# Supplementary material for: An ancestral function of strigolactones as symbiotic rhizosphere signals
Source: Nat Commun. 2022 Jul 8;13:3974. doi: 10.1038/s41467-022-31708-3 (PMC9270392; doi:10.1038/s41467-022-31708-3)
Supplement: Supplementary file 1 — Supplementary Information [file 41467_2022_31708_MOESM1_ESM.pdf]

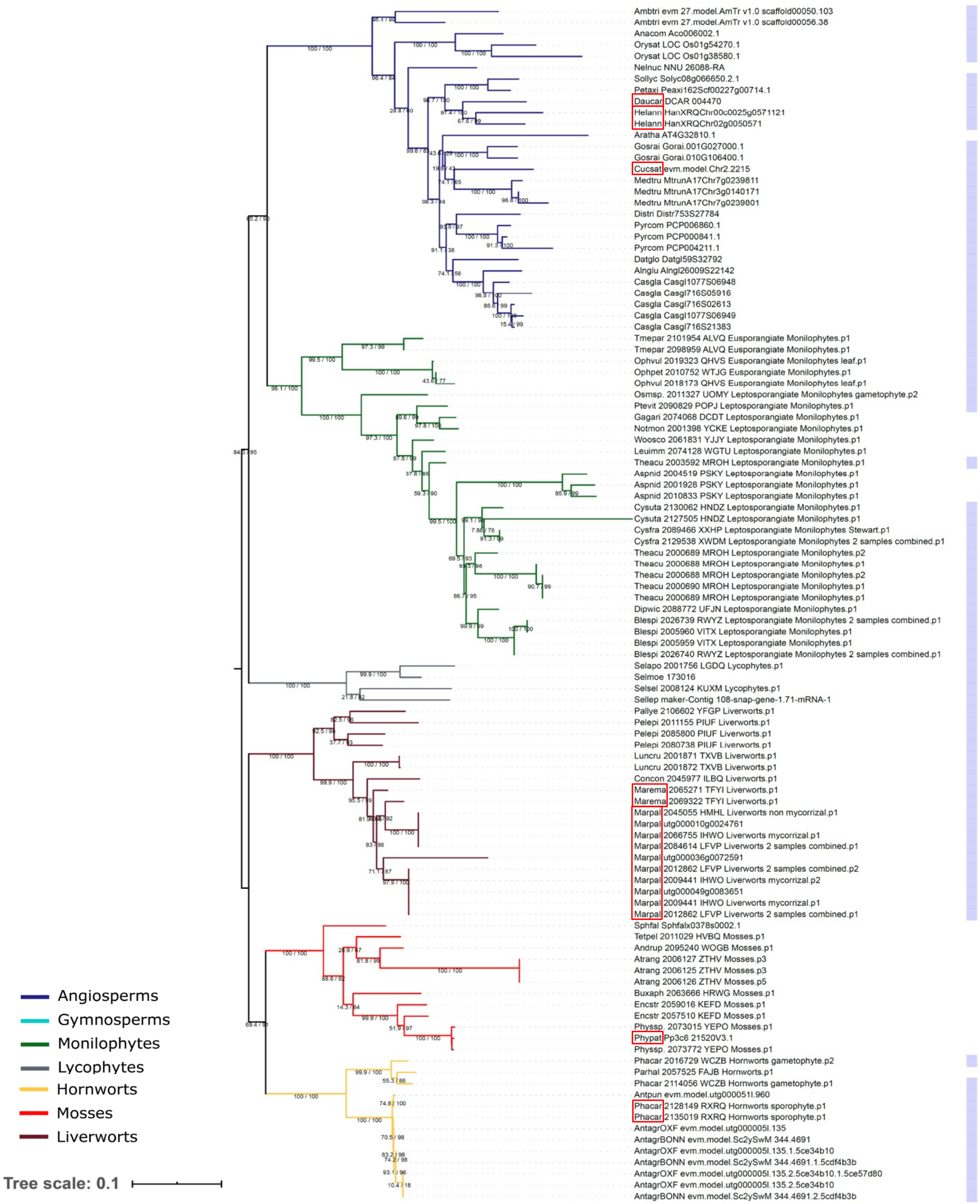

Supplementary Figure 1. Phylogenetic analysis of *CCD8* genes. Tree CCD8: Maximum Likelihood of *CCD8* gene (model: SYM+R5; log-likelihood: -57366.2677). The tree is rooted on the divergence node between vascular and non-vascular plants. Cyan boxes at the right of the tree mark species able to form arbuscular mycorrhizal symbiosis. Branches are coloured according to plant lineages. SH-aLRT and UltraFast Bootstraps branch supports are indicated by the number below the branches on both sides of the "/" symbol. Plants marked with red boxes are analyzed for BSB.

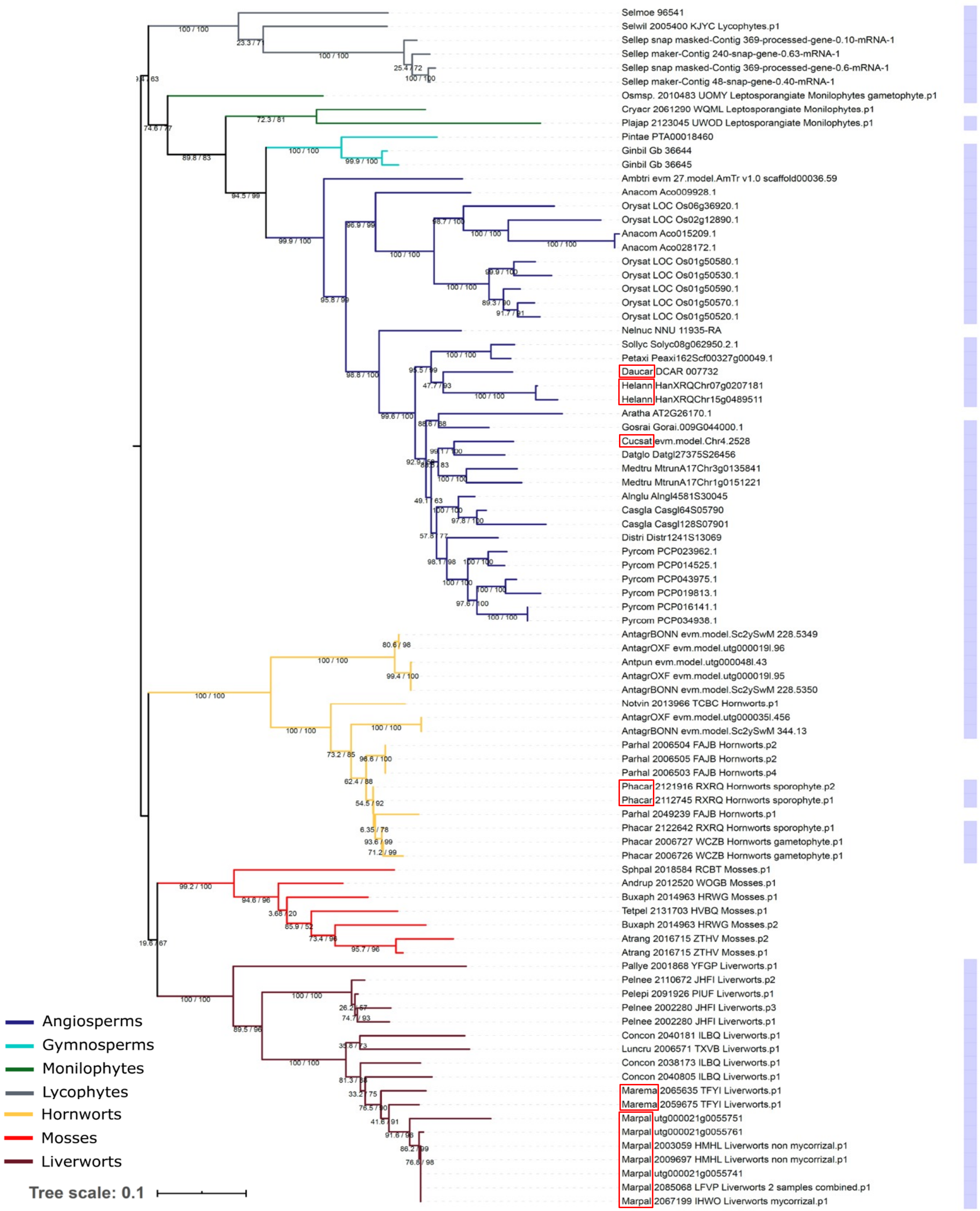

Supplementary Figure 2. Phylogenetic analysis of *MAX1* genes. Maximum Likelihood of *MAX1* gene (model: TVMe+R4; log-likelihood: -55502.0476). The tree is rooted on the divergence node between vascular and non-vascular plants. Cyan boxes at the right of the tree mark species able to form arbuscular mycorrhizal symbiosis. Branches are coloured according to plant. SH-aLRT and UltraFast Bootstraps branch supports are indicated by the number below the branches on both sides of the "/" symbol. Plants marked with red boxes were analyzed for BSB.

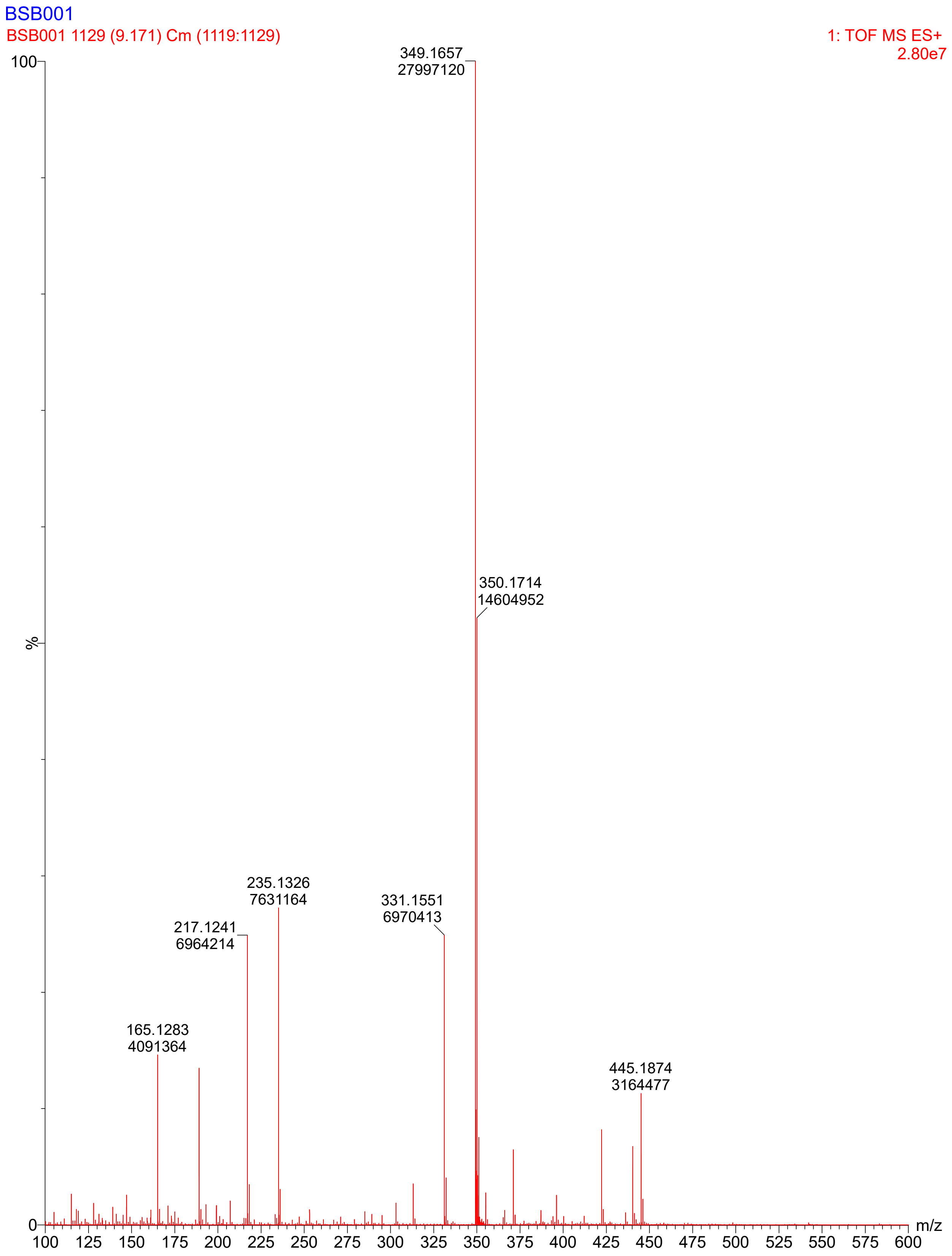

Supplementary Figure 3. High-resolution electrospray ionization mass spectrometry (HR-ESI-MS) data of BSB.

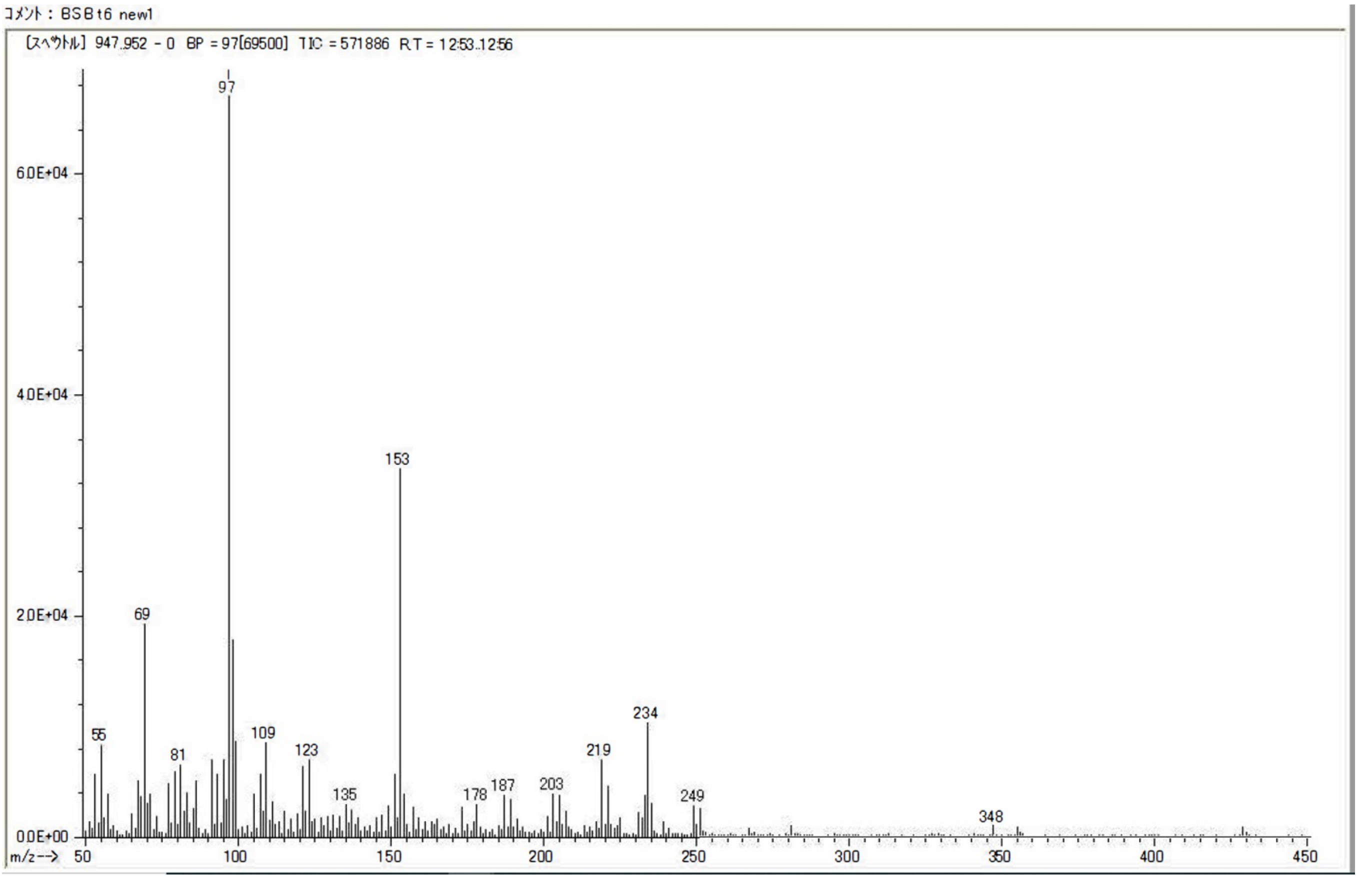

Supplementary Figure 4. Electron impact mass spectrometry (EI-MS) data of BSB.

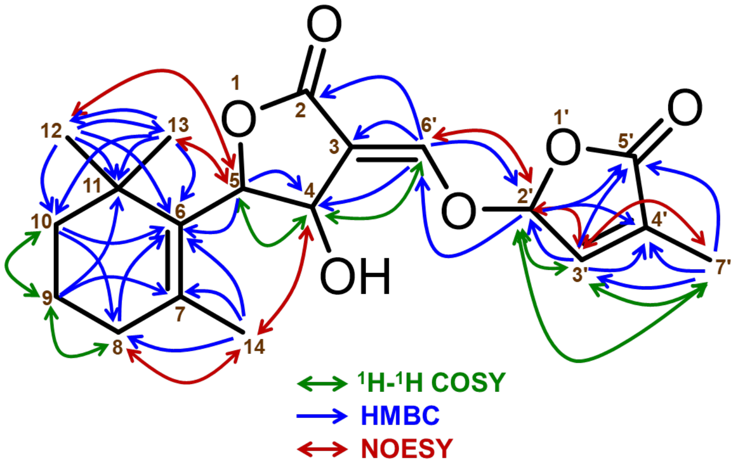

Supplementary Figure 5. ^{1}H-^{1}H correlation spectroscopy (^{1}H-^{1}H COSY), heteronuclear multiple-bond correlation spectroscopy (HMBC), and nuclear Overhauser effect spectroscopy (NOESY) correlations of BSB. ^{1}H-^{1}H COSY (green arrow), HMBC (blue arrow), and NOESY (red arrow)

Default parameters	1	2	3	4
sDP4+ (H data)	89.77%	9.81%	0.41%	0.01%
sDP4+ (C data)	68.75%	31.25%	0.00%	0.00%
sDP4+ (all data)	95.27%	4.73%	0.00%	0.00%
uDP4+ (H data)	56.50%	41.82%	0.46%	1.22%
uDP4+ (C data)	45.95%	54.05%	0.00%	0.00%
uDP4+ (all data)	53.46%	46.54%	0.00%	0.00%
DP4+ (H data)	92.52%	7.48%	0.00%	0.00%
DP4+ (C data)	65.17%	34.83%	0.00%	0.00%
DP4+ (all data)	95.86%	4.14%	0.00%	0.00%

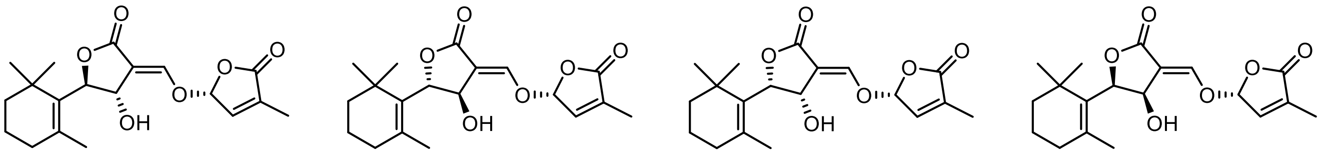

4*S**,5*R**,2'*R**
Isomer 1

4*R**,5*S**,2'*R**
Isomer 2

4*S**,5*S**,2'*R**
Isomer 3

4*R**,5*R**,2'*R**
Isomer 4

Supplementary Figure 6. Detailed DP4+ probability for BSB calculated at PCM/mPW1PW91/6-31+G(d,p) level. Isomers 1, 2, 3, and 4 are (4*S**,5*R**,2'*R**)-, (4*R**,5*S**,2'*R**)-, (4*S**,5*S**,2'*R**)-, and (4*R**,5*R**,2'*R**)-BSB, respectively.

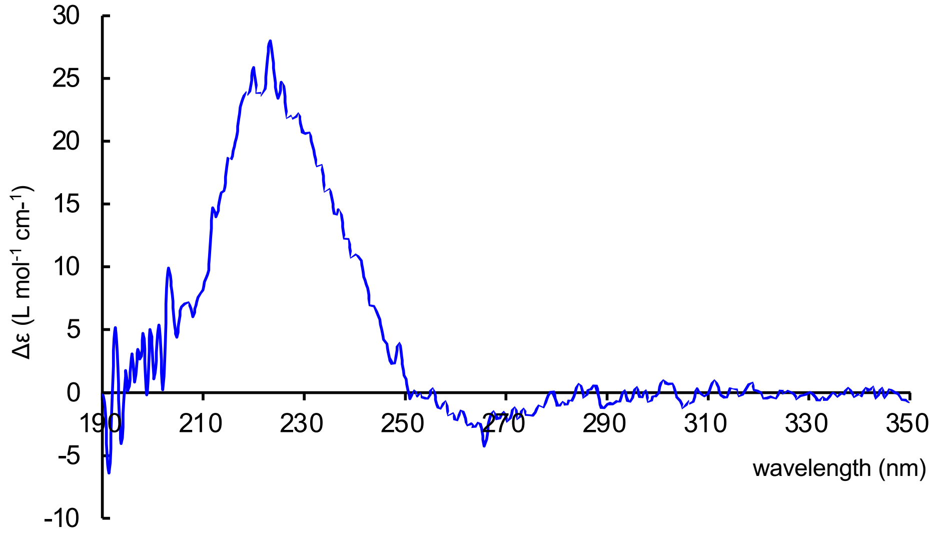

Supplementary Figure. 7. Electronic circular dichroism (ECD) spectrum of BSB. taken in acetonitrile (*c* 0.002).

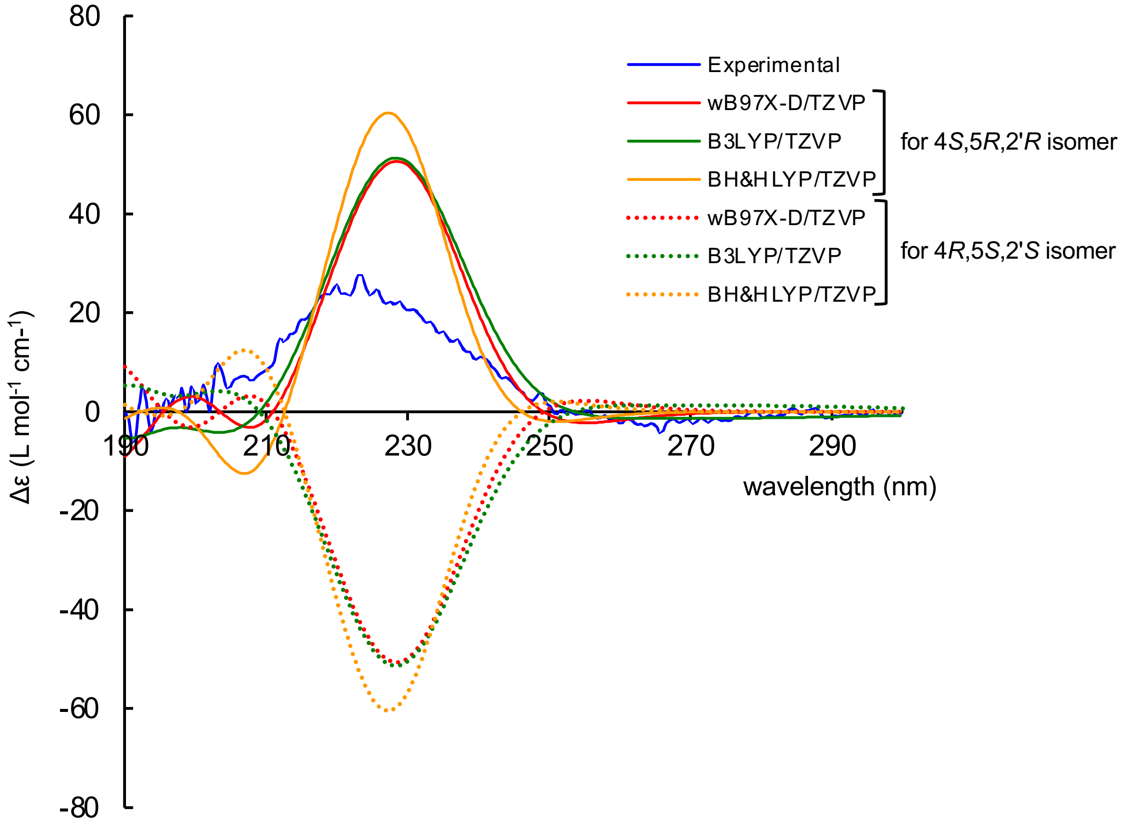

Supplementary Figure 8. Experimental and calculated ECD spectra of (4*S*,5*R*,2'*R*)- and (4*R*,5*S*,2'*S*)-isomers of BSB. UV correction of +10 nm for ωB97X-D/TZVP and +15 nm for BH&HLYP/TZVP were applied for proper comparison.

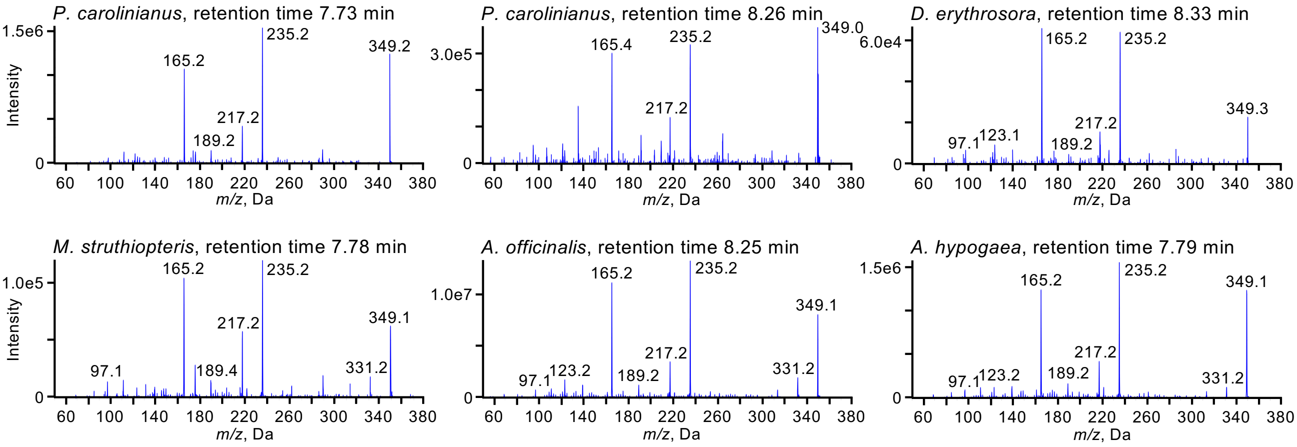

Supplementary Figure 9. Strigolactones in hornwort, ferns and seed plants.

Product ion spectra of BSB. Product ion spectra derived from the precursor ion (*m/z* 349 in positive mode) of peaks detected in hornwort, ferns and seed plants are shown.

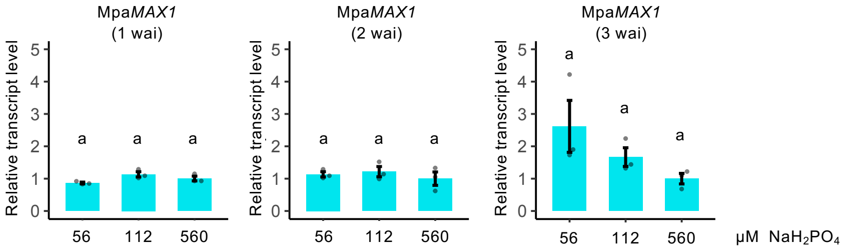

Supplementary Figure 10. Developmental stage-dependent induction of Mpa*MAX1* transcript level in response to low phosphate conditions.
Gemmalings of 1-3 weeks after inoculation (wai) were transferred to media containing different concentrations of phosphate. Data are means ± SD (n = 3 biologically independent samples) and The HSD test was used for multiple comparisons. Statistical differences (p-values < 0.05) are indicated by different letters. Source data provided.

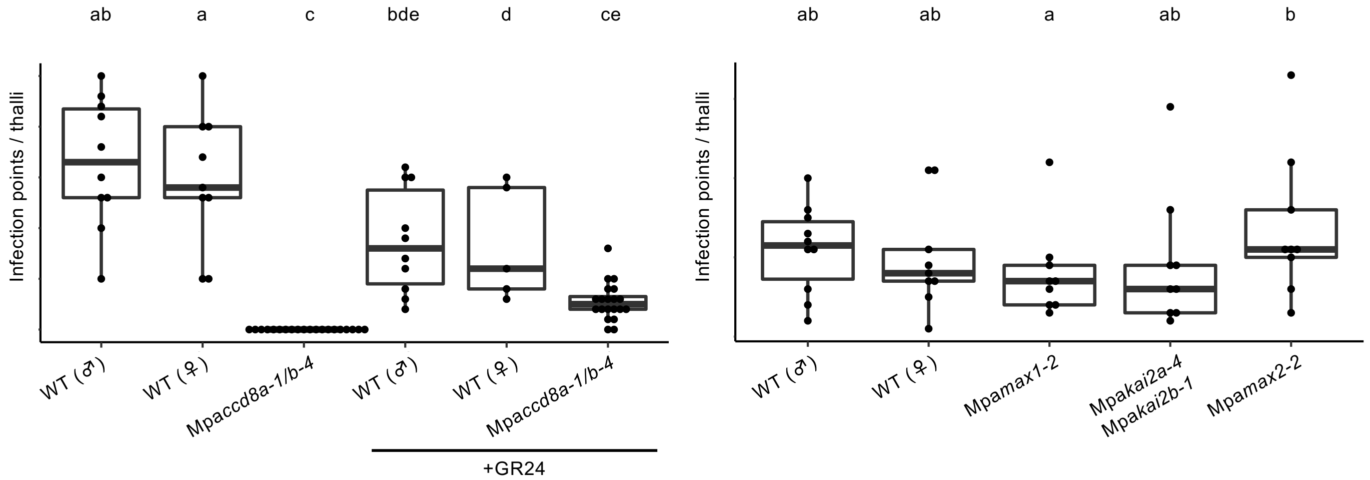

Supplementary Figure 11. Mycorrhizal phenotype of strigolactone biosynthesis and signalling mutants. Replication of experiments shown in Figure 5c conducted in independent mutant alleles is shown. The number of infection points per thalli in BSB biosynthesis and signalling mutants of *M. paleacea* (n ≥ 5 biologically independent plants). Letters show different statistical groups (p-values < 0.05, ANOVA, post hoc Tukey). Box plot shows first quartile, median, third quartile, whiskers 1.5 interquartile. Source data provided.

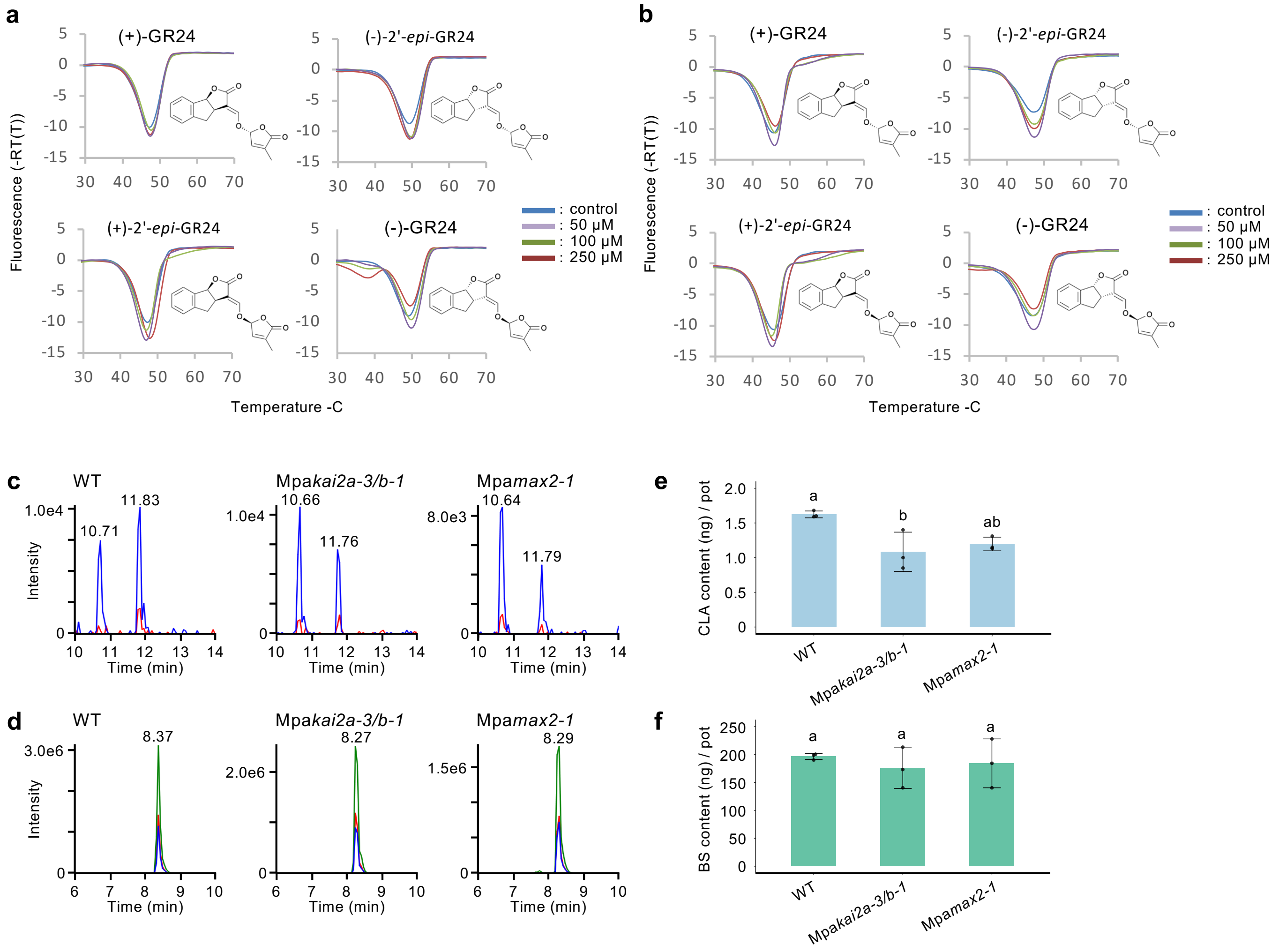

Supplementary Figure 12. Evaluation of the MpaKAI2-SLs interaction.
(a and b) Differential Scanning Fluorimetry (DSF) analysis with 4 stereoisomers of GR24 and MpaKAI2A (a) and MpaKAI2B (b) protein. **(c and d)** Detection of carlactonoic acid and BSB in exudates of the Mpa*kai2a/b* and Mpa*max2* mutants. MRM chromatograms of carlactonoic acid (c) and BSB (d) are shown. **(e and f)** The content of carlactonoic acid and BSB in exudates of the Mpa*kai2a/b* and Mpa*max2* mutants. The exudates of WT, Mpa*kai2a/b* and Mpa*max2* grown in 10 cm diameter pots were analysed by LC-MS/MS. Carlactonoic acid (e) was quantified using [1-$^{13}CH_3$] carlactonoic acid as an internal standard. BSB (f) was quantified using [$^{2}H_6$]4DO as an internal standard. Data in (e) and (f) are the means ± SD (n = 3 biologically independent samples, ANOVA with Tukey's test, p-values < 0.05). Source data provided.

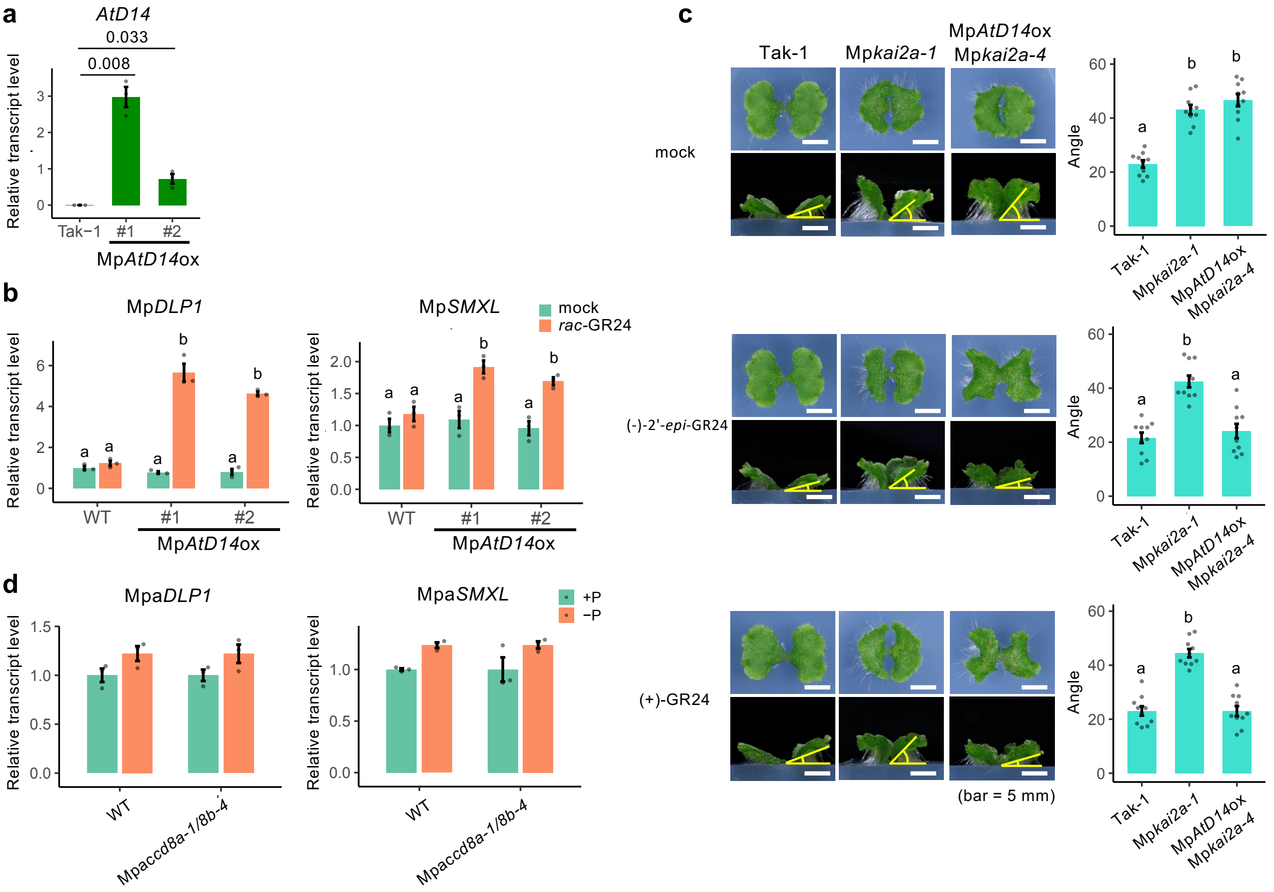

Supplementary Figure 13. Effects of SLs and phosphorus on gene transcript level in *AtD14* line in *M. polymorpha* and in *M. paleacea.*

(a) Transcript level of *AtD14* introduced into *M. polymorpha*. Data are means ± SD (n = 3 biologically independent samples), and p-values in Welch's t-test, two tailed are indicated. **(b)** Transcript level of Mp*DLP1* and Mp*SMXL*, markers of the KAI2-dependent signaling pathway, in WT and Mp*AtD14*ox lines of *M. polymorpha* after *rac*-GR24 treatment. Data are means ± SD (n = 3 biologically independent samples). **(c)** Complementation of Mp*kai2a* phenotypes in Mp*AtD14*ox/Mp*kai2a* (*M. polymorpha*) line by the addition of 1 μM (-)-2'-*epi*-GR24 or 1 μM (+)-GR24. Data are means ± SD (n = 10 biologically independent plants). **(d)** Transcript level of Mpa*DLP1* and Mpa*SMXL* after phosphate starvation in WT and Mpa*ccd8a-1/8b-4*. Data are means ± SD (n = 3 biologically independent samples). The HSD test was used for multiple comparisons and statistical differences (P-values < 0.05) are indicated by different letters in (a) to (d). Source data provided.

Supplementary Table 1. Production of CRISPR mutants.

Allele	gRNA sequence	Mutation site	Amino acid
Mp*akai2a-1*	GGACTGACCAGTCTGTGTGGA	c.93_94insT	p.Trp32LeufsTer11
Mp*akai2a-2*	TGTACGATACCATGGGAGCA	c.157_158delinsA	p.Ala53LysfsTer28
Mp*akai2a-3*	TGTACGATACCATGGGAGCA	c.156_157del	p.Ala53ArgfsTer22
Mp*akai2a-4*	TGTACGATACCATGGGAGCA	c.156del	p.Ala53GlnfsTer28
Mp*akai2a-5*	GGACTGACCAGTCTGTGTGGA	c.93_94insT	p.Trp32LeufsTer11
Mp*kai2a-4*	GGAGATCAGCTCGTGGTACT	c.55_59del	p.Val19ThrfsTer8
Mp*akai2b-1*	TTGGCACGGATCAGTCAGTG	c.88_91delinsAT	p.Ser30IlefsTer19
Mp*akai2b-2*	TTGGCACGGATCAGTCAGTG	c.1-104_117delins CAGGTGAGGTATAAGAT GCTTCCAAACA	No start codon
Mp*accd8a-1*	GGAACAGTGGGAAGGAGAGC	c.389_426+123del	p.Leu132GlufsTer16
Mp*accd8a-2*	GTTCACAATAGCAGATAAAG	c.53_56del	p.Asp158LysfsTer16
Mp*accd8b-1*	ACAATGGGAAGGAGAGCTGG	c.368_380delins AAATTCCACCGAGGAAT TGAGCCCTGGAC	p.Leu123GlnfsTer7
Mp*accd8b-2*	ACAATGGGAAGGAGAGCTGG	c.367_368insT	p.Glu124GlyfsTer27
Mp*accd8b-3*	ACAATGGGAAGGAGAGCTGG	c.365_369delins GAACTCAGGAATT	p.Glu122GlyfsTer19
Mp*accd8b-4*	ACAATGGGAAGGAGAGCTGG	c.366_367insC	p.Leu123ProfsTer28
Mp*amax1-1*	CTCTGCTAACCAAGCACC	c.157_63del	p.Lys54ArgfsTer19
Mp*amax1-2*	ATGCAGAGCTGTGCCGAC	c.780_806del	p.Asp85ValfsTer428
Mp*amax2-1*	ACCCAAATCAGCGACCTGC	c.19_58del	p.Aps11_Ile20delinsVal
Mp*amax2-2*	ACCCAAATCAGCGACCTGC	c.34_35del	p.Pro13ArafsTer19

※ Notations are followed den Dunnen JT and Antonarakis SE (2000). Hum.mutat. 15:7-12

Supplementary Table 2. NMR spectroscopic data for BSB.*

No.	δ_C	δ_H (mult., *J* Hz)	HMQC & DEPT	^1H–^1H COSY	HMBC	NOESY
2	170.05		C			
3	113.01		C			
4	72.16	5.08 (dd, 4.6, 1.7)	CH	H-5, H-6'	none	H-14
5	84.20	4.87 (d, 4.6)	CH	H-4	C-4, C-6	H-12, H-13
6	134.88		C			
7	134.97		C			
8	33.95	1.986 (t, 6.1), 1.991 (t, 6.0)	CH_2	H-9	C-6	H-14
9	19.10	1.57-1.63 (m)	CH_2	H-8, H-10	C-7, C-11	
10	39.48	1.44-1.51 (m)	CH_2	H-9	C-6, C-8	
11	34.55		C			
12	28.50	1.06 (s)	CH_3		C-6, C-10, C-11, C-13	H-5
13	27.86	1.10 (s)	CH_3		C-6, C-10, C-11, C-12	H-5
14	20.75	1.51 (s)	CH_3		C-6, C-7, C-8	H-4, H-8
2'	100.37	6.22 (quin, 1.4)	CH	H-3', H-7'	C-4', C-5', C-6'	H-3', H-6', H-7'
3'	140.57	6.94 (quin, 1.7)	CH	H-2', H-7'	C-2', C-4',C-5'	H-2', H-7'
4'	136.39		C			
5'	169.88		C			
6'	153.25	7.59 (d, 1.7)	CH	H-4	C-2, C-3, C-4, C-2'	H-2'
7'	10.79	2.04 (br.t, 1.7)	CH_3	H-2', H-3'	C-3', C-4', C-5'	H-2', H-3'

* taken at 500 MHz for ^1H and at 125 MHz for ^{13}C, in $CDCl_3$ (δ_H 7.26, δ_C 77.0).

Supplementary Table 3. Species used for BSB analysis in this study.

Species name	Group	Order	Family
*Marchantia paleacea**	Liverworts	Marchantiales	Marchantiaceae
*Marchantia pinnata**	Liverworts	Marchantiales	Marchantiaceae
*Marchantia emarginata**	Liverworts	Marchantiales	Marchantiaceae
Marchantia polymorpha	Liverworts	Marchantiales	Marchantiaceae
Physcomitrium patens	Mosses	Funariales	Funariaceae
*Phaeoceros carolinianus**	Hornworts	Notothyladales	Notothyladaceae
*Dryopteris erythrosora**	Leptosporangiate fern	Polypodiales	Dryopteridaceae
Polystichum tripteron	Leptosporangiate fern	Polypodiales	Dryopteridaceae
*Matteuccia struthiopteris**	Leptosporangiate fern	Polypodiales	Onocleaceae
Adiantum pedatum	Leptosporangiate fern	Polypodiales	Pteridaceae
Pteris multifida	Leptosporangiate fern	Polypodiales	Pteridaceae
Aspleniaceae scolopendrium	Leptosporangiate fern	Polypodiales	Aspleniineae
Blechnum nipponicum	Leptosporangiate fern	Polypodiales	Blechnaceae
Equisetum hyemale	Eusporangiate fern	Equisetales	Equisetaceae
Equisetum arvense	Eusporangiate fern	Equisetales	Equisetaceae
*Asparagus officinalis**	Angiosperms	Asparagales	Asparagaceae
Allium fistulosum	Angiosperms	Asparagales	Amaryllidaceae
*Arachis hypogaea**	Angiosperms	Fabales	Fabaceae
Cucumis sativus	Angiosperms	Cucurbitales	Cucurbitaceae
Cucumis melo	Angiosperms	Cucurbitales	Cucurbitaceae
Citrullus lanatus	Angiosperms	Cucurbitales	Cucurbitaceae
Cucurbita maxima	Angiosperms	Cucurbitales	Cucurbitaceae
Solanum melongena	Angiosperms	Solanales	Solanaceae
Capsicum annuum	Angiosperms	Solanales	Solanaceae
Arctium lappa	Angiosperms	Asterales	Asteraceae
Lactuca sativa	Angiosperms	Asterales	Asteraceae
Helianthus annuus	Angiosperms	Asterales	Asteraceae
Phaseolus vulgaris	Angiosperms	Fabales	Fabaceae
Petroselinum crispum	Angiosperms	Apiales	Apiaceae
Daucus carota	Angiosperms	Apiales	Apiaceae
Apium graveolens	Angiosperms	Apiales	Apiaceae
Brassica rapa	Angiosperms	Brassicales	Brassicaceae
Tropaeolum majus	Angiosperms	Brassicales	Tropaeolum
Abelmoschus esculentus	Angiosperms	Malvales	Malvaceae
Bombax ceiba	Angiosperms	Malvales	Malvaceae
Ocimum basilicum	Angiosperms	Lamiales	Lamiaceae
Lavandula angustifolia	Angiosperms	Lamiales	Lamiaceae
Antirrhinum majus	Angiosperms	Lamiales	Plantaginaceae
Viola × wittrockiana	Angiosperms	Malpighiales	Violaceae
Eustoma grandiflorum	Angiosperms	Gentianales	Gentianaceae
Myosotis scorpioides	Angiosperms	Boraginales	Boraginaceae
Dianthus superbus	Angiosperms	Caryophyllales	Caryophyllaceae

*BSB was detected.

Supplementary Table 4. Oligonucleotide primers used in this study.

Use	Name	Sequence (5'→3')
Mutagenesis by CRISPR	MpaKAI2ACR1F	GCACCCAGCCTCTCGGACTGACCAGTCTGTGTGGAGTTTTAGAGCTAGAA
	MpaKAI2ACR1R	TTCTAGCTCTAAAACTCCACACAGACTGGTCAGTCCGAGAGGCTGGGTGC
	MpaKAI2ACR2F	GCACCCAGCCTCTCGTGTACGATACCATGGGAGCAGTTTTAGAGCTAGAA
	MpaKAI2ACR2R	TTCTAGCTCTAAAACTGCTCCCATGGTATCGTACACGAGAGGCTGGGTGC
	MpaKAI2BCR1F	GCACCCAGCCTCTCGTTGGCACGGATCAGTCAGTGGTTTTAGAGCTAGAA
	MpaKAI2BCR1R	TTCTAGCTCTAAAACCACTGACTGATCCGTGCCAACGAGAGGCTGGGTGC
	MpaCCD8ACR1F	GCACCCAGCCTCTCGGGAACAGTGGGAAGGAGAGCGTTTTAGAGCTAGAA
	MpaCCD8ACR1R	TTCTAGCTCTAAAACGCTCTCCTTCCCACTGTTCCCGAGAGGCTGGGTGC
	MpaCCD8ACR2F	GCACCCAGCCTCTCGGTTCACAATAGCAGATAAAGGTTTTAGAGCTAGAA
	MpaCCD8ACR2R	TTCTAGCTCTAAAACCTTTATCTGCTATTGTGAACCGAGAGGCTGGGTGC
	MpaCCD8BCR1F	GCACCCAGCCTCTCGACAATGGGAAGGAGAGCTGGGTTTTAGAGCTAGAA
	MpaCCD8BCR1R	TTCTAGCTCTAAAACCCAGCTCTCCTTCCCATTGTCGAGAGGCTGGGTGC
	MpaMAX1CR1F	CTCGCTCTGCTAACCAAGCACC
	MpaMAX1CR1R	AAACGGTGCTTGGTTAGCAGAG
	MpaMAX1CR2F	CTCGATGCAGAGCTGTGCCGAC
	MpaMAX1CR2R	AAACGTCGGCACAGCTCTGCAT
	MpaMAX2CR1F	CTCGACCCAAATCAGCGACCTGC
	MpaMAX2CR1R	AAACGCAGGTCGCTGATTTGGGT
Genotyping	MpaKAI2AgeF	CAATGTGCGGATAGTTGGTTCG
	MpaKAI2AgeR	TCTCAATAGATGCAAGGCACCC
	MpaKAI2BgeF	ACGTGTAGTAGGATCAGGGG
	MpaKAI2BgeR	GTCCGGTCTTTCCAAGGATG
	MpaCCD8AgeF	AGGAGAAAGCTGTAGAGATCGA
	MpaCCD8AgeR	CTCAGTTGGTTGTGAGATGCTA
	MpaCCD8BgeF	CACCCAAGTCCAGTGATTCG
	MpaCCD8BgeR	TCCCTCAAACTTCAAACGCA
	MpaMAX1geF	ATGGGGAGAGTTCGGCAGA
	MpaMAX1geR	CTGTCGTCTTAAAGCCCTCTG
	MpaMAX2geF	ATGGGGAGAGTTCGGCAGA
	MpaMAX2geR	CTGTCGTCTTAAAGCCCTCTG
qPCR	MpACTINqPCRF	AGGCATCTGGTATCCACGAG
	MpACTINqPCRR	ACATGGTCGTTCCTCCAGAC
	MpDLP1qPCRF	GGTGTGAAGAAAGTTGGAGTTATGG
	MpDLP1qPCRR	GTGTGAGGAATGAGGGATGGTT
	MpSMXLqPCRF	TGGGATGTCAGGTCGGAAAC
	MpSMXLqPCRR	AAAAGTTCTGCTGAGCTGCG
	MpaACTINqPCRF	ACGTGGCAATTCAGGCTGTC
	MpaACTINqPCRR	GCGTGGGGAAGAGCATAACC
	MpaDLP1qPCRF	ACCATCCGTCATTCCTCACAC
	MpaDLP1qPCRR	ATTTGTCTCCAATCTCAGGGTTCTT
	AtD14qPCRF	GCTTCGGTGGCGGAGTATCT
	AtD14qPCRR	AGGATGTTTCTGTGCCGGCT
	MpaD27qPCRF	GTACAAGGACTCTTGGCTTGAGAAA
	MpaD27qPCRR	GGAGCAAGTCGGAATGTGAAG
	MpaCCD7qPCRF	GAACCTGGAGAAGCAGGCTT
	MpaCCD7qPCRR	GGGGACTCTCAGTCTAGCCA
	MpaCCD8AqPCRF	CTATGGCTGGCTGGGACATATTTG
	MpaCCD8AqPCRR	CTCTTCGCAGCATTGTACGC
	MpaCCD8BqPCRF	CTGCACTTGACCTTCCCGATG
	MpaCCD8BqPCRR	GTGGGAGGACAATCTTGGGG
	MpaMAX1qPCRF	ACATCGAGCTGGGTGGCTAC
	MpaMAX1qPCRR	TCGCTCGGGTCGAAATTCCT
	MpaSMXLqPCRF	TGCCACCATCAAGCACACCT
	MpaSMXLqPCRR	GAACAACGGGCTTTGCGTCA
	AaACTINqPCRF	GGCATCACACTTTCTACAATGAGC
	AaACTINqPCRR	TGACACCATCACCAGAATCAAGC
	AaCCD8qPCRF	TGTCAAGCAGCCGAGATGG
	AaCCD8qPCRR	GTGGAGTACCCGTCAAAGAGG
Cloning	MpCCD8-F	GAACAGCGTGAARCAGGAAC
	MpCCD8-R	GGRTTGGCTGTGTGTTCACAACA
	MpaKAI2A_cDNA_F	ATCACCATCACCATATGTCTATCCTTGAGGCTCAC
	MpaKAI2A_cDNA_R	GGTGGTGGTGCTCGAGCTAAATAGAACCCATAATA
	MpaKAI2B_cDNA_F	ATCACCATCACCATATGTCTAACCTCGAAGTTCAT
	MpaKAI2B_cDNA_R	GGTGGTGGTGCTCGAGTCAGCTCATTGCCACGTGC
	MpaMAX1_cDNA_F	GAGTTGCAGTTCTGAACGTG
	MpaMAX1_cDNA_R	CTGTAATCCTGTTCGTTCGTC
	MpaMAX1_cloning_F	CTAAATTACCGGATCATGATAACGAGCATGGAAGGATG
	MpaMAX1_cloning_R	GCGAATTCGAGCTCGCTACAAGCGTGTCCATCTGG

Supplementary Table 5. Accessions No. of genes analyzed in this study

Name	species	Accession
MpaKAI2A	*M. paleacea*	Marpal_utg000015g0039791
MpaKAI2B	*M. paleacea*	Marpal_utg000147g0178301
MpaMAX2	*M. paleacea*	Marpal_utg000086g0138511
MpaD27	*M. paleacea*	Marpal_utg000152g0180621
MpaCCD7	*M. paleacea*	Marpal_utg000013g0034951
MpaCCD8A	*M. paleacea*	Marpal_utg000010g0024761
MpaCCD8B	*M. paleacea*	Marpal_utg000049g0083651
MpaMAX1	*M. paleacea*	Marpal_utg000021g0055741
MpaSMXL	*M. paleacea*	Marpal_scaff7741_FGenesh6.1
MpaDLP1	*M. paleacea*	Marpal_utg000067g0111431
MpaACTIN	*M. paleacea*	Marpal_utg000003g0005821
MpKAI2A	*M. paleacea*	Mp2g11710.1
MpDLP1	*M. polymorpha*	Mp5g09160
MpSMXL	*M. polymorpha*	Mp3g06310.1
MpACTIN	*M. polymorpha*	Mp6g11010
AaCCD8	*A. agrestis*	AagrBONN_evm.model.Sc2ySwM_344.4691.1
AtD14	*A. thaliana*	NP_566220.1

Supplementary Method 1. Computational NMR calculation details.

Conformational searches were performed with the MMFF force field (Merck Molecular force field) using the Monte Carlo algorithm implemented in Spartan'20,[1] using default parameters and convergence criteria. All the quantum mechanical calculations were performed using Gaussian09.[2] All conformers within 5 kcal/mol (20.9 kJ/mol) of the lowest energy conformer were subjected to reoptimization at the B3LYP/6-31G* level in gas. Frequency calculations were done for all optimized geometries at the B3LYP/6-31G* level to determine the nature of the stationary point found, and to compute the thermochemical properties (at 1 atm and 298.15 K). The B3LYP/6-31G* optimized geometries within 2 kcal/mol (8.37 kJ/mol) of the lowest energy conformer were next subjected to NMR calculations. The magnetic shielding constants (σ) were computed using the gauge including atomic orbitals (GIAO) method at PCM/mPW1PW91/6-31+G** level of theory, using the polarizable continuum model, PCM, with chloroform as the solvent. The calculated shielding tensors of conformers were averaged according to the Boltzmann distribution theory and their relative Gibbs free energy. The DP4+ probability, the unscaled and scaled chemical shifts, and the unscaled and scaled errors were calculated using the Sarotti's Excel spreadsheet available for free at sarotti-nmr.weebly.com.[3,4]

References:

1) Spartan'20; Wavefunction: Irvine, CA.

2) Frisch, M. J.; Trucks, G. W.; Schlegel, H. B.; Scuseria, G. E.; Robb, M. A.; Cheeseman, J. R.; Scalmani, G.; Barone, V.; Mennucci, B.; Petersson, G. A.; Nakatsuji, H.; Caricato, M.; Li, X.; Hratchian, H. P.; Izmaylov, A. F.; Bloino, J.; Zheng, G.; Sonnenberg, J. L.; Hada, M.; Ehara, M.; Toyota, K.; Fukuda, R.; Hasegawa, J.; Ishida, M.; Nakajima, T.; Honda, Y.; Kitao, O.; Nakai, H.; Vreven, T.; Montgomery, J. A., Jr., Peralta, J. E.; Ogliaro, F.; Bearpark, M.; Heyd, J. J.; Brothers, E.; Kudin, K. N.; Staroverov, V. N.; Kobayashi, R.; Normand, J.; Raghavachari, K.; Rendell, A.; Burant, J. C.; Iyengar, S. S.; Tomasi, J.; Cossi, M.; Rega, N.; Millam, J. M.; Klene, M.; Knox, J. E.; Cross, J. B.; Bakken, V.; Adamo, C.; Jaramillo, J.; Gomperts, R.; Stratmann, R. E.; Yazyev, O.; Austin, A. J.; Cammi, R.; Pomelli, C.; Ochterski, J. W.; Martin, R. L.; Morokuma, K.; Zakrzewski, V. G.; Voth, G. A.; Salvador, P.; Dannenberg, J. J.; Dapprich, S.; Daniels, A. D.; Farkas, O.; Foresman, J. B.; Ortiz, J. V.; Cioslowski, J.; Fox, D. J. Gaussian 09, Gaussian, Inc.: Wallingford, CT, 2009.

3) Grimblat, N.; Zanardi, M. M.; Sarotti, A. M. *J. Org. Chem.* 2015, **80**, 12526−12534.

4) Zanardi, M. M.; Sarotti, A. M. *J. Org. Chem.*, 2021, **86**, 8544–8548.

Supplementary Method 2. Computational ECD calculation details.

All conformers within 5 kcal/mol (20.9 kJ/mol) of the lowest energy conformer obtained in the NMR calculation were reoptimized at the ωB97X-D/TZVP level using IEF-PCM solvent model for acetonitrile. All optimized structures were confirmed to be local minima (no imaginary frequencies) by calculating the harmonic frequencies at the same level of theory. TDDFT calculations were run using several combinations of functionals (ωB97X-D, B3LYP, BH&HLYP) and the TZVP basis set in acetonitrile (IEF-PCM); they included at least 30 excited states. Boltzmann weights were computed using relative Gibbs free energies. ECD spectra were generated by applying a Gaussian band shape with 0.25 eV exponential halfwidth.
