# Supplementary material for: An ancestral function of strigolactones as symbiotic rhizosphere signals
Source: Nat Commun. 2022 Jul 8;13:3974. doi: 10.1038/s41467-022-31708-3 (PMC9270392; doi:10.1038/s41467-022-31708-3)
Supplement: Supplementary file 3 — Description of Additional Supplementary Files [file 41467_2022_31708_MOESM3_ESM.pdf]

Description of Additional Supplementary Files

File Name: Supplementary Data 1
Description: Species used in the analysis shown in Supplementary Figs 1 and 2.

File Name: Supplementary Data 2
Description: Chemical profiles of BSB.

File Name: Supplementary Data 3
Description: Results of RNAseq analysis.
