# Supplementary material for: An ancestral function of strigolactones as symbiotic rhizosphere signals
Source: Nat Commun. 2022 Jul 8;13:3974. doi: 10.1038/s41467-022-31708-3 (PMC9270392; doi:10.1038/s41467-022-31708-3)
Supplement: Supplementary file 5 — Supplementary Data 2 [file 41467_2022_31708_MOESM5_ESM.pdf]

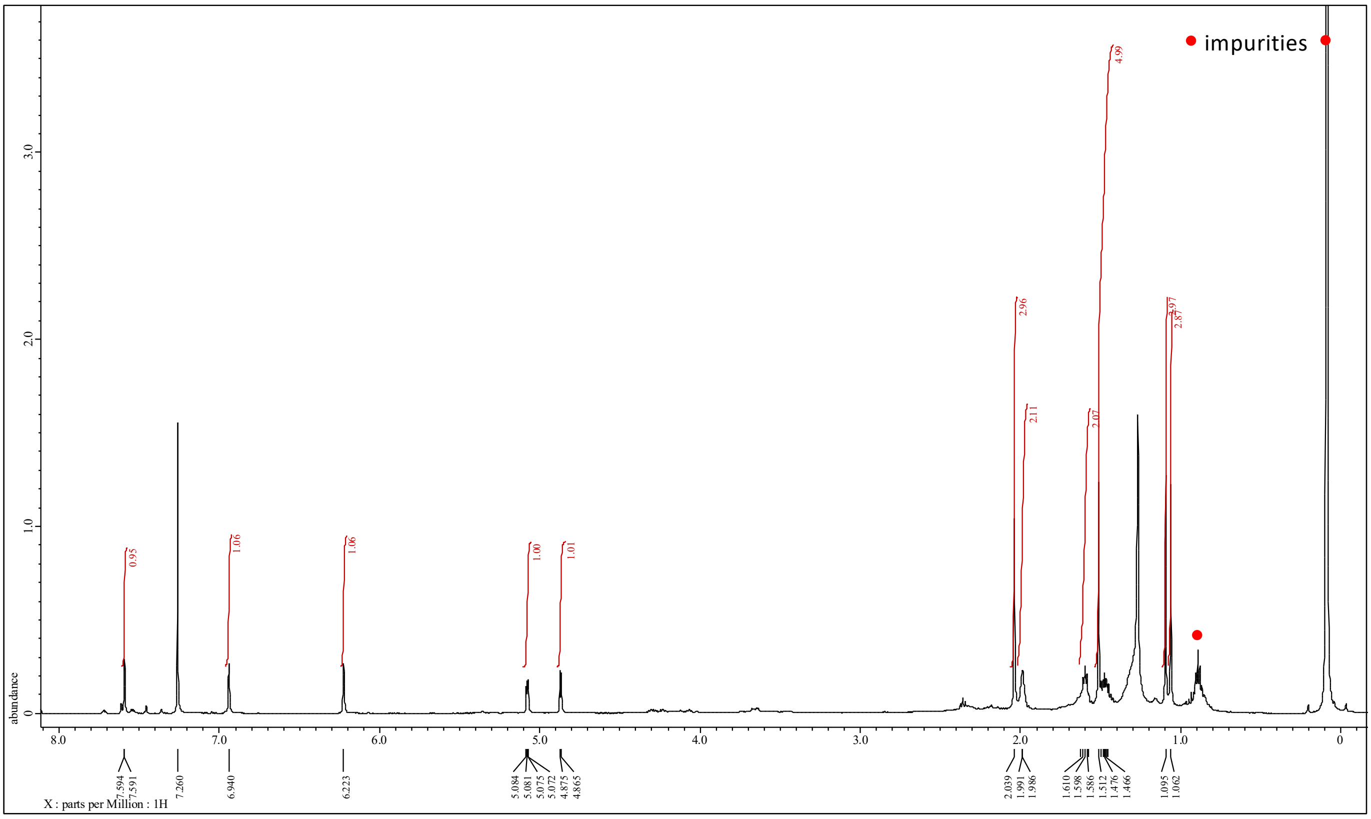


**Supplementary Data 2.1. 1-Dimensional proton nuclear magnetic resonance spectroscopy (1D $^{1}H$-NMR) spectrum of BSB (500 MHz, $CDCl_3$).**

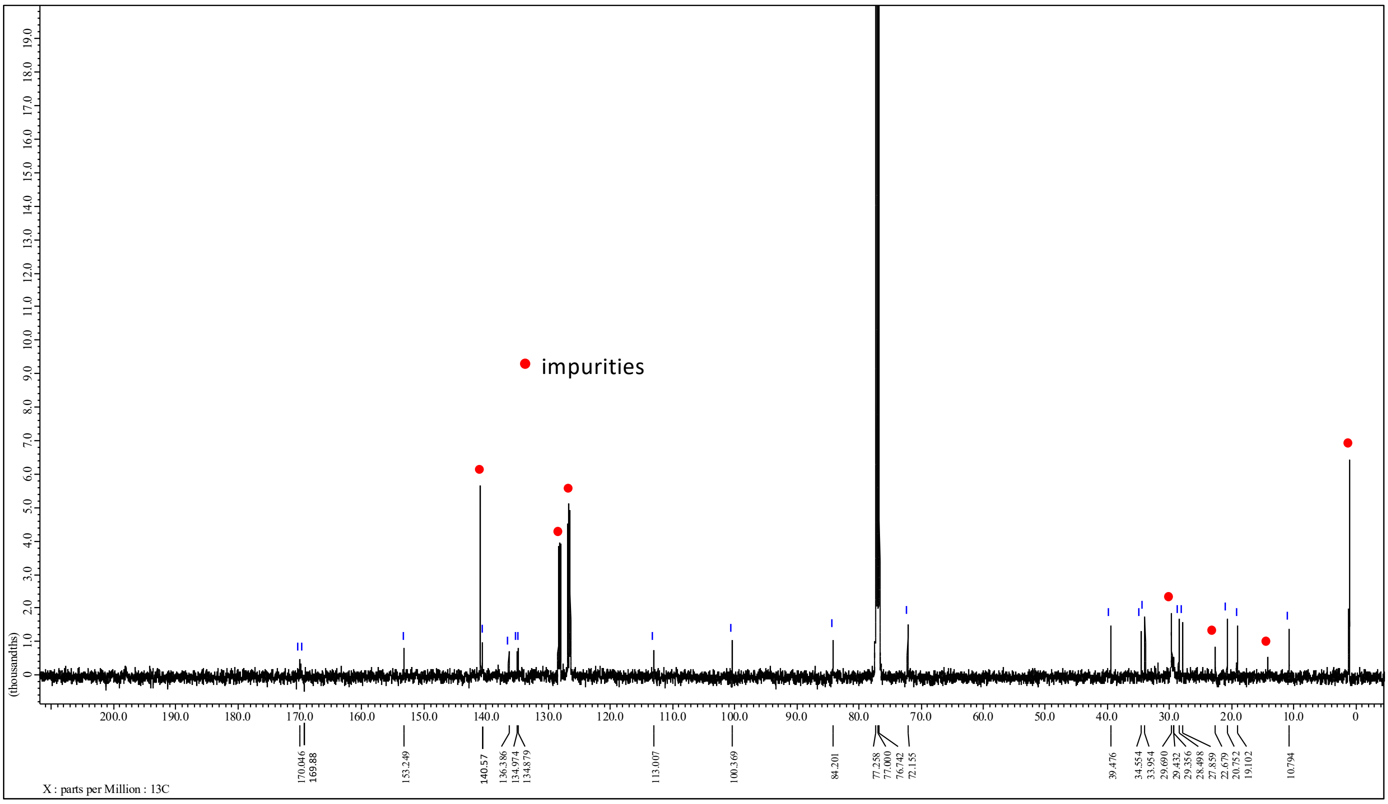


**Supplementary Data 2.2. 1-Dimensional carbon-13 nuclear magnetic resonance spectroscopy (1D $^{13}$C-NMR) spectrum of BSB (125 MHz, $CDCl_3$).**

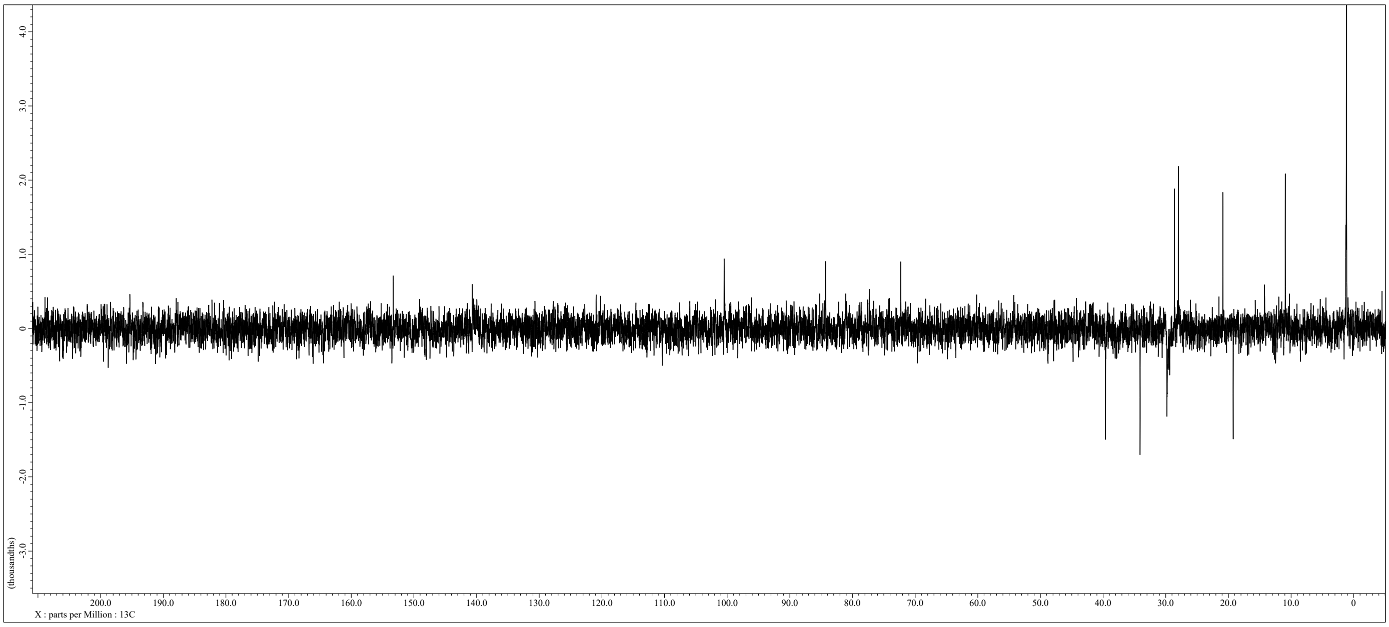


**Supplementary Data 2.3. Distortionless enhancement by polarization transfer (DEPT135) spectrum of BSB (125 MHz, $CDCl_3$).**

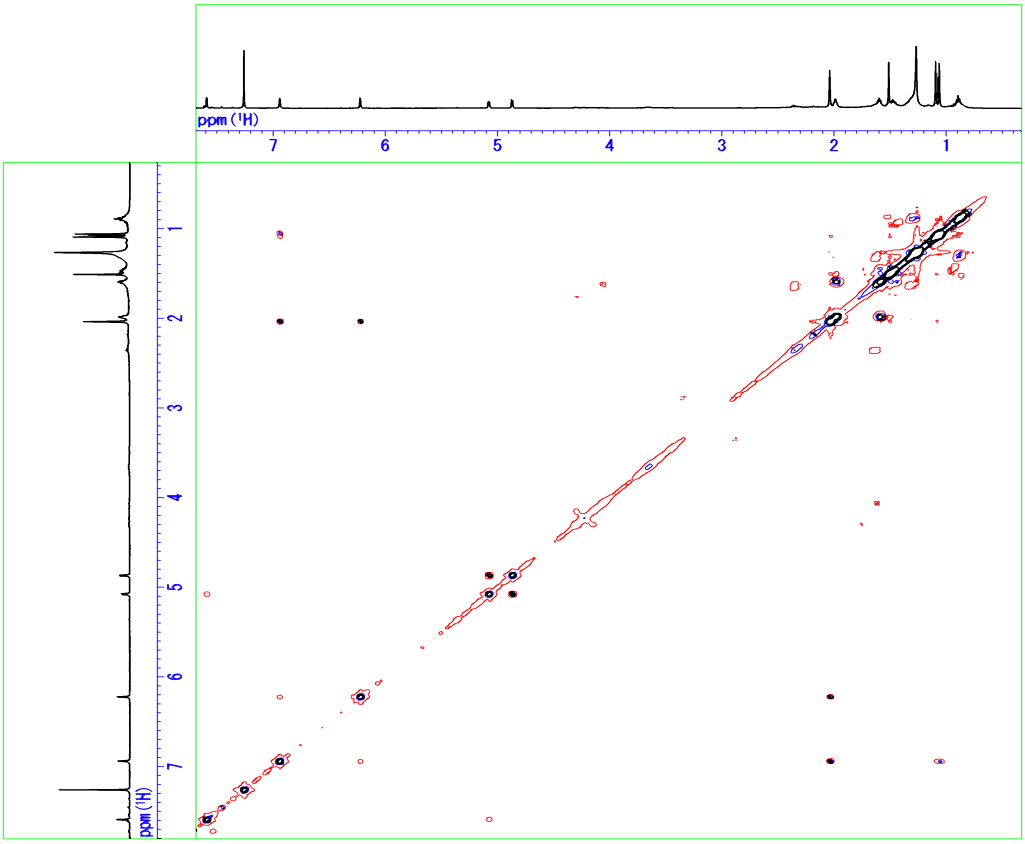


**Supplementary Data 2.4. $^{1}H$-$^{1}H$ correlation spectroscopy ($^{1}H$-$^{1}H$ COSY) spectrum of BSB.**

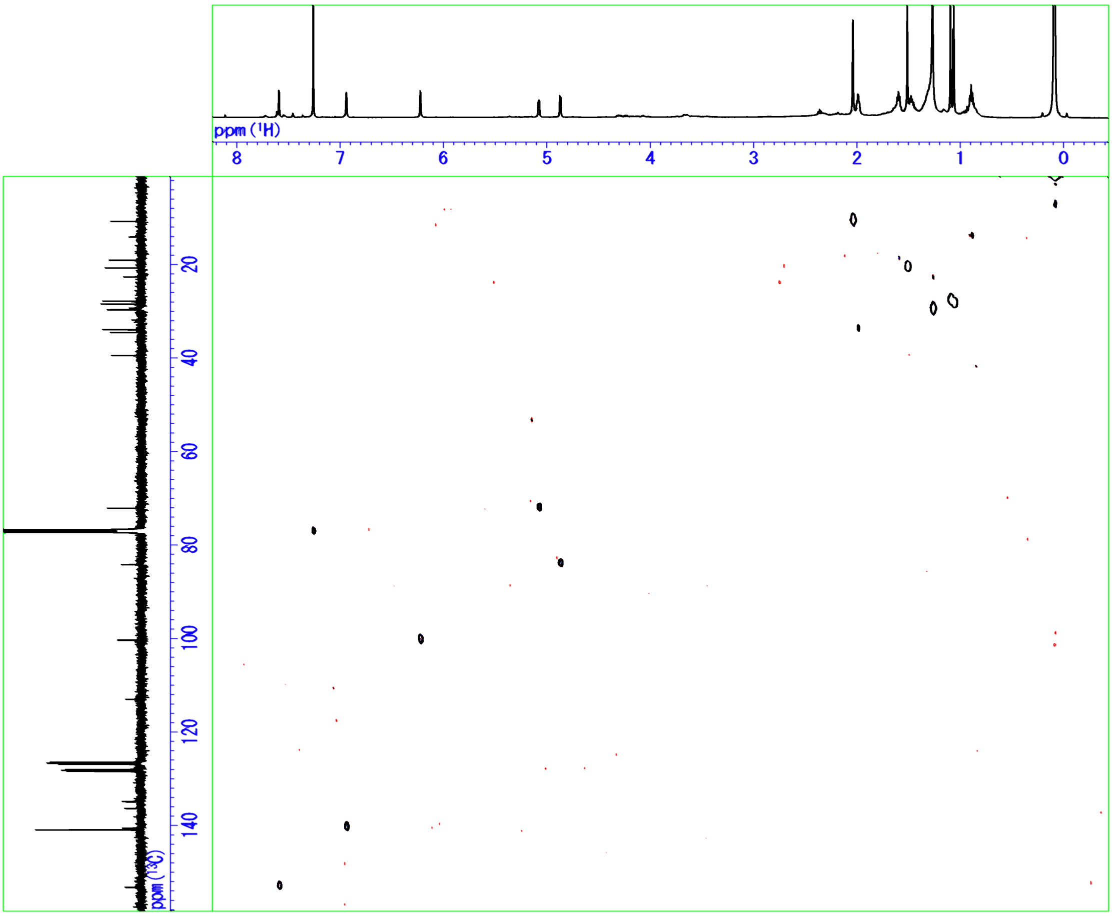


**Supplementary Data 2.5. Proton-detected heteronuclear multiple quantum coherence (HMQC) spectrum of BSB.**

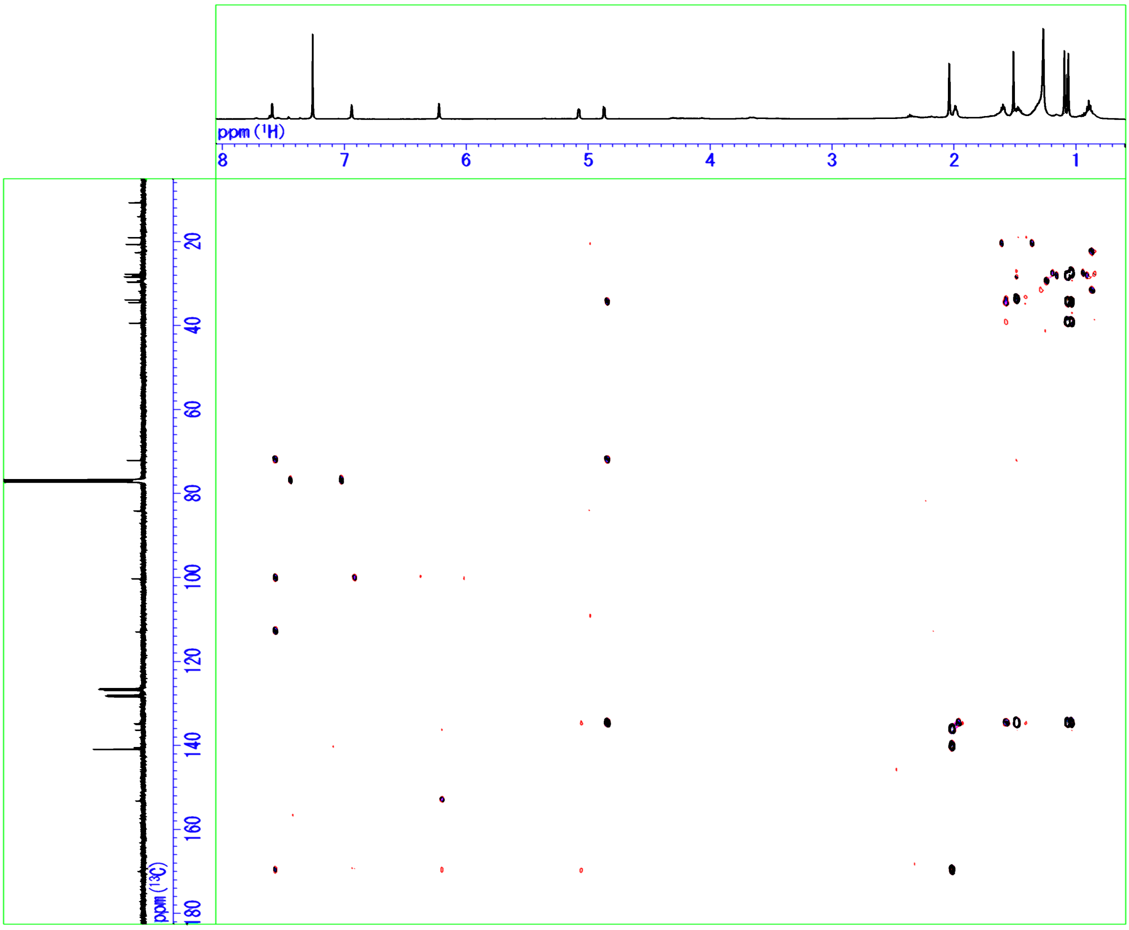


**Supplementary Data 2.6. Proton-detected multiple-bond heteronuclear multiple quantum coherence (HMBC) spectrum of BSB.**

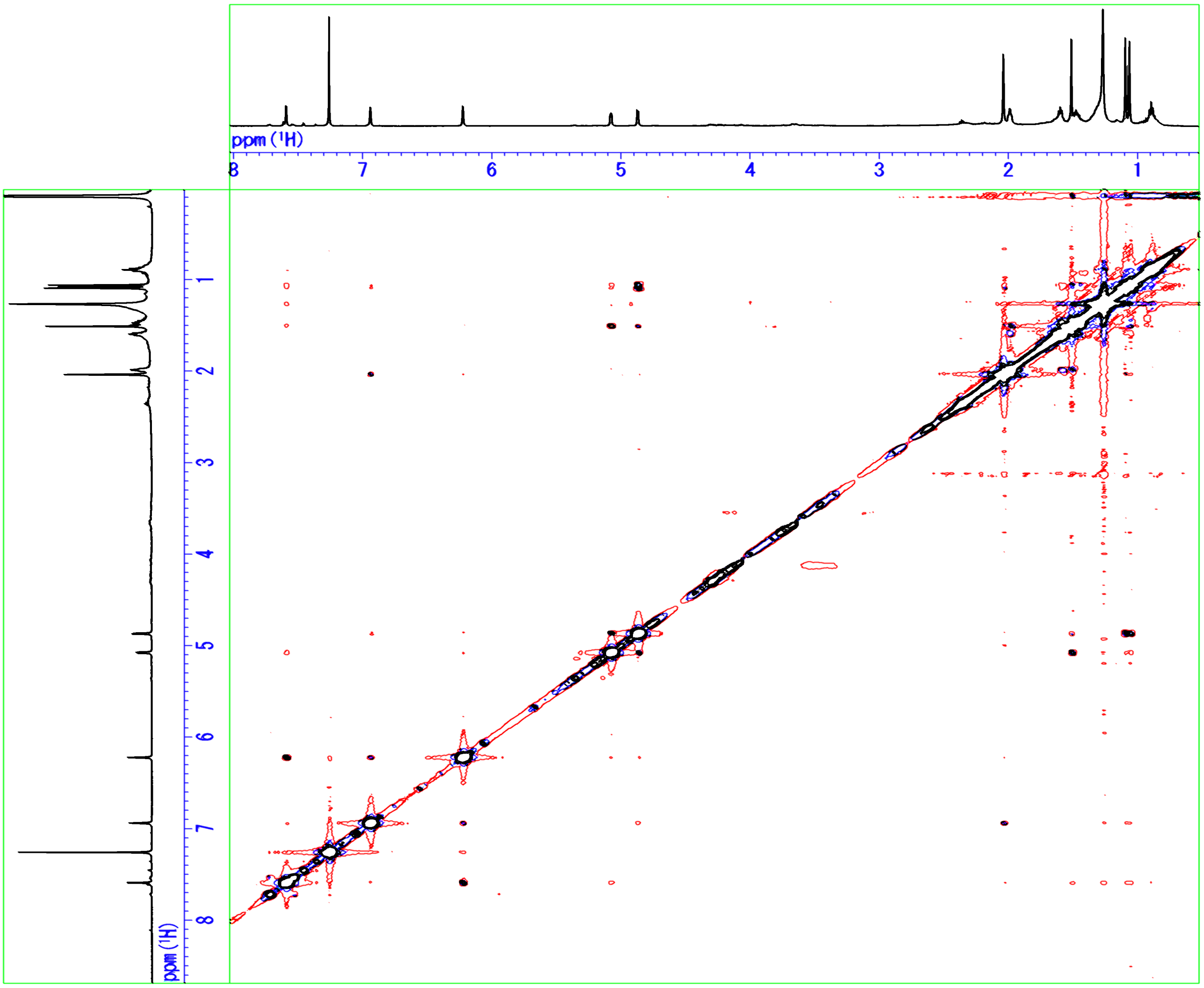


**Supplementary Data 2.7. Nuclear Overhauser effect spectroscopy (NOESY) spectrum of BSB.**

## Supplementary Data 2.8. The DP4+ evaluation of BSB.

| Settings | Type of data (shifts) |
|---|---|
| Default | Shielding tensors |

| Functional | Solvent? | Basis Set |
|---|---|---|
| mPW1PW91 | PCM | 6-31+G(d,p) |

| Isomer Nº | | | 1 | 2 | 3 | 4 |
|---|---|---|---|---|---|---|
| DP4+ (%) | | H data | 92.52% | 7.48% | 0.00% | 0.00% |
| | | C data | 65.17% | 34.83% | 0.00% | 0.00% |
| | | All data | **95.86%** | **4.14%** | **0.00%** | **0.00%** |
| **Type** | **sp2?** | **Exp** | **1** | **2** | **3** | **4** |
| C | | 34.55 | 156.3108978 | 156.438558 | 156.328998 | 156.805368 |
| C | | 39.48 | 155.5809703 | 155.054708 | 156.613535 | 156.096504 |
| C | | 19.1 | 173.95942 | 173.948654 | 174.237927 | 174.013583 |
| C | | 33.95 | 159.9688864 | 159.816741 | 159.707436 | 158.982399 |
| C | x | 134.97 | 57.69810288 | 57.2281354 | 53.1139625 | 52.5242657 |
| C | x | 134.88 | 60.07233071 | 60.1557728 | 66.7872983 | 67.5125507 |
| C | | 84.2 | 112.6654347 | 112.708072 | 113.876809 | 113.909306 |
| C | | 72.16 | 119.9966067 | 119.787695 | 126.992913 | 126.923748 |
| C | x | 113.01 | 83.31234758 | 83.3018902 | 85.9800907 | 85.9867258 |
| C | x | 153.25 | 42.97892008 | 42.2902127 | 42.0601688 | 40.6092141 |
| C | | 100.37 | 95.29158087 | 94.4867917 | 95.7594594 | 94.4003099 |
| C | x | 140.57 | 55.04424822 | 55.3686328 | 54.4831687 | 54.7750403 |
| C | x | 136.39 | 58.76501166 | 58.5500559 | 58.8949791 | 59.4653628 |
| C | x | 169.88 | 28.47600372 | 28.4463106 | 28.449233 | 28.3396349 |
| C | | 10.79 | 182.4727677 | 182.587334 | 182.431039 | 182.43763 |
| C | | 28.5 | 167.3713836 | 166.892672 | 167.789684 | 169.63716 |
| C | | 27.86 | 167.2167351 | 167.723391 | 170.128629 | 167.531117 |
| C | | 20.75 | 173.6375669 | 173.526385 | 172.22927 | 172.113813 |
| C | x | 170.05 | 29.64545722 | 30.055155 | 28.5526808 | 28.5377225 |
| | | | | | | |
| H | | 1.986 | 29.50799707 | 29.4459314 | 29.3938945 | 29.3234492 |
| H | | 1.991 | 29.45021513 | 29.4963062 | 29.2942072 | 29.4522749 |
| H | | 4.87 | 26.6383005 | 26.6312959 | 26.4833294 | 26.5085501 |
| H | | 5.08 | 26.2960439 | 26.2619315 | 26.5280445 | 26.5984684 |
| H | x | 7.59 | 23.75606252 | 23.7982787 | 23.7321319 | 23.6540421 |
| H | | 6.22 | 25.2335371 | 25.3859827 | 25.1899216 | 25.3814433 |
| H | x | 6.94 | 24.287918 | 24.2819167 | 24.2685488 | 24.2440737 |
| H | | 2.04 | 29.49285856 | 29.5064785 | 29.5049371 | 29.5234583 |
| H | | 1.06 | 30.57935022 | 30.4915981 | 30.5772771 | 30.427606 |
| H | | 1.1 | 30.48725972 | 30.5452405 | 30.4056317 | 30.6434665 |
| H | | 1.51 | 29.99820346 | 29.9763016 | 29.7700553 | 29.7411423 |

Experimental chemical shifts and Boltzmann-averaged shielding tensors calculated for (4*S**,5*R**,2'*R**)-, (4*R**,5*S**,2'*R**)-, (4*S**,5*S**,2'*R**)-, and (4*R**,5*R**,2'*R**)-isomers of BSB (Isomer Nº 1, 2, 3, and 4, respectively) were introduced to the main sheet of the Sarotti's DP4+ EXCEL file. The chemical shifts of methylene protons at H-9 and H-10 were not introduced since the exact chemical shifts were not assigned due to their multiplicity.

**Supplementary Data 2.9. Unscaled chemical shifts of DSB isomers calculated in the DP4+ spread sheet.**

| Type | Exp | 1 | 2 | 3 | 4 |
|---|---|---|---|---|---|
| C | 34.55 | 40.30 | 40.17 | 40.28 | 39.80 |
| C | 39.48 | 41.03 | 41.55 | 40.00 | 40.51 |
| C | 19.1 | 22.65 | 22.66 | 22.37 | 22.60 |
| C | 33.95 | 36.64 | 36.79 | 36.90 | 37.63 |
| C | 134.97 | 138.91 | 139.38 | 143.50 | 144.09 |
| C | 134.88 | 136.54 | 136.45 | 129.82 | 129.10 |
| C | 84.2 | 83.94 | 83.90 | 82.73 | 82.70 |
| C | 72.16 | 76.61 | 76.82 | 69.62 | 69.69 |
| C | 113.01 | 113.30 | 113.31 | 110.63 | 110.62 |
| C | 153.25 | 153.63 | 154.32 | 154.55 | 156.00 |
| C | 100.37 | 101.32 | 102.12 | 100.85 | 102.21 |
| C | 140.57 | 141.57 | 141.24 | 142.13 | 141.83 |
| C | 136.39 | 137.84 | 138.06 | 137.71 | 137.14 |
| C | 169.88 | 168.13 | 168.16 | 168.16 | 168.27 |
| C | 10.79 | 14.14 | 14.02 | 14.18 | 14.17 |
| C | 28.5 | 29.24 | 29.72 | 28.82 | 26.97 |
| C | 27.86 | 29.39 | 28.89 | 26.48 | 29.08 |
| C | 20.75 | 22.97 | 23.08 | 24.38 | 24.50 |
| C | 170.05 | 166.96 | 166.55 | 168.06 | 168.07 |
| - | - | - | - | - | - |
| H | 1.986 | 2.05 | 2.11 | 2.17 | 2.24 |
| H | 1.991 | 2.11 | 2.06 | 2.27 | 2.11 |
| H | 4.87 | 4.92 | 4.93 | 5.08 | 5.05 |
| H | 5.08 | 5.26 | 5.30 | 5.03 | 4.96 |
| H | 7.59 | 7.80 | 7.76 | 7.83 | 7.91 |
| H | 6.22 | 6.33 | 6.17 | 6.37 | 6.18 |
| H | 6.94 | 7.27 | 7.28 | 7.29 | 7.32 |
| H | 2.04 | 2.07 | 2.05 | 2.06 | 2.04 |
| H | 1.06 | 0.98 | 1.07 | 0.98 | 1.13 |
| H | 1.1 | 1.07 | 1.01 | 1.15 | 0.92 |
| H | 1.51 | 1.56 | 1.58 | 1.79 | 1.82 |

Isomers 1, 2, 3, and 4 are (4*S**,5*R**,2'*R**)-, (4*R**,5*S**,2'*R**)-, (4*S**,5*S**,2'*R**)-, and (4*R**,5*R**,2'*R**)-BSB, respectively.

**Supplementary Data 2.10. Scaled chemical shift of DSB isomers calculated in the DP4+ spread sheet.**

| Type | Exp | 1 | 2 | 3 | 4 |
|---|---|---|---|---|---|
| C | 34.55 | 37.7 | 37.4 | 38.6 | 37.9 |
| C | 39.48 | 38.4 | 38.8 | 38.3 | 38.6 |
| C | 19.1 | 19.6 | 19.5 | 20.4 | 20.3 |
| C | 33.95 | 33.9 | 34.0 | 35.1 | 35.7 |
| C | 134.97 | 138.5 | 138.8 | 143.7 | 144.1 |
| C | 134.88 | 136.0 | 135.8 | 129.7 | 128.8 |
| C | 84.2 | 82.3 | 82.1 | 81.8 | 81.6 |
| C | 72.16 | 74.8 | 74.9 | 68.4 | 68.3 |
| C | 113.01 | 112.3 | 112.2 | 110.2 | 110.0 |
| C | 153.25 | 153.5 | 154.1 | 154.9 | 156.2 |
| C | 100.37 | 100.0 | 100.7 | 100.2 | 101.4 |
| C | 140.57 | 141.2 | 140.7 | 142.3 | 141.8 |
| C | 136.39 | 137.4 | 137.5 | 137.8 | 137.0 |
| C | 169.88 | 168.3 | 168.2 | 168.8 | 168.7 |
| C | 10.79 | 10.9 | 10.7 | 12.0 | 11.8 |
| C | 28.5 | 26.3 | 26.7 | 26.9 | 24.8 |
| C | 27.86 | 26.5 | 25.9 | 24.5 | 27.0 |
| C | 20.75 | 19.9 | 20.0 | 22.4 | 22.3 |
| C | 170.05 | 167.1 | 166.6 | 168.7 | 168.5 |
| - | - | - | - | - | - |
| H | 1.986 | 2.0 | 2.1 | 2.1 | 2.2 |
| H | 1.991 | 2.1 | 2.0 | 2.2 | 2.0 |
| H | 4.87 | 4.8 | 4.8 | 4.9 | 4.9 |
| H | 5.08 | 5.1 | 5.2 | 4.9 | 4.8 |
| H | 7.59 | 7.6 | 7.6 | 7.6 | 7.7 |
| H | 6.22 | 6.1 | 6.0 | 6.2 | 6.0 |
| H | 6.94 | 7.1 | 7.1 | 7.1 | 7.1 |
| H | 2.04 | 2.0 | 2.0 | 1.9 | 2.0 |
| H | 1.06 | 1.0 | 1.1 | 0.9 | 1.1 |
| H | 1.1 | 1.1 | 1.0 | 1.1 | 0.9 |
| H | 1.51 | 1.5 | 1.6 | 1.7 | 1.7 |

Isomers 1, 2, 3, and 4 are (4*S**,5*R**,2'*R**)-, (4*R**,5*S**,2'*R**)-, (4*S**,5*S**,2'*R**)-, and (4*R**,5*R**,2'*R**)-BSB, respectively.

**Supplementary Data 2.11. Unscaled errors chemical shift of DSB isomers calculated in the DP4+ spread sheet.**

| Type | Exp | 1 | 2 | 3 | 4 |
|---|---|---|---|---|---|
| C | 34.55 | 5.75 | 5.62 | 5.73 | 5.25 |
| C | 39.48 | 1.55 | 2.07 | 0.52 | 1.03 |
| C | 19.1 | 3.55 | 3.56 | 3.27 | 3.50 |
| C | 33.95 | 2.69 | 2.84 | 2.95 | 3.68 |
| C | 134.97 | 3.94 | 4.41 | 8.53 | 9.12 |
| C | 134.88 | 1.66 | 1.57 | -5.06 | -5.78 |
| C | 84.2 | -0.26 | -0.30 | -1.47 | -1.50 |
| C | 72.16 | 4.45 | 4.66 | -2.54 | -2.47 |
| C | 113.01 | 0.29 | 0.30 | -2.38 | -2.39 |
| C | 153.25 | 0.38 | 1.07 | 1.30 | 2.75 |
| C | 100.37 | 0.95 | 1.75 | 0.48 | 1.84 |
| C | 140.57 | 1.00 | 0.67 | 1.56 | 1.26 |
| C | 136.39 | 1.45 | 1.67 | 1.32 | 0.75 |
| C | 169.88 | -1.75 | -1.72 | -1.72 | -1.61 |
| C | 10.79 | 3.35 | 3.23 | 3.39 | 3.38 |
| C | 28.5 | 0.74 | 1.22 | 0.32 | -1.53 |
| C | 27.86 | 1.53 | 1.03 | -1.38 | 1.22 |
| C | 20.75 | 2.22 | 2.33 | 3.63 | 3.75 |
| C | 170.05 | -3.09 | -3.50 | -1.99 | -1.98 |
| - | - | - | - | - | - |
| H | 1.986 | 0.07 | 0.13 | 0.18 | 0.25 |
| H | 1.991 | 0.12 | 0.07 | 0.27 | 0.12 |
| H | 4.87 | 0.05 | 0.06 | 0.21 | 0.18 |
| H | 5.08 | 0.18 | 0.22 | -0.05 | -0.12 |
| H | 7.59 | 0.21 | 0.17 | 0.24 | 0.32 |
| H | 6.22 | 0.11 | -0.05 | 0.15 | -0.04 |
| H | 6.94 | 0.33 | 0.34 | 0.35 | 0.38 |
| H | 2.04 | 0.03 | 0.01 | 0.02 | 0.00 |
| H | 1.06 | -0.08 | 0.01 | -0.08 | 0.07 |
| H | 1.1 | -0.03 | -0.09 | 0.05 | -0.18 |
| H | 1.51 | 0.05 | 0.07 | 0.28 | 0.31 |

Isomers 1, 2, 3, and 4 are (4*S**,5*R**,2'*R**)-, (4*R**,5*S**,2'*R**)-, (4*S**,5*S**,2'*R**)-, and (4*R**,5*R**,2'*R**)-BSB, respectively. MAEs (mean absolute errors) in the $^{13}C$ and $^{1}H$ NMR chemical shifts of isomers 1, 2, 3, and 4 are 2.14 and 0.11, 2.29 and 0.11, 2.60 and 0.16, and 2.92 and 0.18, respectively. RMSs (root mean squares) in the $^{13}C$ and $^{1}H$ NMR chemical shifts of isomers 1, 2, 3, and 4 are 2.61 and 0.15, 2.73 and 0.15, 3.25 and 0.16, and 3.56 and 0.22, respectively.

**Supplementary Data 2.12. Scaled errors chemical shift of DSB isomers calculated in the DP4+ spread sheet.**

| Type | Exp | 1 | 2 | 3 | 4 |
|---|---|---|---|---|---|
| C | 34.55 | 3.11 | 2.87 | 4.04 | 3.32 |
| C | 39.48 | -1.08 | -0.64 | -1.18 | -0.88 |
| C | 19.1 | 0.51 | 0.43 | 1.26 | 1.25 |
| C | 33.95 | -0.03 | 0.02 | 1.20 | 1.71 |
| C | 134.97 | 3.49 | 3.85 | 8.69 | 9.11 |
| C | 134.88 | 1.16 | 0.95 | -5.14 | -6.07 |
| C | 84.2 | -1.93 | -2.08 | -2.40 | -2.64 |
| C | 72.16 | 2.62 | 2.72 | -3.71 | -3.85 |
| C | 113.01 | -0.73 | -0.84 | -2.81 | -3.01 |
| C | 153.25 | 0.26 | 0.84 | 1.66 | 2.96 |
| C | 100.37 | -0.34 | 0.37 | -0.13 | 1.06 |
| C | 140.57 | 0.61 | 0.15 | 1.69 | 1.22 |
| C | 136.39 | 0.98 | 1.08 | 1.38 | 0.62 |
| C | 169.88 | -1.54 | -1.64 | -1.12 | -1.17 |
| C | 10.79 | 0.12 | -0.09 | 1.23 | 0.98 |
| C | 28.5 | -2.15 | -1.76 | -1.58 | -3.69 |
| C | 27.86 | -1.35 | -1.97 | -3.32 | -0.91 |
| C | 20.75 | -0.81 | -0.79 | 1.65 | 1.53 |
| C | 170.05 | -2.91 | -3.46 | -1.39 | -1.54 |
| - | - | - | - | - | - |
| H | 1.986 | 0.03 | 0.09 | 0.07 | 0.17 |
| H | 1.991 | 0.08 | 0.03 | 0.16 | 0.04 |
| H | 4.87 | -0.08 | -0.06 | 0.03 | 0.04 |
| H | 5.08 | 0.04 | 0.09 | -0.22 | -0.26 |
| H | 7.59 | -0.03 | -0.02 | 0.01 | 0.11 |
| H | 6.22 | -0.08 | -0.20 | -0.05 | -0.21 |
| H | 6.94 | 0.11 | 0.16 | 0.13 | 0.18 |
| H | 2.04 | -0.01 | -0.03 | -0.10 | -0.08 |
| H | 1.06 | -0.07 | -0.01 | -0.17 | 0.02 |
| H | 1.1 | -0.03 | -0.10 | -0.04 | -0.24 |
| H | 1.51 | 0.04 | 0.05 | 0.17 | 0.24 |

Isomers 1, 2, 3, and 4 are (4*S**,5*R**,2'*R**)-, (4*R**,5*S**,2'*R**)-, (4*S**,5*S**,2'*R**)-, and (4*R**,5*R**,2'*R**)-BSB, respectively. CMAEs (corrected mean absolute errors) in the $^{13}$C and $^{1}$H NMR chemical shifts of isomers 1, 2, 3, and 4 are 1.35 and 0.05, 1.40 and 0.07, 2.34 and 0.12, and 2.55 and 0.15, respectively. CRMSs (corrected root mean squares) ) in the $^{13}$C and $^{1}$H NMR chemical shifts of isomers 1, 2, 3, and 4 are 1.70 and 0.06, 1.80 and 0.09, 3.00 and 0.13, and 3.31 and 0.17, respectively.

**Supplementary Data 2.13. Energy analysis and Boltzmann weights of low-energy conformers of 4*S**,5*R**,2'*R** isomer optimized in the gas phase at the B3LYP/6-31G(d) level for DP4+ analysis.**

| Conformer | Total energy (au) | ΔE (kJ/mol) | Gibbs free energy (au) | ΔG (kJ/mol) | Boltzmann weights |
|---|---|---|---|---|---|
| 1a | -1189.782238 | 0.00 | -1189.431809 | 0.00 | 0.300 |
| 1b | -1189.782081 | 0.41 | -1189.431688 | 0.32 | 0.264 |
| 1c | -1189.781414 | 2.16 | -1189.431216 | 1.56 | 0.160 |
| 1d | -1189.781100 | 2.99 | -1189.430360 | 3.80 | 0.065 |
| 1e | -1189.781253 | 2.59 | -1189.430261 | 4.06 | 0.058 |
| 1f | -1189.783336 | -2.88 | -1189.430172 | 4.30 | 0.053 |
| 1g | -1189.780315 | 5.05 | -1189.429831 | 5.19 | 0.037 |
| 1h | -1189.782805 | -1.49 | -1189.429588 | 5.83 | 0.029 |
| 1i | -1189.782397 | -0.42 | -1189.429540 | 5.96 | 0.027 |
| 1j | -1189.781265 | 2.55 | -1189.428196 | 9.49 | 0.007 |

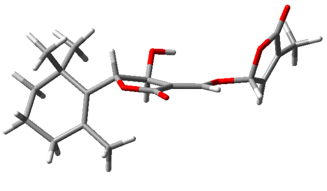

1a (30.0%)

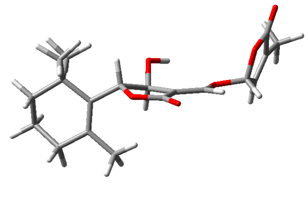

1b (26.4%)

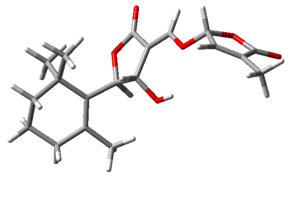

1c (16.0%)

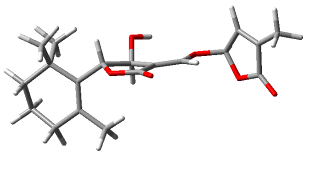

1d (6.5%)

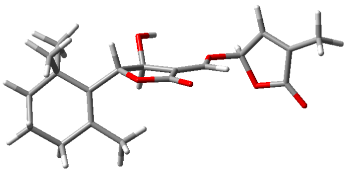

1e (5.8%)

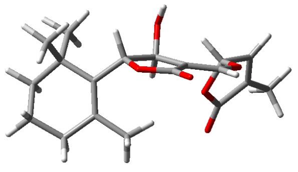

1f (5.3%)

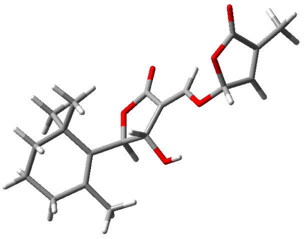

1g (3.7%)

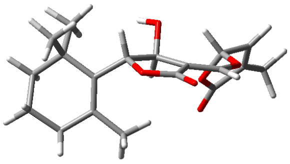

1h (2.9%)

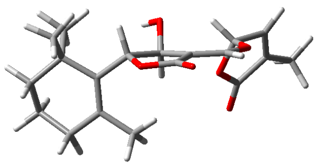

1i (2.7%)

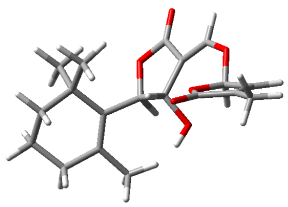

1j (0.7%)

**Supplementary Data 2.14. Low-energy conformers of 4*S**,5*R**,2'*R** isomer optimized in the gas phase at the B3LYP/6-31G(d) level for DP4+ analysis.**

**Supplementary Data 2.15. Cartesian coordinates of the conformers 1a−1j of 4*S**,5*R**,2'*R** isomer.**

Conformer 1a

| Center Number | Atomic Number | Atomic Type | Coordinates (Angstroms) | | |
|---|---|---|---|---|---|
| | | | X | Y | Z |
| 1 | 6 | 0 | -4.93643 | -1.778642 | 0.294529 |
| 2 | 6 | 0 | -5.70022 | -0.618048 | 0.92642 |
| 3 | 6 | 0 | -4.74679 | 0.195454 | 1.799781 |
| 4 | 6 | 0 | -3.41482 | 0.490224 | 1.136125 |
| 5 | 6 | 0 | -2.98895 | -0.163409 | 0.035084 |
| 6 | 6 | 0 | -3.77945 | -1.313016 | -0.62213 |
| 7 | 6 | 0 | -2.87094 | -2.543351 | -0.86326 |
| 8 | 6 | 0 | -4.3531 | -0.843121 | -1.98226 |
| 9 | 6 | 0 | -2.66718 | 1.610168 | 1.827006 |
| 10 | 6 | 0 | -1.70951 | 0.23145 | -0.68487 |
| 11 | 6 | 0 | -0.34974 | -0.084309 | 0.00997 |
| 12 | 6 | 0 | 0.424478 | 1.183277 | -0.22882 |
| 13 | 6 | 0 | -0.46385 | 2.211121 | -0.81333 |
| 14 | 8 | 0 | -1.69928 | 1.662147 | -0.98135 |
| 15 | 8 | 0 | 0.204499 | -1.239029 | -0.61131 |
| 16 | 8 | 0 | -0.21107 | 3.358924 | -1.09747 |
| 17 | 6 | 0 | 1.722822 | 1.412557 | -0.01615 |
| 18 | 8 | 0 | 2.517447 | 0.448411 | 0.536187 |
| 19 | 6 | 0 | 3.902884 | 0.735387 | 0.601873 |
| 20 | 6 | 0 | 4.582429 | -0.271923 | 1.487379 |
| 21 | 6 | 0 | 5.49871 | -0.940184 | 0.782149 |
| 22 | 6 | 0 | 5.466141 | -0.408454 | -0.61672 |
| 23 | 8 | 0 | 4.50798 | 0.587901 | -0.67665 |
| 24 | 8 | 0 | 6.130795 | -0.730638 | -1.56308 |
| 25 | 6 | 0 | 6.437868 | -2.034304 | 1.164498 |
| 26 | 1 | 0 | -4.52292 | -2.403712 | 1.099174 |
| 27 | 1 | 0 | -5.60869 | -2.42404 | -0.2855 |
| 28 | 1 | 0 | -6.54261 | -0.987949 | 1.523747 |
| 29 | 1 | 0 | -6.12752 | 0.02549 | 0.146898 |
| 30 | 1 | 0 | -4.55764 | -0.336572 | 2.746768 |
| 31 | 1 | 0 | -5.21046 | 1.148492 | 2.092384 |
| 32 | 1 | 0 | -2.06149 | -2.350635 | -1.57264 |
| 33 | 1 | 0 | -2.41174 | -2.882653 | 0.072071 |
| 34 | 1 | 0 | -3.47296 | -3.368686 | -1.26367 |
| 35 | 1 | 0 | -3.56752 | -0.487932 | -2.65807 |
| 36 | 1 | 0 | -4.86648 | -1.672844 | -2.48408 |
| 37 | 1 | 0 | -5.06826 | -0.023801 | -1.85642 |
| 38 | 1 | 0 | -2.80148 | 1.529605 | 2.913593 |
| 39 | 1 | 0 | -1.59499 | 1.631047 | 1.630042 |
| 40 | 1 | 0 | -3.07161 | 2.586111 | 1.527624 |
| 41 | 1 | 0 | -1.66121 | -0.268285 | -1.65436 |
| 42 | 1 | 0 | -0.49484 | -0.272226 | 1.082977 |
| 43 | 1 | 0 | 1.100706 | -1.34883 | -0.25422 |
| 44 | 1 | 0 | 2.193584 | 2.359293 | -0.27328 |
| 45 | 1 | 0 | 4.040144 | 1.776861 | 0.918318 |
| 46 | 1 | 0 | 4.315374 | -0.394773 | 2.530473 |
| 47 | 1 | 0 | 7.472624 | -1.73868 | 0.956634 |
| 48 | 1 | 0 | 6.347368 | -2.291435 | 2.2229 |
| 49 | 1 | 0 | 6.24352 | -2.930856 | 0.564524 |

Conformer 1b

| Center Number | Atomic Number | Atomic Type | Coordinates (Angstroms) | | |
|---|---|---|---|---|---|
| | | | X | Y | Z |
| 1 | 6 | 0 | -5.26931 | -1.219936 | -0.34076 |
| 2 | 6 | 0 | -5.23148 | -1.537616 | 1.151467 |
| 3 | 6 | 0 | -4.64324 | -0.344391 | 1.90163 |
| 4 | 6 | 0 | -3.37958 | 0.205759 | 1.268202 |
| 5 | 6 | 0 | -3.0119 | -0.088838 | 0.003396 |
| 6 | 6 | 0 | -3.86619 | -0.960808 | -0.94119 |
| 7 | 6 | 0 | -3.15159 | -2.310624 | -1.19805 |
| 8 | 6 | 0 | -4.08732 | -0.244344 | -2.29723 |
| 9 | 6 | 0 | -2.61868 | 1.113018 | 2.208677 |
| 10 | 6 | 0 | -1.70912 | 0.412764 | -0.59837 |
| 11 | 6 | 0 | -0.37497 | 0.005617 | 0.089682 |
| 12 | 6 | 0 | 0.433874 | 1.267774 | -0.04289 |
| 13 | 6 | 0 | -0.43882 | 2.373498 | -0.49537 |
| 14 | 8 | 0 | -1.6923 | 1.872793 | -0.69532 |
| 15 | 8 | 0 | 0.148783 | -1.115549 | -0.61354 |
| 16 | 8 | 0 | -0.16446 | 3.539817 | -0.65627 |
| 17 | 6 | 0 | 1.742475 | 1.439827 | 0.159399 |
| 18 | 8 | 0 | 2.520322 | 0.405117 | 0.597059 |
| 19 | 6 | 0 | 3.914953 | 0.645305 | 0.650861 |
| 20 | 6 | 0 | 4.585837 | -0.46272 | 1.414233 |
| 21 | 6 | 0 | 5.463567 | -1.083185 | 0.621694 |
| 22 | 6 | 0 | 5.411753 | -0.41559 | -0.71709 |
| 23 | 8 | 0 | 4.482909 | 0.607716 | -0.65251 |
| 24 | 8 | 0 | 6.042544 | -0.661151 | -1.70857 |
| 25 | 6 | 0 | 6.378926 | -2.234821 | 0.868952 |
| 26 | 1 | 0 | -5.74957 | -2.030337 | -0.90427 |
| 27 | 1 | 0 | -5.89113 | -0.325484 | -0.49003 |
| 28 | 1 | 0 | -4.61915 | -2.429906 | 1.335723 |
| 29 | 1 | 0 | -6.23754 | -1.764753 | 1.524821 |
| 30 | 1 | 0 | -4.42581 | -0.61289 | 2.945557 |
| 31 | 1 | 0 | -5.38828 | 0.465754 | 1.960605 |
| 32 | 1 | 0 | -2.12285 | -2.171607 | -1.54626 |
| 33 | 1 | 0 | -3.10254 | -2.91475 | -0.28541 |
| 34 | 1 | 0 | -3.69181 | -2.887672 | -1.95929 |
| 35 | 1 | 0 | -3.1759 | -0.172 | -2.89919 |
| 36 | 1 | 0 | -4.81853 | -0.806007 | -2.89134 |
| 37 | 1 | 0 | -4.47594 | 0.769434 | -2.14986 |
| 38 | 1 | 0 | -2.2102 | 0.541619 | 3.054563 |
| 39 | 1 | 0 | -1.80784 | 1.669351 | 1.742169 |
| 40 | 1 | 0 | -3.30694 | 1.851326 | 2.642187 |
| 41 | 1 | 0 | -1.61187 | 0.047014 | -1.62182 |
| 42 | 1 | 0 | -0.53543 | -0.257397 | 1.143822 |
| 43 | 1 | 0 | 1.037249 | -1.283051 | -0.25984 |
| 44 | 1 | 0 | 2.235588 | 2.394037 | -0.01473 |
| 45 | 1 | 0 | 4.092076 | 1.646369 | 1.06343 |
| 46 | 1 | 0 | 4.341744 | -0.68021 | 2.447477 |
| 47 | 1 | 0 | 7.416835 | -1.947598 | 0.664981 |
| 48 | 1 | 0 | 6.305391 | -2.594135 | 1.898523 |
| 49 | 1 | 0 | 6.144846 | -3.061186 | 0.187894 |

**Supplementary Data 2.15. Cartesian coordinates of the conformers 1a−1j of 4*S**,5*R**,2'*R** isomer (continued).**

Conformer 1c

| Center Number | Atomic Number | Atomic Type | Coordinates (Angstroms) X | Y | Z |
|---|---|---|---|---|---|
| 1 | 6 | 0 | -4.72572 | -0.480915 | 1.56698 |
| 2 | 6 | 0 | -5.58995 | -1.321076 | 0.631046 |
| 3 | 6 | 0 | -4.6935 | -2.237392 | -0.19916 |
| 4 | 6 | 0 | -3.42352 | -1.582746 | -0.7075 |
| 5 | 6 | 0 | -2.97762 | -0.385201 | -0.2657 |
| 6 | 6 | 0 | -3.72829 | 0.432423 | 0.811132 |
| 7 | 6 | 0 | -4.50757 | 1.604207 | 0.157356 |
| 8 | 6 | 0 | -2.77458 | 1.017539 | 1.878663 |
| 9 | 6 | 0 | -2.72584 | -2.431547 | -1.75666 |
| 10 | 6 | 0 | -1.68925 | 0.161123 | -0.86919 |
| 11 | 6 | 0 | -0.35998 | -0.158687 | -0.1166 |
| 12 | 6 | 0 | 0.385374 | 1.142339 | -0.22571 |
| 13 | 6 | 0 | -0.51539 | 2.186914 | -0.75295 |
| 14 | 8 | 0 | -1.72377 | 1.610945 | -1.02016 |
| 15 | 8 | 0 | 0.266737 | -1.254425 | -0.77574 |
| 16 | 8 | 0 | -0.30067 | 3.363535 | -0.9263 |
| 17 | 6 | 0 | 1.66739 | 1.390096 | 0.053831 |
| 18 | 8 | 0 | 2.470302 | 0.406432 | 0.557863 |
| 19 | 6 | 0 | 3.844257 | 0.722745 | 0.694485 |
| 20 | 6 | 0 | 4.522395 | -0.334215 | 1.521188 |
| 21 | 6 | 0 | 5.479729 | -0.920691 | 0.797938 |
| 22 | 6 | 0 | 5.478078 | -0.28029 | -0.55505 |
| 23 | 8 | 0 | 4.49493 | 0.693252 | -0.56962 |
| 24 | 8 | 0 | 6.1821 | -0.50906 | -1.5001 |
| 25 | 6 | 0 | 6.436383 | -2.017392 | 1.125584 |
| 26 | 1 | 0 | -5.35021 | 0.145877 | 2.216819 |
| 27 | 1 | 0 | -4.16238 | -1.155546 | 2.22849 |
| 28 | 1 | 0 | -6.17334 | -0.668768 | -0.02992 |
| 29 | 1 | 0 | -6.31511 | -1.914395 | 1.201577 |
| 30 | 1 | 0 | -5.24512 | -2.64229 | -1.06026 |
| 31 | 1 | 0 | -4.40576 | -3.120098 | 0.395348 |
| 32 | 1 | 0 | -3.83352 | 2.302535 | -0.34081 |
| 33 | 1 | 0 | -5.22204 | 1.242383 | -0.58915 |
| 34 | 1 | 0 | -5.06862 | 2.153196 | 0.924953 |
| 35 | 1 | 0 | -2.07533 | 1.748697 | 1.46644 |
| 36 | 1 | 0 | -3.3639 | 1.533837 | 2.646303 |
| 37 | 1 | 0 | -2.2031 | 0.228854 | 2.382888 |
| 38 | 1 | 0 | -3.11166 | -2.204211 | -2.76149 |
| 39 | 1 | 0 | -1.64102 | -2.322498 | -1.76977 |
| 40 | 1 | 0 | -2.94291 | -3.491235 | -1.57585 |
| 41 | 1 | 0 | -1.55978 | -0.231358 | -1.87958 |
| 42 | 1 | 0 | -0.55787 | -0.418036 | 0.931994 |
| 43 | 1 | 0 | 1.141212 | -1.367493 | -0.36943 |
| 44 | 1 | 0 | 2.118416 | 2.36708 | -0.10748 |
| 45 | 1 | 0 | 3.941208 | 1.738824 | 1.096748 |
| 46 | 1 | 0 | 4.225037 | -0.546187 | 2.541537 |
| 47 | 1 | 0 | 7.468578 | -1.680072 | 0.976941 |
| 48 | 1 | 0 | 6.319205 | -2.359526 | 2.156954 |
| 49 | 1 | 0 | 6.286613 | -2.868401 | 0.451108 |

Conformer 1d

| Center Number | Atomic Number | Atomic Type | Coordinates (Angstroms) X | Y | Z |
|---|---|---|---|---|---|
| 1 | 6 | 0 | 5.570029 | -0.111612 | -0.10143 |
| 2 | 6 | 0 | 5.500702 | 1.140616 | -0.97101 |
| 3 | 6 | 0 | 4.473506 | 2.103887 | -0.3799 |
| 4 | 6 | 0 | 3.164407 | 1.436957 | -0.0026 |
| 5 | 6 | 0 | 3.040915 | 0.099306 | 0.128871 |
| 6 | 6 | 0 | 4.225958 | -0.876406 | -0.0321 |
| 7 | 6 | 0 | 4.03935 | -1.719692 | -1.31769 |
| 8 | 6 | 0 | 4.318847 | -1.831183 | 1.184848 |
| 9 | 6 | 0 | 2.054651 | 2.440614 | 0.214389 |
| 10 | 6 | 0 | 1.708909 | -0.563324 | 0.442207 |
| 11 | 6 | 0 | 0.530571 | -0.352904 | -0.55012 |
| 12 | 6 | 0 | -0.6367 | -0.192562 | 0.385329 |
| 13 | 6 | 0 | -0.14771 | -0.067384 | 1.775475 |
| 14 | 8 | 0 | 1.212985 | -0.170598 | 1.761305 |
| 15 | 8 | 0 | 0.469202 | -1.502716 | -1.39087 |
| 16 | 8 | 0 | -0.78063 | 0.112439 | 2.790118 |
| 17 | 6 | 0 | -1.94174 | -0.184746 | 0.099444 |
| 18 | 8 | 0 | -2.36907 | -0.342108 | -1.18714 |
| 19 | 6 | 0 | -3.76431 | -0.256866 | -1.43582 |
| 20 | 6 | 0 | -4.62169 | -1.194068 | -0.62248 |
| 21 | 6 | 0 | -5.55648 | -0.497883 | 0.031553 |
| 22 | 6 | 0 | -5.34862 | 0.947554 | -0.29717 |
| 23 | 8 | 0 | -4.25654 | 1.04594 | -1.14239 |
| 24 | 8 | 0 | -5.98003 | 1.903308 | 0.062213 |
| 25 | 6 | 0 | -6.65181 | -0.92644 | 0.949169 |
| 26 | 1 | 0 | 6.348134 | -0.797976 | -0.45991 |
| 27 | 1 | 0 | 5.862385 | 0.187095 | 0.91557 |
| 28 | 1 | 0 | 5.213939 | 0.87669 | -1.99725 |
| 29 | 1 | 0 | 6.483829 | 1.622912 | -1.03433 |
| 30 | 1 | 0 | 4.261762 | 2.922478 | -1.08276 |
| 31 | 1 | 0 | 4.889715 | 2.592538 | 0.516099 |
| 32 | 1 | 0 | 3.061386 | -2.211344 | -1.34816 |
| 33 | 1 | 0 | 4.113512 | -1.096825 | -2.21591 |
| 34 | 1 | 0 | 4.811672 | -2.497048 | -1.37831 |
| 35 | 1 | 0 | 3.498094 | -2.554353 | 1.227636 |
| 36 | 1 | 0 | 5.249996 | -2.407963 | 1.125984 |
| 37 | 1 | 0 | 4.324612 | -1.271337 | 2.126647 |
| 38 | 1 | 0 | 1.787182 | 2.93485 | -0.73047 |
| 39 | 1 | 0 | 1.14997 | 2.027026 | 0.655909 |
| 40 | 1 | 0 | 2.40561 | 3.235373 | 0.886536 |
| 41 | 1 | 0 | 1.839688 | -1.645904 | 0.486854 |
| 42 | 1 | 0 | 0.678506 | 0.546272 | -1.16181 |
| 43 | 1 | 0 | -0.27832 | -1.369521 | -1.99522 |
| 44 | 1 | 0 | -2.69514 | -0.072354 | 0.873568 |
| 45 | 1 | 0 | -3.8435 | -0.422585 | -2.51377 |
| 46 | 1 | 0 | -4.4612 | -2.266374 | -0.62139 |
| 47 | 1 | 0 | -6.6855 | -2.013533 | 1.055803 |
| 48 | 1 | 0 | -6.51621 | -0.478191 | 1.940249 |
| 49 | 1 | 0 | -7.62018 | -0.573237 | 0.576673 |

**Supplementary Data 2.15. Cartesian coordinates of the conformers 1a−1j of 4*S**,5*R**,2'*R** isomer (continued).**

Conformer 1e

| Center Number | Atomic Number | Atomic Type | Coordinates (Angstroms) X | Y | Z |
|---|---|---|---|---|---|
| 1 | 6 | 0 | 5.385101 | -0.346993 | -0.80527 |
| 2 | 6 | 0 | 5.656218 | 1.132747 | -0.54744 |
| 3 | 6 | 0 | 4.428591 | 1.946478 | -0.95257 |
| 4 | 6 | 0 | 3.119873 | 1.361816 | -0.45584 |
| 5 | 6 | 0 | 3.015989 | 0.098541 | 0.007117 |
| 6 | 6 | 0 | 4.194855 | -0.895814 | 0.018256 |
| 7 | 6 | 0 | 3.786756 | -2.244965 | -0.62233 |
| 8 | 6 | 0 | 4.650131 | -1.15375 | 1.47638 |
| 9 | 6 | 0 | 1.988139 | 2.365095 | -0.50753 |
| 10 | 6 | 0 | 1.725605 | -0.42621 | 0.616621 |
| 11 | 6 | 0 | 0.519523 | -0.689749 | -0.33509 |
| 12 | 6 | 0 | -0.62653 | -0.094271 | 0.435388 |
| 13 | 6 | 0 | -0.11531 | 0.607045 | 1.63226 |
| 14 | 8 | 0 | 1.240048 | 0.471349 | 1.66191 |
| 15 | 8 | 0 | 0.424051 | -2.097491 | -0.54032 |
| 16 | 8 | 0 | -0.73113 | 1.222454 | 2.471948 |
| 17 | 6 | 0 | -1.93492 | -0.171101 | 0.175583 |
| 18 | 8 | 0 | -2.38485 | -0.867015 | -0.90854 |
| 19 | 6 | 0 | -3.77986 | -0.849513 | -1.17381 |
| 20 | 6 | 0 | -4.65752 | -1.298305 | -0.03219 |
| 21 | 6 | 0 | -5.55864 | -0.350686 | 0.243959 |
| 22 | 6 | 0 | -5.30566 | 0.791828 | -0.68941 |
| 23 | 8 | 0 | -4.22294 | 0.465686 | -1.4884 |
| 24 | 8 | 0 | -5.89882 | 1.830527 | -0.79175 |
| 25 | 6 | 0 | -6.65527 | -0.288691 | 1.253396 |
| 26 | 1 | 0 | 5.168985 | -0.48063 | -1.87511 |
| 27 | 1 | 0 | 6.273655 | -0.954545 | -0.58995 |
| 28 | 1 | 0 | 6.537981 | 1.46834 | -1.10698 |
| 29 | 1 | 0 | 5.876583 | 1.299852 | 0.514619 |
| 30 | 1 | 0 | 4.380584 | 2.033774 | -2.05053 |
| 31 | 1 | 0 | 4.511923 | 2.97924 | -0.58485 |
| 32 | 1 | 0 | 3.002106 | -2.766966 | -0.0678 |
| 33 | 1 | 0 | 3.422383 | -2.099504 | -1.64557 |
| 34 | 1 | 0 | 4.659938 | -2.908066 | -0.66532 |
| 35 | 1 | 0 | 3.8324 | -1.52843 | 2.101743 |
| 36 | 1 | 0 | 5.449089 | -1.905517 | 1.496777 |
| 37 | 1 | 0 | 5.027197 | -0.240468 | 1.947852 |
| 38 | 1 | 0 | 2.078187 | 2.978276 | -1.41347 |
| 39 | 1 | 0 | 0.989925 | 1.92647 | -0.50827 |
| 40 | 1 | 0 | 2.041378 | 3.05136 | 0.347956 |
| 41 | 1 | 0 | 1.914221 | -1.378998 | 1.115476 |
| 42 | 1 | 0 | 0.663199 | -0.183346 | -1.299 |
| 43 | 1 | 0 | -0.34535 | -2.244321 | -1.11348 |
| 44 | 1 | 0 | -2.67334 | 0.294573 | 0.821695 |
| 45 | 1 | 0 | -3.87879 | -1.47189 | -2.06741 |
| 46 | 1 | 0 | -4.53407 | -2.264784 | 0.443499 |
| 47 | 1 | 0 | -7.61611 | -0.101922 | 0.760274 |
| 48 | 1 | 0 | -6.7243 | -1.21427 | 1.830291 |
| 49 | 1 | 0 | -6.49112 | 0.546682 | 1.94386 |

Conformer 1f

| Center Number | Atomic Number | Atomic Type | Coordinates (Angstroms) X | Y | Z |
|---|---|---|---|---|---|
| 1 | 6 | 0 | 4.097777 | -2.061658 | 0.478111 |
| 2 | 6 | 0 | 4.557019 | -1.906857 | -0.9692 |
| 3 | 6 | 0 | 3.341168 | -1.643679 | -1.85538 |
| 4 | 6 | 0 | 2.391265 | -0.60388 | -1.29268 |
| 5 | 6 | 0 | 2.44372 | -0.190848 | -0.00926 |
| 6 | 6 | 0 | 3.411467 | -0.789363 | 1.032233 |
| 7 | 6 | 0 | 2.64772 | -1.21153 | 2.311555 |
| 8 | 6 | 0 | 4.49136 | 0.253561 | 1.413885 |
| 9 | 6 | 0 | 1.430113 | -0.076424 | -2.33533 |
| 10 | 6 | 0 | 1.574204 | 0.947742 | 0.504163 |
| 11 | 6 | 0 | 0.039157 | 0.677994 | 0.670656 |
| 12 | 6 | 0 | -0.58039 | 1.834986 | -0.06487 |
| 13 | 6 | 0 | 0.497242 | 2.704717 | -0.60273 |
| 14 | 8 | 0 | 1.703348 | 2.134061 | -0.32865 |
| 15 | 8 | 0 | -0.33393 | 0.593845 | 2.05538 |
| 16 | 8 | 0 | 0.39788 | 3.745513 | -1.21079 |
| 17 | 6 | 0 | -1.85033 | 2.192308 | -0.34115 |
| 18 | 8 | 0 | -3.0341 | 1.611331 | -0.05892 |
| 19 | 6 | 0 | -3.08008 | 0.346653 | 0.594954 |
| 20 | 6 | 0 | -4.51449 | 0.021046 | 0.903855 |
| 21 | 6 | 0 | -4.87101 | -1.096539 | 0.265605 |
| 22 | 6 | 0 | -3.68638 | -1.565302 | -0.51573 |
| 23 | 8 | 0 | -2.64515 | -0.682617 | -0.29025 |
| 24 | 8 | 0 | -3.57639 | -2.531407 | -1.2215 |
| 25 | 6 | 0 | -6.16071 | -1.846085 | 0.227991 |
| 26 | 1 | 0 | 3.388609 | -2.900099 | 0.530416 |
| 27 | 1 | 0 | 4.938049 | -2.324043 | 1.133909 |
| 28 | 1 | 0 | 5.08668 | -2.806558 | -1.30536 |
| 29 | 1 | 0 | 5.26652 | -1.074063 | -1.05685 |
| 30 | 1 | 0 | 2.781625 | -2.579614 | -2.01594 |
| 31 | 1 | 0 | 3.656506 | -1.320608 | -2.85783 |
| 32 | 1 | 0 | 2.177267 | -0.372211 | 2.832487 |
| 33 | 1 | 0 | 1.858306 | -1.934457 | 2.076486 |
| 34 | 1 | 0 | 3.34247 | -1.687373 | 3.014808 |
| 35 | 1 | 0 | 4.052633 | 1.181638 | 1.797104 |
| 36 | 1 | 0 | 5.14554 | -0.147002 | 2.198374 |
| 37 | 1 | 0 | 5.113 | 0.520753 | 0.553347 |
| 38 | 1 | 0 | 1.150299 | -0.8849 | -3.02198 |
| 39 | 1 | 0 | 0.504932 | 0.335744 | -1.93079 |
| 40 | 1 | 0 | 1.904718 | 0.707721 | -2.94001 |
| 41 | 1 | 0 | 1.938131 | 1.24975 | 1.489551 |
| 42 | 1 | 0 | -0.24004 | -0.292898 | 0.25835 |
| 43 | 1 | 0 | -0.21816 | 1.479653 | 2.439723 |
| 44 | 1 | 0 | -2.011 | 3.116246 | -0.8911 |
| 45 | 1 | 0 | -2.41052 | 0.354846 | 1.460183 |
| 46 | 1 | 0 | -5.11982 | 0.644992 | 1.550969 |
| 47 | 1 | 0 | -6.01142 | -2.88177 | 0.554342 |
| 48 | 1 | 0 | -6.91588 | -1.378704 | 0.86518 |
| 49 | 1 | 0 | -6.5447 | -1.893909 | -0.79759 |

**Supplementary Data 2.15. Cartesian coordinates of the conformers 1a−1j of 4*S**,5*R**,2'*R** isomer (continued).**

Conformer 1g

| Center Number | Atomic Number | Atomic Type | Coordinates (Angstroms) | | |
|---|---|---|---|---|---|
| | | | X | Y | Z |
| 1 | 6 | 0 | -4.59342 | -1.12038 | -1.38924 |
| 2 | 6 | 0 | -5.77157 | -0.329552 | -0.82766 |
| 3 | 6 | 0 | -5.32646 | 1.101765 | -0.53478 |
| 4 | 6 | 0 | -3.95304 | 1.216109 | 0.0979 |
| 5 | 6 | 0 | -3.07955 | 0.186437 | 0.167381 |
| 6 | 6 | 0 | -3.4028 | -1.214416 | -0.40232 |
| 7 | 6 | 0 | -3.76878 | -2.194877 | 0.743243 |
| 8 | 6 | 0 | -2.22024 | -1.814133 | -1.19811 |
| 9 | 6 | 0 | -3.6861 | 2.613491 | 0.631399 |
| 10 | 6 | 0 | -1.73484 | 0.437998 | 0.839406 |
| 11 | 6 | 0 | -0.54691 | 0.852593 | -0.08305 |
| 12 | 6 | 0 | 0.592455 | 0.054035 | 0.485035 |
| 13 | 6 | 0 | 0.077787 | -0.900488 | 1.486396 |
| 14 | 8 | 0 | -1.27105 | -0.718751 | 1.595326 |
| 15 | 8 | 0 | -0.38654 | 2.266212 | 0.019739 |
| 16 | 8 | 0 | 0.67684 | -1.731629 | 2.128142 |
| 17 | 6 | 0 | 1.896835 | 0.147048 | 0.211028 |
| 18 | 8 | 0 | 2.349716 | 1.066807 | -0.68932 |
| 19 | 6 | 0 | 3.740021 | 1.079866 | -0.97954 |
| 20 | 6 | 0 | 4.644729 | 1.234984 | 0.217267 |
| 21 | 6 | 0 | 5.525221 | 0.230062 | 0.25069 |
| 22 | 6 | 0 | 5.2303 | -0.661172 | -0.9152 |
| 23 | 8 | 0 | 4.144478 | -0.136972 | -1.59615 |
| 24 | 8 | 0 | 5.795405 | -1.660784 | -1.26518 |
| 25 | 6 | 0 | 6.63399 | -0.086162 | 1.197277 |
| 26 | 1 | 0 | -4.90166 | -2.137783 | -1.66287 |
| 27 | 1 | 0 | -4.25226 | -0.635876 | -2.31595 |
| 28 | 1 | 0 | -6.13978 | -0.802682 | 0.090754 |
| 29 | 1 | 0 | -6.61018 | -0.329118 | -1.53486 |
| 30 | 1 | 0 | -6.05345 | 1.607717 | 0.11741 |
| 31 | 1 | 0 | -5.32117 | 1.691208 | -1.46643 |
| 32 | 1 | 0 | -2.93441 | -2.332074 | 1.432619 |
| 33 | 1 | 0 | -4.62307 | -1.831432 | 1.323632 |
| 34 | 1 | 0 | -4.03555 | -3.173984 | 0.324427 |
| 35 | 1 | 0 | -1.3431 | -2.004889 | -0.57556 |
| 36 | 1 | 0 | -2.52393 | -2.776642 | -1.6274 |
| 37 | 1 | 0 | -1.9254 | -1.165327 | -2.03165 |
| 38 | 1 | 0 | -4.04123 | 2.710163 | 1.668151 |
| 39 | 1 | 0 | -2.63762 | 2.910942 | 0.595539 |
| 40 | 1 | 0 | -4.25142 | 3.345954 | 0.042621 |
| 41 | 1 | 0 | -1.83516 | 1.239988 | 1.573419 |
| 42 | 1 | 0 | -0.74572 | 0.576529 | -1.12646 |
| 43 | 1 | 0 | 0.357692 | 2.506792 | -0.55474 |
| 44 | 1 | 0 | 2.630306 | -0.485906 | 0.701737 |
| 45 | 1 | 0 | 3.842179 | 1.88878 | -1.70817 |
| 46 | 1 | 0 | 4.552572 | 2.068574 | 0.90445 |
| 47 | 1 | 0 | 7.582613 | -0.175642 | 0.655657 |
| 48 | 1 | 0 | 6.734999 | 0.680422 | 1.969556 |
| 49 | 1 | 0 | 6.458173 | -1.054018 | 1.680898 |

Conformer 1h

| Center Number | Atomic Number | Atomic Type | Coordinates (Angstroms) | | |
|---|---|---|---|---|---|
| | | | X | Y | Z |
| 1 | 6 | 0 | -4.15575 | -2.047561 | -0.41312 |
| 2 | 6 | 0 | -4.70067 | -1.764874 | 0.984375 |
| 3 | 6 | 0 | -3.53886 | -1.443485 | 1.922696 |
| 4 | 6 | 0 | -2.53192 | -0.472982 | 1.336027 |
| 5 | 6 | 0 | -2.49027 | -0.172709 | 0.020922 |
| 6 | 6 | 0 | -3.41156 | -0.835396 | -1.02367 |
| 7 | 6 | 0 | -2.5903 | -1.371584 | -2.22293 |
| 8 | 6 | 0 | -4.44054 | 0.194566 | -1.55185 |
| 9 | 6 | 0 | -1.62963 | 0.129489 | 2.390364 |
| 10 | 6 | 0 | -1.55332 | 0.890948 | -0.52979 |
| 11 | 6 | 0 | -0.02251 | 0.555604 | -0.57114 |
| 12 | 6 | 0 | 0.597994 | 1.805825 | -0.01809 |
| 13 | 6 | 0 | -0.48072 | 2.73621 | 0.406298 |
| 14 | 8 | 0 | -1.68943 | 2.144194 | 0.191536 |
| 15 | 8 | 0 | 0.409722 | 0.311381 | -1.92025 |
| 16 | 8 | 0 | -0.3812 | 3.836095 | 0.898897 |
| 17 | 6 | 0 | 1.860537 | 2.203938 | 0.22903 |
| 18 | 8 | 0 | 3.059751 | 1.617881 | 0.030011 |
| 19 | 6 | 0 | 3.150941 | 0.34664 | -0.59619 |
| 20 | 6 | 0 | 4.600597 | 0.040438 | -0.84838 |
| 21 | 6 | 0 | 4.952866 | -1.063614 | -0.18445 |
| 22 | 6 | 0 | 3.748276 | -1.545357 | 0.556904 |
| 23 | 8 | 0 | 2.702256 | -0.683092 | 0.287049 |
| 24 | 8 | 0 | 3.62746 | -2.51047 | 1.263542 |
| 25 | 6 | 0 | 6.253576 | -1.789197 | -0.09069 |
| 26 | 1 | 0 | -3.46215 | -2.898692 | -0.35082 |
| 27 | 1 | 0 | -4.95932 | -2.350668 | -1.09682 |
| 28 | 1 | 0 | -5.2673 | -2.625841 | 1.359168 |
| 29 | 1 | 0 | -5.39873 | -0.918611 | 0.95582 |
| 30 | 1 | 0 | -3.01114 | -2.370347 | 2.20084 |
| 31 | 1 | 0 | -3.91182 | -1.029168 | 2.870141 |
| 32 | 1 | 0 | -2.10735 | -0.575737 | -2.80037 |
| 33 | 1 | 0 | -1.82063 | -2.078415 | -1.88467 |
| 34 | 1 | 0 | -3.24851 | -1.91233 | -2.91366 |
| 35 | 1 | 0 | -3.95436 | 1.080328 | -1.97498 |
| 36 | 1 | 0 | -5.05665 | -0.252449 | -2.34217 |
| 37 | 1 | 0 | -5.10537 | 0.539617 | -0.75387 |
| 38 | 1 | 0 | -1.38838 | -0.627621 | 3.147049 |
| 39 | 1 | 0 | -0.6863 | 0.522118 | 2.010064 |
| 40 | 1 | 0 | -2.14 | 0.950774 | 2.910593 |
| 41 | 1 | 0 | -1.82693 | 1.122296 | -1.56161 |
| 42 | 1 | 0 | 0.209527 | -0.320141 | 0.044329 |
| 43 | 1 | 0 | 0.110259 | -0.581746 | -2.15666 |
| 44 | 1 | 0 | 1.999232 | 3.183929 | 0.678206 |
| 45 | 1 | 0 | 2.506827 | 0.329074 | -1.47942 |
| 46 | 1 | 0 | 5.218547 | 0.668437 | -1.47949 |
| 47 | 1 | 0 | 6.133988 | -2.833095 | -0.40281 |
| 48 | 1 | 0 | 7.021282 | -1.319153 | -0.71074 |
| 49 | 1 | 0 | 6.604333 | -1.811861 | 0.947631 |

**Supplementary Data 2.15. Cartesian coordinates of the conformers 1a−1j of 4*S**,5*R**,2'*R** isomer (continued).**

Conformer 1i

| Center Number | Atomic Number | Atomic Type | Coordinates (Angstroms) | | |
|---|---|---|---|---|---|
| | | | X | Y | Z |
| 1 | 6 | 0 | 4.697358 | -1.426725 | 0.357251 |
| 2 | 6 | 0 | 4.083646 | -2.552998 | -0.47023 |
| 3 | 6 | 0 | 3.323827 | -1.949147 | -1.64953 |
| 4 | 6 | 0 | 2.41886 | -0.796869 | -1.25845 |
| 5 | 6 | 0 | 2.534039 | -0.149538 | -0.07895 |
| 6 | 6 | 0 | 3.639889 | -0.464331 | 0.950811 |
| 7 | 6 | 0 | 3.015465 | -1.100805 | 2.217545 |
| 8 | 6 | 0 | 4.387564 | 0.82927 | 1.359805 |
| 9 | 6 | 0 | 1.409001 | -0.466696 | -2.33396 |
| 10 | 6 | 0 | 1.565984 | 0.94567 | 0.339158 |
| 11 | 6 | 0 | 0.049412 | 0.580496 | 0.415477 |
| 12 | 6 | 0 | -0.60285 | 1.81631 | -0.13649 |
| 13 | 6 | 0 | 0.452447 | 2.714141 | -0.67888 |
| 14 | 8 | 0 | 1.670757 | 2.121793 | -0.51134 |
| 15 | 8 | 0 | -0.32984 | 0.326402 | 1.778994 |
| 16 | 8 | 0 | 0.327297 | 3.78501 | -1.226 |
| 17 | 6 | 0 | -1.87514 | 2.215578 | -0.32421 |
| 18 | 8 | 0 | -3.06578 | 1.649934 | -0.03249 |
| 19 | 6 | 0 | -3.13186 | 0.406429 | 0.648514 |
| 20 | 6 | 0 | -4.57048 | 0.111262 | 0.968152 |
| 21 | 6 | 0 | -4.94568 | -1.020794 | 0.366769 |
| 22 | 6 | 0 | -3.76915 | -1.534145 | -0.3979 |
| 23 | 8 | 0 | -2.71544 | -0.660872 | -0.20608 |
| 24 | 8 | 0 | -3.67279 | -2.529349 | -1.06574 |
| 25 | 6 | 0 | -6.24764 | -1.750114 | 0.354416 |
| 26 | 1 | 0 | 5.311742 | -1.825946 | 1.174655 |
| 27 | 1 | 0 | 5.375541 | -0.851637 | -0.28901 |
| 28 | 1 | 0 | 3.398715 | -3.15098 | 0.145465 |
| 29 | 1 | 0 | 4.861788 | -3.238263 | -0.82755 |
| 30 | 1 | 0 | 2.71818 | -2.715501 | -2.15387 |
| 31 | 1 | 0 | 4.035664 | -1.594296 | -2.41232 |
| 32 | 1 | 0 | 2.234139 | -0.464625 | 2.650437 |
| 33 | 1 | 0 | 2.569311 | -2.077402 | 1.995729 |
| 34 | 1 | 0 | 3.779379 | -1.248071 | 2.990959 |
| 35 | 1 | 0 | 3.765418 | 1.520341 | 1.937422 |
| 36 | 1 | 0 | 5.250656 | 0.572184 | 1.986138 |
| 37 | 1 | 0 | 4.754356 | 1.365431 | 0.477941 |
| 38 | 1 | 0 | 0.664595 | -1.269221 | -2.43257 |
| 39 | 1 | 0 | 0.88436 | 0.475166 | -2.1835 |
| 40 | 1 | 0 | 1.918943 | -0.400073 | -3.30439 |
| 41 | 1 | 0 | 1.815162 | 1.288693 | 1.345305 |
| 42 | 1 | 0 | -0.19021 | -0.29929 | -0.1891 |
| 43 | 1 | 0 | -0.07272 | -0.588725 | 1.977037 |
| 44 | 1 | 0 | -2.03348 | 3.177627 | -0.80453 |
| 45 | 1 | 0 | -2.45379 | 0.424441 | 1.505545 |
| 46 | 1 | 0 | -5.16543 | 0.765917 | 1.594249 |
| 47 | 1 | 0 | -6.11414 | -2.779656 | 0.706073 |
| 48 | 1 | 0 | -6.99173 | -1.254141 | 0.982993 |
| 49 | 1 | 0 | -6.63822 | -1.817282 | -0.66764 |

Conformer 1j

| Center Number | Atomic Number | Atomic Type | Coordinates (Angstroms) | | |
|---|---|---|---|---|---|
| | | | X | Y | Z |
| 1 | 6 | 0 | 3.635346 | -1.591472 | -1.49271 |
| 2 | 6 | 0 | 4.668855 | -2.0445 | -0.46587 |
| 3 | 6 | 0 | 3.990861 | -2.211874 | 0.892146 |
| 4 | 6 | 0 | 3.004711 | -1.111928 | 1.241532 |
| 5 | 6 | 0 | 2.541709 | -0.215588 | 0.339417 |
| 6 | 6 | 0 | 2.990575 | -0.227634 | -1.14122 |
| 7 | 6 | 0 | 4.022192 | 0.903695 | -1.39365 |
| 8 | 6 | 0 | 1.807182 | -0.036566 | -2.11823 |
| 9 | 6 | 0 | 2.620977 | -1.168516 | 2.708975 |
| 10 | 6 | 0 | 1.54115 | 0.833706 | 0.807431 |
| 11 | 6 | 0 | 0.026609 | 0.474823 | 0.639304 |
| 12 | 6 | 0 | -0.57087 | 1.786221 | 0.2139 |
| 13 | 6 | 0 | 0.533044 | 2.737968 | -0.07351 |
| 14 | 8 | 0 | 1.727358 | 2.117909 | 0.15952 |
| 15 | 8 | 0 | -0.51068 | -0.006189 | 1.882019 |
| 16 | 8 | 0 | 0.47114 | 3.87487 | -0.47915 |
| 17 | 6 | 0 | -1.81484 | 2.226099 | -0.05554 |
| 18 | 8 | 0 | -3.03139 | 1.645887 | 0.023115 |
| 19 | 6 | 0 | -3.18729 | 0.346996 | 0.572344 |
| 20 | 6 | 0 | -4.65611 | 0.043693 | 0.672014 |
| 21 | 6 | 0 | -4.95018 | -1.025789 | -0.07205 |
| 22 | 6 | 0 | -3.68327 | -1.486302 | -0.71638 |
| 23 | 8 | 0 | -2.66249 | -0.646947 | -0.31 |
| 24 | 8 | 0 | -3.49981 | -2.421048 | -1.44971 |
| 25 | 6 | 0 | -6.2402 | -1.733513 | -0.32121 |
| 26 | 1 | 0 | 4.08417 | -1.517761 | -2.49156 |
| 27 | 1 | 0 | 2.845669 | -2.354205 | -1.55995 |
| 28 | 1 | 0 | 5.477472 | -1.307287 | -0.39221 |
| 29 | 1 | 0 | 5.134979 | -2.988027 | -0.77507 |
| 30 | 1 | 0 | 4.741047 | -2.276887 | 1.693705 |
| 31 | 1 | 0 | 3.454237 | -3.174433 | 0.924989 |
| 32 | 1 | 0 | 3.588498 | 1.887201 | -1.20708 |
| 33 | 1 | 0 | 4.899451 | 0.7981 | -0.74705 |
| 34 | 1 | 0 | 4.363442 | 0.868694 | -2.43634 |
| 35 | 1 | 0 | 1.324841 | 0.93773 | -2.01372 |
| 36 | 1 | 0 | 2.176818 | -0.103418 | -3.14858 |
| 37 | 1 | 0 | 1.049596 | -0.819674 | -1.99518 |
| 38 | 1 | 0 | 3.455719 | -0.823616 | 3.335823 |
| 39 | 1 | 0 | 1.734658 | -0.595001 | 2.980803 |
| 40 | 1 | 0 | 2.435824 | -2.211551 | 3.000027 |
| 41 | 1 | 0 | 1.68206 | 1.028503 | 1.87165 |
| 42 | 1 | 0 | -0.12238 | -0.288249 | -0.13016 |
| 43 | 1 | 0 | -0.37954 | -0.967486 | 1.891917 |
| 44 | 1 | 0 | -1.91833 | 3.246397 | -0.41566 |
| 45 | 1 | 0 | -2.62789 | 0.28053 | 1.508524 |
| 46 | 1 | 0 | -5.32785 | 0.649049 | 1.269357 |
| 47 | 1 | 0 | -6.15615 | -2.791812 | -0.04853 |
| 48 | 1 | 0 | -7.06018 | -1.285029 | 0.245553 |
| 49 | 1 | 0 | -6.4919 | -1.704723 | -1.38772 |

**Supplementary Data 2.16. Energy analysis and Boltzmann weights of low-energy conformers of 4*R**,5*S**,2'*R** isomer optimized in the gas phase at the B3LYP/6-31G(d) level for DP4+ analysis.**

| Conformer | Total energy (au) | ΔE (kJ/mol) | Gibbs free energy (au) | ΔG (kJ/mol) | Boltzmann weights |
|---|---|---|---|---|---|
| 2a | -1189.782355 | 0.00 | -1189.431820 | 0.00 | 0.310 |
| 2b | -1189.782220 | 0.35 | -1189.431733 | 0.23 | 0.283 |
| 2c | -1189.781416 | 2.46 | -1189.431272 | 1.44 | 0.174 |
| 2d | -1189.781055 | 3.41 | -1189.430261 | 4.09 | 0.059 |
| 2e | -1189.780884 | 3.86 | -1189.430143 | 4.40 | 0.052 |
| 2f | -1189.780644 | 4.49 | -1189.429827 | 5.23 | 0.038 |
| 2g | -1189.780240 | 5.55 | -1189.429819 | 5.25 | 0.037 |
| 2h | -1189.780241 | 5.55 | -1189.429804 | 5.29 | 0.037 |
| 2i | -1189.779450 | 7.63 | -1189.428545 | 8.60 | 0.010 |

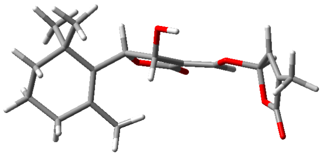

2a (31.0%)

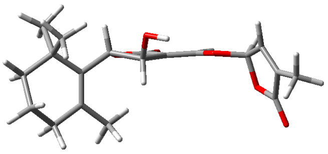

2b (28.3%)

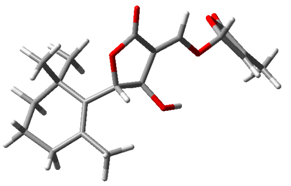

2c (17.4%)

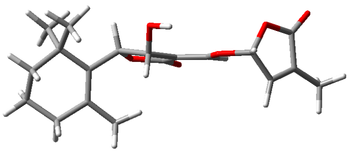

2d (5.9%)

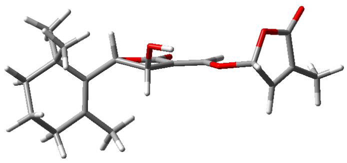

2e (5.2%)

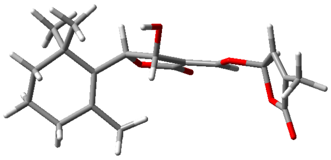

2f (3.8%)

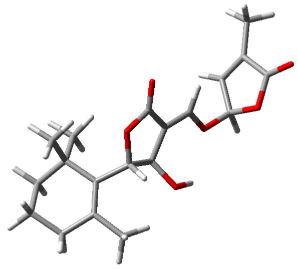

2g (3.7%)

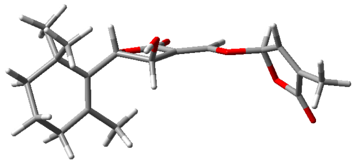

2h (3.7%)

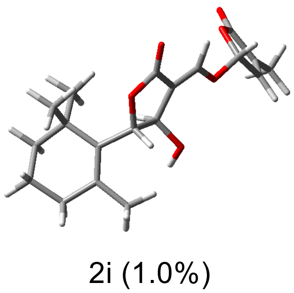

2i (1.0%)

**Supplementary Data 2.17. Low-energy conformers of 4*R**,5*S**,2'*R** isomer optimized in the gas phase at the B3LYP/6-31G(d) level for DP4+ analysis.**

**Supplementary Data 2.18. Cartesian coordinates of the conformers 2a−2i of 4*R**,5*S**,2'*R** isomer.**

Conformer 2a

| Center Number | Atomic Number | Atomic Type | Coordinates (Angstroms) X | Y | Z |
|---|---|---|---|---|---|
| 1 | 6 | 0 | 4.858433 | -1.789255 | 0.287557 |
| 2 | 6 | 0 | 5.318275 | -0.972694 | 1.492257 |
| 3 | 6 | 0 | 4.097653 | -0.544172 | 2.304562 |
| 4 | 6 | 0 | 2.9655 | 0.005591 | 1.457668 |
| 5 | 6 | 0 | 2.906921 | -0.166112 | 0.120457 |
| 6 | 6 | 0 | 3.943763 | -0.988909 | -0.67106 |
| 7 | 6 | 0 | 4.808456 | -0.044245 | -1.54274 |
| 8 | 6 | 0 | 3.245831 | -2.018128 | -1.59348 |
| 9 | 6 | 0 | 1.961964 | 0.784842 | 2.279745 |
| 10 | 6 | 0 | 1.832344 | 0.49921 | -0.72477 |
| 11 | 6 | 0 | 0.371857 | -0.032 | -0.60436 |
| 12 | 6 | 0 | -0.42244 | 1.243242 | -0.53065 |
| 13 | 6 | 0 | 0.493994 | 2.399734 | -0.43086 |
| 14 | 8 | 0 | 1.774087 | 1.934383 | -0.45845 |
| 15 | 8 | 0 | 0.100536 | -0.831149 | -1.75313 |
| 16 | 8 | 0 | 0.224737 | 3.573946 | -0.32601 |
| 17 | 6 | 0 | -1.74779 | 1.40006 | -0.57891 |
| 18 | 8 | 0 | -2.57345 | 0.324837 | -0.73946 |
| 19 | 6 | 0 | -3.9599 | 0.567268 | -0.58619 |
| 20 | 6 | 0 | -4.73851 | -0.607953 | -1.10894 |
| 21 | 6 | 0 | -5.46173 | -1.140114 | -0.1205 |
| 22 | 6 | 0 | -5.19023 | -0.341294 | 1.115961 |
| 23 | 8 | 0 | -4.30082 | 0.666716 | 0.791364 |
| 24 | 8 | 0 | -5.63878 | -0.483558 | 2.220645 |
| 25 | 6 | 0 | -6.39246 | -2.305501 | -0.0915 |
| 26 | 1 | 0 | 5.716144 | -2.17245 | -0.28034 |
| 27 | 1 | 0 | 4.308629 | -2.669106 | 0.651809 |
| 28 | 1 | 0 | 5.872216 | -0.085022 | 1.161126 |
| 29 | 1 | 0 | 6.006007 | -1.556448 | 2.116327 |
| 30 | 1 | 0 | 4.377178 | 0.214627 | 3.049436 |
| 31 | 1 | 0 | 3.716805 | -1.397141 | 2.89006 |
| 32 | 1 | 0 | 4.198648 | 0.556934 | -2.226 |
| 33 | 1 | 0 | 5.388926 | 0.651411 | -0.9282 |
| 34 | 1 | 0 | 5.508005 | -0.627303 | -2.15477 |
| 35 | 1 | 0 | 2.634538 | -1.555062 | -2.37295 |
| 36 | 1 | 0 | 4.003859 | -2.636768 | -2.09012 |
| 37 | 1 | 0 | 2.592265 | -2.682608 | -1.01705 |
| 38 | 1 | 0 | 0.98077 | 0.894902 | 1.8172 |
| 39 | 1 | 0 | 1.814588 | 0.291327 | 3.249026 |
| 40 | 1 | 0 | 2.337386 | 1.794924 | 2.490572 |
| 41 | 1 | 0 | 2.08692 | 0.409237 | -1.78277 |
| 42 | 1 | 0 | 0.248254 | -0.635278 | 0.305043 |
| 43 | 1 | 0 | -0.82022 | -1.128314 | -1.67549 |
| 44 | 1 | 0 | -2.21542 | 2.381343 | -0.52183 |
| 45 | 1 | 0 | -4.21415 | 1.522404 | -1.06263 |
| 46 | 1 | 0 | -4.6728 | -0.929168 | -2.142 |
| 47 | 1 | 0 | -7.38223 | -1.994552 | 0.262007 |
| 48 | 1 | 0 | -6.49524 | -2.76372 | -1.07842 |
| 49 | 1 | 0 | -6.03316 | -3.062981 | 0.614691 |

Conformer 2b

| Center Number | Atomic Number | Atomic Type | Coordinates (Angstroms) X | Y | Z |
|---|---|---|---|---|---|
| 1 | 6 | 0 | 5.283751 | -1.122459 | -0.15387 |
| 2 | 6 | 0 | 4.858803 | -2.167475 | 0.873933 |
| 3 | 6 | 0 | 4.010406 | -1.494063 | 1.950661 |
| 4 | 6 | 0 | 2.936643 | -0.579699 | 1.392323 |
| 5 | 6 | 0 | 2.950624 | -0.134791 | 0.118054 |
| 6 | 6 | 0 | 4.085434 | -0.46017 | -0.87615 |
| 7 | 6 | 0 | 4.623569 | 0.834431 | -1.53611 |
| 8 | 6 | 0 | 3.557172 | -1.405357 | -1.98359 |
| 9 | 6 | 0 | 1.88938 | -0.231665 | 2.425891 |
| 10 | 6 | 0 | 1.83423 | 0.726487 | -0.45041 |
| 11 | 6 | 0 | 0.39071 | 0.149302 | -0.45876 |
| 12 | 6 | 0 | -0.42741 | 1.359221 | -0.09694 |
| 13 | 6 | 0 | 0.467914 | 2.455006 | 0.334979 |
| 14 | 8 | 0 | 1.756954 | 2.019834 | 0.229101 |
| 15 | 8 | 0 | 0.144831 | -0.371215 | -1.76257 |
| 16 | 8 | 0 | 0.178891 | 3.557014 | 0.739515 |
| 17 | 6 | 0 | -1.75216 | 1.516069 | -0.15619 |
| 18 | 8 | 0 | -2.55516 | 0.511394 | -0.61468 |
| 19 | 6 | 0 | -3.94983 | 0.701803 | -0.46589 |
| 20 | 6 | 0 | -4.6889 | -0.304103 | -1.3042 |
| 21 | 6 | 0 | -5.44499 | -1.07302 | -0.51657 |
| 22 | 6 | 0 | -5.23696 | -0.614719 | 0.89318 |
| 23 | 8 | 0 | -4.34943 | 0.445472 | 0.875417 |
| 24 | 8 | 0 | -5.72899 | -1.035705 | 1.904399 |
| 25 | 6 | 0 | -6.35919 | -2.210446 | -0.82604 |
| 26 | 1 | 0 | 5.863234 | -0.343205 | 0.361844 |
| 27 | 1 | 0 | 5.951778 | -1.559441 | -0.90743 |
| 28 | 1 | 0 | 5.736545 | -2.646955 | 1.324268 |
| 29 | 1 | 0 | 4.279508 | -2.964499 | 0.389986 |
| 30 | 1 | 0 | 4.655858 | -0.907233 | 2.624329 |
| 31 | 1 | 0 | 3.531996 | -2.247155 | 2.593268 |
| 32 | 1 | 0 | 3.909257 | 1.294342 | -2.22652 |
| 33 | 1 | 0 | 4.890945 | 1.581257 | -0.78035 |
| 34 | 1 | 0 | 5.524062 | 0.600613 | -2.11713 |
| 35 | 1 | 0 | 2.655195 | -1.011791 | -2.4638 |
| 36 | 1 | 0 | 4.320373 | -1.546383 | -2.75952 |
| 37 | 1 | 0 | 3.300669 | -2.389819 | -1.57699 |
| 38 | 1 | 0 | 1.204998 | 0.56026 | 2.127612 |
| 39 | 1 | 0 | 1.299683 | -1.119049 | 2.696698 |
| 40 | 1 | 0 | 2.382507 | 0.101121 | 3.349263 |
| 41 | 1 | 0 | 2.044157 | 0.955045 | -1.49672 |
| 42 | 1 | 0 | 0.274284 | -0.647411 | 0.287176 |
| 43 | 1 | 0 | -0.76621 | -0.705706 | -1.76482 |
| 44 | 1 | 0 | -2.23701 | 2.447958 | 0.128946 |
| 45 | 1 | 0 | -4.19827 | 1.745862 | -0.69419 |
| 46 | 1 | 0 | -4.57525 | -0.351216 | -2.38105 |
| 47 | 1 | 0 | -7.36807 | -2.001675 | -0.45201 |
| 48 | 1 | 0 | -6.41156 | -2.404533 | -1.90035 |
| 49 | 1 | 0 | -6.02075 | -3.120844 | -0.31791 |

**Supplementary Data 2.18. Cartesian coordinates of the conformers 2a−2i of 4*R**,5*S**,2'*R** isomer (continued).**

Conformer 2c

| Center Number | Atomic Number | Atomic Type | Coordinates (Angstroms) | | |
|---|---|---|---|---|---|
| | | | X | Y | Z |
| 1 | 6 | 0 | 4.161291 | -0.761566 | 2.005806 |
| 2 | 6 | 0 | 5.301253 | -1.37364 | 1.196579 |
| 3 | 6 | 0 | 4.726114 | -2.104646 | -0.01482 |
| 4 | 6 | 0 | 3.595155 | -1.373533 | -0.71194 |
| 5 | 6 | 0 | 2.964712 | -0.302156 | -0.18028 |
| 6 | 6 | 0 | 3.339949 | 0.27834 | 1.203052 |
| 7 | 6 | 0 | 2.096135 | 0.610251 | 2.059872 |
| 8 | 6 | 0 | 4.180209 | 1.572317 | 1.03862 |
| 9 | 6 | 0 | 3.262864 | -1.989735 | -2.06055 |
| 10 | 6 | 0 | 1.849435 | 0.342923 | -0.99461 |
| 11 | 6 | 0 | 0.393412 | -0.141921 | -0.71365 |
| 12 | 6 | 0 | -0.38227 | 1.145314 | -0.74152 |
| 13 | 6 | 0 | 0.553212 | 2.286479 | -0.79661 |
| 14 | 8 | 0 | 1.824998 | 1.792528 | -0.84421 |
| 15 | 8 | 0 | 0.035778 | -1.069827 | -1.73567 |
| 16 | 8 | 0 | 0.313777 | 3.471128 | -0.79061 |
| 17 | 6 | 0 | -1.70618 | 1.319816 | -0.75012 |
| 18 | 8 | 0 | -2.55169 | 0.248268 | -0.76144 |
| 19 | 6 | 0 | -3.92849 | 0.529524 | -0.5871 |
| 20 | 6 | 0 | -4.74102 | -0.680818 | -0.95539 |
| 21 | 6 | 0 | -5.435 | -1.093519 | 0.108256 |
| 22 | 6 | 0 | -5.10897 | -0.173134 | 1.242898 |
| 23 | 8 | 0 | -4.21813 | 0.77953 | 0.782857 |
| 24 | 8 | 0 | -5.51906 | -0.190606 | 2.371213 |
| 25 | 6 | 0 | -6.37946 | -2.233312 | 0.29279 |
| 26 | 1 | 0 | 3.490671 | -1.568018 | 2.337238 |
| 27 | 1 | 0 | 4.543528 | -0.278707 | 2.914476 |
| 28 | 1 | 0 | 5.887068 | -2.065359 | 1.814422 |
| 29 | 1 | 0 | 5.993301 | -0.58921 | 0.86692 |
| 30 | 1 | 0 | 4.353159 | -3.096991 | 0.287934 |
| 31 | 1 | 0 | 5.5148 | -2.310702 | -0.75329 |
| 32 | 1 | 0 | 1.481107 | 1.402004 | 1.625984 |
| 33 | 1 | 0 | 1.468741 | -0.274998 | 2.219145 |
| 34 | 1 | 0 | 2.419838 | 0.960796 | 3.047417 |
| 35 | 1 | 0 | 3.615166 | 2.352342 | 0.526247 |
| 36 | 1 | 0 | 4.476688 | 1.951839 | 2.02516 |
| 37 | 1 | 0 | 5.090991 | 1.387261 | 0.459904 |
| 38 | 1 | 0 | 2.212325 | -1.906108 | -2.34085 |
| 39 | 1 | 0 | 3.873218 | -1.538957 | -2.85715 |
| 40 | 1 | 0 | 3.511821 | -3.057673 | -2.04847 |
| 41 | 1 | 0 | 2.021518 | 0.16646 | -2.05826 |
| 42 | 1 | 0 | 0.324184 | -0.626263 | 0.268765 |
| 43 | 1 | 0 | -0.87489 | -1.351416 | -1.55323 |
| 44 | 1 | 0 | -2.15851 | 2.309516 | -0.77558 |
| 45 | 1 | 0 | -4.18658 | 1.433198 | -1.15332 |
| 46 | 1 | 0 | -4.71683 | -1.110126 | -1.9503 |
| 47 | 1 | 0 | -7.35317 | -1.869412 | 0.640377 |
| 48 | 1 | 0 | -6.52016 | -2.794296 | -0.63459 |
| 49 | 1 | 0 | -6.00757 | -2.915248 | 1.066215 |

Conformer 2d

| Center Number | Atomic Number | Atomic Type | Coordinates (Angstroms) | | |
|---|---|---|---|---|---|
| | | | X | Y | Z |
| 1 | 6 | 0 | -5.39658 | -0.531074 | -0.7055 |
| 2 | 6 | 0 | -5.73138 | 0.951591 | -0.56642 |
| 3 | 6 | 0 | -4.55292 | 1.782379 | -1.07093 |
| 4 | 6 | 0 | -3.20707 | 1.300639 | -0.56292 |
| 5 | 6 | 0 | -3.03313 | 0.087709 | 0.002107 |
| 6 | 6 | 0 | -4.16389 | -0.953217 | 0.129884 |
| 7 | 6 | 0 | -4.57243 | -1.103273 | 1.616646 |
| 8 | 6 | 0 | -3.70804 | -2.333564 | -0.40326 |
| 9 | 6 | 0 | -2.12462 | 2.345032 | -0.73049 |
| 10 | 6 | 0 | -1.70551 | -0.32618 | 0.616621 |
| 11 | 6 | 0 | -0.5131 | -0.621332 | -0.3442 |
| 12 | 6 | 0 | 0.625616 | 0.07877 | 0.345362 |
| 13 | 6 | 0 | 0.113329 | 0.864852 | 1.488106 |
| 14 | 8 | 0 | -1.23415 | 0.680676 | 1.564649 |
| 15 | 8 | 0 | -0.36499 | -2.034773 | -0.43574 |
| 16 | 8 | 0 | 0.721657 | 1.583715 | 2.247158 |
| 17 | 6 | 0 | 1.930427 | 0.022164 | 0.063213 |
| 18 | 8 | 0 | 2.374716 | -0.709741 | -1.0001 |
| 19 | 6 | 0 | 3.775606 | -0.767428 | -1.2264 |
| 20 | 6 | 0 | 4.460716 | 0.569806 | -1.35521 |
| 21 | 6 | 0 | 5.463854 | 0.649575 | -0.47569 |
| 22 | 6 | 0 | 5.47845 | -0.629118 | 0.302565 |
| 23 | 8 | 0 | 4.440566 | -1.424215 | -0.15329 |
| 24 | 8 | 0 | 6.229628 | -0.976525 | 1.172056 |
| 25 | 6 | 0 | 6.454601 | 1.729131 | -0.19598 |
| 26 | 1 | 0 | -6.25048 | -1.157268 | -0.41629 |
| 27 | 1 | 0 | -5.19829 | -0.746042 | -1.76549 |
| 28 | 1 | 0 | -5.93557 | 1.198786 | 0.483146 |
| 29 | 1 | 0 | -6.64025 | 1.198176 | -1.12891 |
| 30 | 1 | 0 | -4.67626 | 2.837797 | -0.78838 |
| 31 | 1 | 0 | -4.5348 | 1.778628 | -2.17333 |
| 32 | 1 | 0 | -3.72388 | -1.386626 | 2.248851 |
| 33 | 1 | 0 | -4.97972 | -0.169973 | 2.018675 |
| 34 | 1 | 0 | -5.33529 | -1.884661 | 1.722997 |
| 35 | 1 | 0 | -2.88842 | -2.770737 | 0.173485 |
| 36 | 1 | 0 | -4.55056 | -3.035514 | -0.36663 |
| 37 | 1 | 0 | -3.37317 | -2.261861 | -1.44424 |
| 38 | 1 | 0 | -1.10782 | 1.951233 | -0.72124 |
| 39 | 1 | 0 | -2.2648 | 2.873723 | -1.68236 |
| 40 | 1 | 0 | -2.18878 | 3.098982 | 0.065213 |
| 41 | 1 | 0 | -1.83757 | -1.238485 | 1.201413 |
| 42 | 1 | 0 | -0.70244 | -0.201452 | -1.3419 |
| 43 | 1 | 0 | 0.448304 | -2.202152 | -0.93892 |
| 44 | 1 | 0 | 2.668857 | 0.541612 | 0.66665 |
| 45 | 1 | 0 | 3.865055 | -1.388295 | -2.12204 |
| 46 | 1 | 0 | 4.147401 | 1.309273 | -2.0836 |
| 47 | 1 | 0 | 7.47561 | 1.342378 | -0.2918 |
| 48 | 1 | 0 | 6.328907 | 2.576737 | -0.87424 |
| 49 | 1 | 0 | 6.350774 | 2.083874 | 0.83599 |

**Supplementary Data 2.18. Cartesian coordinates of the conformers 2a−2i of 4*R**,5*S**,2'*R** isomer (continued).**

Conformer 2e

| Center Number | Atomic Number | Atomic Type | Coordinates (Angstroms) | | |
|---|---|---|---|---|---|
| | | | X | Y | Z |
| 1 | 6 | 0 | -5.56807 | -0.28479 | -0.01305 |
| 2 | 6 | 0 | -5.57092 | 0.649967 | -1.21939 |
| 3 | 6 | 0 | -4.60041 | 1.800804 | -0.96234 |
| 4 | 6 | 0 | -3.25483 | 1.349482 | -0.42692 |
| 5 | 6 | 0 | -3.05471 | 0.121776 | 0.096837 |
| 6 | 6 | 0 | -4.18251 | -0.919434 | 0.258734 |
| 7 | 6 | 0 | -4.21931 | -1.465637 | 1.708349 |
| 8 | 6 | 0 | -3.94963 | -2.100062 | -0.71622 |
| 9 | 6 | 0 | -2.20375 | 2.43052 | -0.54071 |
| 10 | 6 | 0 | -1.68652 | -0.344429 | 0.56787 |
| 11 | 6 | 0 | -0.52528 | -0.384729 | -0.46638 |
| 12 | 6 | 0 | 0.637269 | 0.094187 | 0.360167 |
| 13 | 6 | 0 | 0.147034 | 0.618121 | 1.653613 |
| 14 | 8 | 0 | -1.20722 | 0.457202 | 1.694335 |
| 15 | 8 | 0 | -0.41332 | -1.725335 | -0.93271 |
| 16 | 8 | 0 | 0.773378 | 1.135657 | 2.549288 |
| 17 | 6 | 0 | 1.940066 | 0.060081 | 0.066335 |
| 18 | 8 | 0 | 2.362522 | -0.417609 | -1.14122 |
| 19 | 6 | 0 | 3.761448 | -0.474905 | -1.37839 |
| 20 | 6 | 0 | 4.498109 | 0.827679 | -1.19147 |
| 21 | 6 | 0 | 5.498019 | 0.659865 | -0.32071 |
| 22 | 6 | 0 | 5.458723 | -0.76538 | 0.135414 |
| 23 | 8 | 0 | 4.394048 | -1.390444 | -0.4913 |
| 24 | 8 | 0 | 6.19052 | -1.335929 | 0.896977 |
| 25 | 6 | 0 | 6.527351 | 1.60465 | 0.201927 |
| 26 | 1 | 0 | -5.87658 | 0.289456 | 0.872432 |
| 27 | 1 | 0 | -6.30584 | -1.088339 | -0.13529 |
| 28 | 1 | 0 | -6.58003 | 1.036599 | -1.40752 |
| 29 | 1 | 0 | -5.26934 | 0.105836 | -2.12382 |
| 30 | 1 | 0 | -5.0433 | 2.512073 | -0.24619 |
| 31 | 1 | 0 | -4.43692 | 2.381318 | -1.88183 |
| 32 | 1 | 0 | -3.35863 | -2.096451 | 1.952358 |
| 33 | 1 | 0 | -4.25666 | -0.64874 | 2.437593 |
| 34 | 1 | 0 | -5.11598 | -2.082941 | 1.842684 |
| 35 | 1 | 0 | -2.94592 | -2.525586 | -0.61446 |
| 36 | 1 | 0 | -4.67732 | -2.899148 | -0.52584 |
| 37 | 1 | 0 | -4.0593 | -1.782443 | -1.759 |
| 38 | 1 | 0 | -1.27664 | 2.216105 | -0.0124 |
| 39 | 1 | 0 | -1.96715 | 2.63304 | -1.59518 |
| 40 | 1 | 0 | -2.59814 | 3.369836 | -0.12969 |
| 41 | 1 | 0 | -1.75537 | -1.367369 | 0.941264 |
| 42 | 1 | 0 | -0.721 | 0.288438 | -1.31174 |
| 43 | 1 | 0 | 0.384449 | -1.769646 | -1.48409 |
| 44 | 1 | 0 | 2.694506 | 0.39973 | 0.769632 |
| 45 | 1 | 0 | 3.833138 | -0.871129 | -2.39503 |
| 46 | 1 | 0 | 4.217915 | 1.729093 | -1.72483 |
| 47 | 1 | 0 | 7.53347 | 1.211339 | 0.016932 |
| 48 | 1 | 0 | 6.439429 | 2.591441 | -0.25938 |
| 49 | 1 | 0 | 6.429261 | 1.712551 | 1.288359 |

Conformer 2f

| Center Number | Atomic Number | Atomic Type | Coordinates (Angstroms) | | |
|---|---|---|---|---|---|
| | | | X | Y | Z |
| 1 | 6 | 0 | 4.917142 | -1.714127 | 0.406052 |
| 2 | 6 | 0 | 5.342174 | -0.84282 | 1.585036 |
| 3 | 6 | 0 | 4.100685 | -0.403018 | 2.35895 |
| 4 | 6 | 0 | 2.973756 | 0.091493 | 1.472272 |
| 5 | 6 | 0 | 2.944645 | -0.127319 | 0.140727 |
| 6 | 6 | 0 | 4.009011 | -0.964089 | -0.59828 |
| 7 | 6 | 0 | 4.875337 | -0.046198 | -1.49597 |
| 8 | 6 | 0 | 3.340563 | -2.039743 | -1.49019 |
| 9 | 6 | 0 | 1.935733 | 0.876857 | 2.244023 |
| 10 | 6 | 0 | 1.871338 | 0.479201 | -0.74822 |
| 11 | 6 | 0 | 0.422235 | -0.11793 | -0.65493 |
| 12 | 6 | 0 | -0.41917 | 1.121365 | -0.64395 |
| 13 | 6 | 0 | 0.444271 | 2.318334 | -0.56839 |
| 14 | 8 | 0 | 1.747331 | 1.913273 | -0.52494 |
| 15 | 8 | 0 | 0.107996 | -0.937725 | -1.77821 |
| 16 | 8 | 0 | 0.124635 | 3.483966 | -0.52315 |
| 17 | 6 | 0 | -1.75173 | 1.251122 | -0.67211 |
| 18 | 8 | 0 | -2.5857 | 0.186343 | -0.72894 |
| 19 | 6 | 0 | -3.96887 | 0.469931 | -0.65667 |
| 20 | 6 | 0 | -4.75072 | -0.750822 | -1.05508 |
| 21 | 6 | 0 | -5.51977 | -1.14151 | -0.0359 |
| 22 | 6 | 0 | -5.27793 | -0.196739 | 1.098703 |
| 23 | 8 | 0 | -4.35747 | 0.747849 | 0.685377 |
| 24 | 8 | 0 | -5.76946 | -0.191153 | 2.194951 |
| 25 | 6 | 0 | -6.47182 | -2.281631 | 0.104927 |
| 26 | 1 | 0 | 5.79059 | -2.106567 | -0.13065 |
| 27 | 1 | 0 | 4.373898 | -2.586992 | 0.796557 |
| 28 | 1 | 0 | 5.887694 | 0.039833 | 1.227907 |
| 29 | 1 | 0 | 6.028195 | -1.390356 | 2.242653 |
| 30 | 1 | 0 | 4.354621 | 0.390877 | 3.075727 |
| 31 | 1 | 0 | 3.725006 | -1.237326 | 2.973903 |
| 32 | 1 | 0 | 4.269114 | 0.516506 | -2.21397 |
| 33 | 1 | 0 | 5.433681 | 0.68296 | -0.90056 |
| 34 | 1 | 0 | 5.595067 | -0.643601 | -2.06959 |
| 35 | 1 | 0 | 2.737048 | -1.612355 | -2.29761 |
| 36 | 1 | 0 | 4.10963 | -2.666317 | -1.95796 |
| 37 | 1 | 0 | 2.700695 | -2.700609 | -0.89007 |
| 38 | 1 | 0 | 0.960388 | 0.936958 | 1.760466 |
| 39 | 1 | 0 | 1.787162 | 0.422518 | 3.231996 |
| 40 | 1 | 0 | 2.27809 | 1.906025 | 2.414155 |
| 41 | 1 | 0 | 2.157166 | 0.373752 | -1.79726 |
| 42 | 1 | 0 | 0.299543 | -0.696012 | 0.270552 |
| 43 | 1 | 0 | 0.484832 | -1.815452 | -1.61118 |
| 44 | 1 | 0 | -2.2077 | 2.240193 | -0.65317 |
| 45 | 1 | 0 | -4.1918 | 1.360613 | -1.25758 |
| 46 | 1 | 0 | -4.64599 | -1.205008 | -2.03331 |
| 47 | 1 | 0 | -7.46952 | -1.91573 | 0.373487 |
| 48 | 1 | 0 | -6.54322 | -2.862523 | -0.8182 |
| 49 | 1 | 0 | -6.15426 | -2.945956 | 0.916962 |

**Supplementary Data 2.18. Cartesian coordinates of the conformers 2a−2i of 4*R**,5*S**,2'*R** isomer (continued).**

Conformer 2g

| Center Number | Atomic Number | Atomic Type | Coordinates (Angstroms) | | |
|---|---|---|---|---|---|
| | | | X | Y | Z |
| 1 | 6 | 0 | 4.686044 | -1.024472 | -1.3626 |
| 2 | 6 | 0 | 5.819536 | -0.206514 | -0.75026 |
| 3 | 6 | 0 | 5.317098 | 1.202257 | -0.44131 |
| 4 | 6 | 0 | 3.922987 | 1.256557 | 0.153007 |
| 5 | 6 | 0 | 3.083681 | 0.19675 | 0.175112 |
| 6 | 6 | 0 | 3.471717 | -1.179755 | -0.41308 |
| 7 | 6 | 0 | 2.333864 | -1.802229 | -1.25525 |
| 8 | 6 | 0 | 3.838058 | -2.171508 | 0.722559 |
| 9 | 6 | 0 | 3.592945 | 2.631914 | 0.707636 |
| 10 | 6 | 0 | 1.712184 | 0.388537 | 0.811588 |
| 11 | 6 | 0 | 0.537976 | 0.78662 | -0.13657 |
| 12 | 6 | 0 | -0.59139 | -0.05655 | 0.385798 |
| 13 | 6 | 0 | -0.07387 | -1.020243 | 1.377147 |
| 14 | 8 | 0 | 1.265156 | -0.801506 | 1.525692 |
| 15 | 8 | 0 | 0.331698 | 2.19059 | -0.01023 |
| 16 | 8 | 0 | -0.66258 | -1.891107 | 1.974297 |
| 17 | 6 | 0 | -1.89063 | 0.007789 | 0.081234 |
| 18 | 8 | 0 | -2.33663 | 0.90286 | -0.84812 |
| 19 | 6 | 0 | -3.73352 | 0.956552 | -1.10001 |
| 20 | 6 | 0 | -4.36785 | -0.357448 | -1.48102 |
| 21 | 6 | 0 | -5.38685 | -0.622162 | -0.65771 |
| 22 | 6 | 0 | -5.46374 | 0.497847 | 0.332552 |
| 23 | 8 | 0 | -4.44464 | 1.392663 | 0.052746 |
| 24 | 8 | 0 | -6.24548 | 0.662922 | 1.228395 |
| 25 | 6 | 0 | -6.34475 | -1.764264 | -0.6002 |
| 26 | 1 | 0 | 4.355104 | -0.531443 | -2.28858 |
| 27 | 1 | 0 | 5.036905 | -2.02474 | -1.64808 |
| 28 | 1 | 0 | 6.677721 | -0.162614 | -1.43216 |
| 29 | 1 | 0 | 6.177051 | -0.686719 | 0.168681 |
| 30 | 1 | 0 | 5.318138 | 1.810991 | -1.36047 |
| 31 | 1 | 0 | 6.007286 | 1.718365 | 0.24205 |
| 32 | 1 | 0 | 1.447098 | -2.035534 | -0.66139 |
| 33 | 1 | 0 | 2.04073 | -1.145041 | -2.08293 |
| 34 | 1 | 0 | 2.68296 | -2.74434 | -1.69545 |
| 35 | 1 | 0 | 2.989666 | -2.350423 | 1.384719 |
| 36 | 1 | 0 | 4.149919 | -3.132329 | 0.292482 |
| 37 | 1 | 0 | 4.662605 | -1.791772 | 1.334531 |
| 38 | 1 | 0 | 2.536236 | 2.894562 | 0.649453 |
| 39 | 1 | 0 | 3.916391 | 2.717555 | 1.755618 |
| 40 | 1 | 0 | 4.148185 | 3.396019 | 0.150368 |
| 41 | 1 | 0 | 1.763084 | 1.175347 | 1.566482 |
| 42 | 1 | 0 | 0.775193 | 0.537318 | -1.17935 |
| 43 | 1 | 0 | -0.46267 | 2.408993 | -0.52377 |
| 44 | 1 | 0 | -2.62367 | -0.634535 | 0.560001 |
| 45 | 1 | 0 | -3.82475 | 1.722743 | -1.87477 |
| 46 | 1 | 0 | -4.0125 | -0.946642 | -2.31894 |
| 47 | 1 | 0 | -7.37642 | -1.398496 | -0.65571 |
| 48 | 1 | 0 | -6.17368 | -2.474565 | -1.41289 |
| 49 | 1 | 0 | -6.25176 | -2.292151 | 0.355969 |

Conformer 2h

| Center Number | Atomic Number | Atomic Type | Coordinates (Angstroms) | | |
|---|---|---|---|---|---|
| | | | X | Y | Z |
| 1 | 6 | 0 | 5.363077 | -1.040826 | -0.12297 |
| 2 | 6 | 0 | 4.959422 | -2.085044 | 0.914294 |
| 3 | 6 | 0 | 4.101186 | -1.417658 | 1.98706 |
| 4 | 6 | 0 | 3.004403 | -0.536004 | 1.421331 |
| 5 | 6 | 0 | 3.006354 | -0.102219 | 0.142455 |
| 6 | 6 | 0 | 4.152233 | -0.403693 | -0.84725 |
| 7 | 6 | 0 | 4.661902 | 0.901814 | -1.50766 |
| 8 | 6 | 0 | 3.649201 | -1.360419 | -1.95681 |
| 9 | 6 | 0 | 1.948697 | -0.199846 | 2.450258 |
| 10 | 6 | 0 | 1.860811 | 0.711143 | -0.43709 |
| 11 | 6 | 0 | 0.438495 | 0.062235 | -0.45503 |
| 12 | 6 | 0 | -0.43248 | 1.251123 | -0.18116 |
| 13 | 6 | 0 | 0.404517 | 2.392368 | 0.248551 |
| 14 | 8 | 0 | 1.715783 | 2.002649 | 0.226872 |
| 15 | 8 | 0 | 0.149478 | -0.527243 | -1.72077 |
| 16 | 8 | 0 | 0.061393 | 3.497148 | 0.600385 |
| 17 | 6 | 0 | -1.76377 | 1.379856 | -0.24698 |
| 18 | 8 | 0 | -2.57825 | 0.363211 | -0.61629 |
| 19 | 6 | 0 | -3.96662 | 0.623044 | -0.56309 |
| 20 | 6 | 0 | -4.70628 | -0.457171 | -1.30222 |
| 21 | 6 | 0 | -5.53254 | -1.087463 | -0.46388 |
| 22 | 6 | 0 | -5.37408 | -0.456782 | 0.883559 |
| 23 | 8 | 0 | -4.44146 | 0.557671 | 0.778621 |
| 24 | 8 | 0 | -5.93335 | -0.723678 | 1.912881 |
| 25 | 6 | 0 | -6.47713 | -2.223322 | -0.67291 |
| 26 | 1 | 0 | 5.928445 | -0.2473 | 0.386067 |
| 27 | 1 | 0 | 6.03878 | -1.470472 | -0.87388 |
| 28 | 1 | 0 | 5.84703 | -2.544315 | 1.366045 |
| 29 | 1 | 0 | 4.394194 | -2.897709 | 0.439023 |
| 30 | 1 | 0 | 4.736565 | -0.808039 | 2.649785 |
| 31 | 1 | 0 | 3.64307 | -2.172921 | 2.641664 |
| 32 | 1 | 0 | 3.933875 | 1.350262 | -2.19097 |
| 33 | 1 | 0 | 4.918071 | 1.650632 | -0.75034 |
| 34 | 1 | 0 | 5.563905 | 0.687714 | -2.09398 |
| 35 | 1 | 0 | 2.757418 | -0.967884 | -2.45918 |
| 36 | 1 | 0 | 4.42206 | -1.502125 | -2.72241 |
| 37 | 1 | 0 | 3.396262 | -2.346193 | -1.54868 |
| 38 | 1 | 0 | 1.24782 | 0.572284 | 2.138825 |
| 39 | 1 | 0 | 1.380783 | -1.097046 | 2.734934 |
| 40 | 1 | 0 | 2.434253 | 0.157651 | 3.368395 |
| 41 | 1 | 0 | 2.065704 | 0.93958 | -1.48495 |
| 42 | 1 | 0 | 0.336151 | -0.692925 | 0.334315 |
| 43 | 1 | 0 | 0.49717 | -1.432052 | -1.70505 |
| 44 | 1 | 0 | -2.23513 | 2.330941 | -0.0025 |
| 45 | 1 | 0 | -4.1644 | 1.636132 | -0.93554 |
| 46 | 1 | 0 | -4.53484 | -0.653019 | -2.35411 |
| 47 | 1 | 0 | -7.49431 | -1.93243 | -0.38627 |
| 48 | 1 | 0 | -6.48292 | -2.555332 | -1.71428 |
| 49 | 1 | 0 | -6.20312 | -3.070323 | -0.03332 |

**Supplementary Data 2.18. Cartesian coordinates of the conformers 2a−2i of 4*R**,5*S**,2'*R** isomer (continued).**

Conformer 2i

| Center Number | Atomic Number | Atomic Type | Coordinates (Angstroms) | | |
|---|---|---|---|---|---|
| | | | X | Y | Z |
| 1 | 6 | 0 | 4.291343 | -0.4566 | 2.049171 |
| 2 | 6 | 0 | 5.426915 | -1.126718 | 1.281427 |
| 3 | 6 | 0 | 4.840126 | -2.010254 | 0.182823 |
| 4 | 6 | 0 | 3.678483 | -1.386854 | -0.56935 |
| 5 | 6 | 0 | 3.026975 | -0.279256 | -0.14497 |
| 6 | 6 | 0 | 3.415909 | 0.458968 | 1.157693 |
| 7 | 6 | 0 | 2.181979 | 0.848218 | 2.004755 |
| 8 | 6 | 0 | 4.207302 | 1.751053 | 0.823077 |
| 9 | 6 | 0 | 3.35999 | -2.164897 | -1.83331 |
| 10 | 6 | 0 | 1.870143 | 0.245203 | -0.98566 |
| 11 | 6 | 0 | 0.438818 | -0.281032 | -0.62213 |
| 12 | 6 | 0 | -0.40116 | 0.936526 | -0.86011 |
| 13 | 6 | 0 | 0.473821 | 2.112024 | -1.03806 |
| 14 | 8 | 0 | 1.776663 | 1.696442 | -0.97136 |
| 15 | 8 | 0 | 0.04846 | -1.368913 | -1.45753 |
| 16 | 8 | 0 | 0.173967 | 3.272397 | -1.19475 |
| 17 | 6 | 0 | -1.73272 | 1.07523 | -0.89451 |
| 18 | 8 | 0 | -2.57972 | 0.034017 | -0.71846 |
| 19 | 6 | 0 | -3.95893 | 0.345417 | -0.70268 |
| 20 | 6 | 0 | -4.75875 | -0.92125 | -0.82962 |
| 21 | 6 | 0 | -5.52599 | -1.07582 | 0.252157 |
| 22 | 6 | 0 | -5.26489 | 0.08521 | 1.15902 |
| 23 | 8 | 0 | -4.3349 | 0.907392 | 0.550943 |
| 24 | 8 | 0 | -5.74927 | 0.330516 | 2.230655 |
| 25 | 6 | 0 | -6.49172 | -2.146498 | 0.63567 |
| 26 | 1 | 0 | 3.656424 | -1.23751 | 2.493401 |
| 27 | 1 | 0 | 4.682628 | 0.139619 | 2.883485 |
| 28 | 1 | 0 | 6.051371 | -1.725051 | 1.956135 |
| 29 | 1 | 0 | 6.084061 | -0.367745 | 0.840049 |
| 30 | 1 | 0 | 4.495819 | -2.964384 | 0.615065 |
| 31 | 1 | 0 | 5.61648 | -2.291199 | -0.54375 |
| 32 | 1 | 0 | 1.529919 | 1.562441 | 1.497613 |
| 33 | 1 | 0 | 1.590888 | -0.032454 | 2.284436 |
| 34 | 1 | 0 | 2.517456 | 1.321375 | 2.935538 |
| 35 | 1 | 0 | 3.604714 | 2.445936 | 0.236505 |
| 36 | 1 | 0 | 4.508338 | 2.253112 | 1.751641 |
| 37 | 1 | 0 | 5.113325 | 1.528941 | 0.249837 |
| 38 | 1 | 0 | 2.377876 | -1.966652 | -2.2629 |
| 39 | 1 | 0 | 4.117924 | -1.968953 | -2.60544 |
| 40 | 1 | 0 | 3.412761 | -3.242018 | -1.62449 |
| 41 | 1 | 0 | 2.02349 | -0.021739 | -2.03295 |
| 42 | 1 | 0 | 0.392116 | -0.589545 | 0.429505 |
| 43 | 1 | 0 | 0.332695 | -2.186389 | -1.02144 |
| 44 | 1 | 0 | -2.17776 | 2.053178 | -1.07352 |
| 45 | 1 | 0 | -4.17465 | 1.090939 | -1.47844 |
| 46 | 1 | 0 | -4.66652 | -1.57417 | -1.6895 |
| 47 | 1 | 0 | -7.48241 | -1.71765 | 0.825474 |
| 48 | 1 | 0 | -6.57788 | -2.908508 | -0.14318 |
| 49 | 1 | 0 | -6.17666 | -2.628401 | 1.56846 |

**Supplementary Data 2.19. Energy analysis and Boltzmann weights of low-energy conformers of 4*S**,5*S**,2'*R** isomer optimized in the gas phase at the B3LYP/6-31G(d) level for DP4+ analysis.**

| Conformer | Total energy (au) | ΔE (kJ/mol) | Gibbs free energy (au) | ΔG (kJ/mol) | Boltzmann weights |
|---|---|---|---|---|---|
| 3a | -1189.782040 | 0.00 | -1189.430462 | 0.00 | 0.405 |
| 3b | -1189.782008 | 0.08 | -1189.430106 | 0.93 | 0.278 |
| 3c | -1189.780331 | 4.49 | -1189.429753 | 1.86 | 0.191 |
| 3d | -1189.783282 | -3.26 | -1189.429366 | 2.88 | 0.127 |

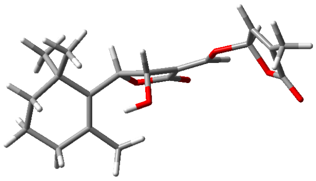

3a (40.5%)

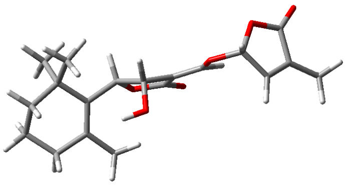

3b (27.8%)

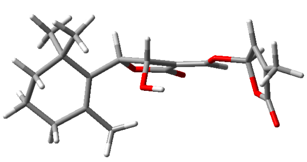

3c (19.1%)

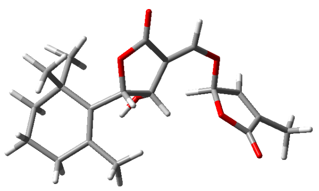

3d (12.7%)

**Supplementary Data 2.20. Low-energy conformers of 4*S**,5*S**,2'*R** isomer optimized in the gas phase at the B3LYP/6-31G(d) level for DP4+ analysis.**

**Supplementary Data 2.21. Cartesian coordinates of the conformers 3a−3d of 4*S**,5*S**,2'*R** isomer.**

Conformer 3a

| Center Number | Atomic Number | Atomic Type | Coordinates (Angstroms) X | Y | Z |
|---|---|---|---|---|---|
| 1 | 6 | 0 | -4.81258 | -1.847832 | -0.32009 |
| 2 | 6 | 0 | -5.57095 | -0.818047 | -1.15313 |
| 3 | 6 | 0 | -4.57994 | -0.005975 | -1.98507 |
| 4 | 6 | 0 | -3.32907 | 0.419987 | -1.23742 |
| 5 | 6 | 0 | -2.99329 | -0.095184 | -0.03079 |
| 6 | 6 | 0 | -3.81796 | -1.197918 | 0.670215 |
| 7 | 6 | 0 | -4.5963 | -0.596558 | 1.867398 |
| 8 | 6 | 0 | -2.90244 | -2.328634 | 1.199761 |
| 9 | 6 | 0 | -2.55722 | 1.496388 | -1.97505 |
| 10 | 6 | 0 | -1.80275 | 0.406619 | 0.76982 |
| 11 | 6 | 0 | -0.39349 | -0.157533 | 0.377746 |
| 12 | 6 | 0 | 0.471388 | 1.030109 | 0.676296 |
| 13 | 6 | 0 | -0.37208 | 2.23766 | 0.790654 |
| 14 | 8 | 0 | -1.68471 | 1.853323 | 0.729189 |
| 15 | 8 | 0 | -0.26511 | -0.511448 | -0.99334 |
| 16 | 8 | 0 | -0.0413 | 3.393159 | 0.921762 |
| 17 | 6 | 0 | 1.800014 | 1.107666 | 0.817557 |
| 18 | 8 | 0 | 2.598261 | 0.014724 | 0.755116 |
| 19 | 6 | 0 | 3.989564 | 0.249654 | 0.865904 |
| 20 | 6 | 0 | 4.705157 | -1.065788 | 1.000053 |
| 21 | 6 | 0 | 5.547926 | -1.221825 | -0.02359 |
| 22 | 6 | 0 | 5.430376 | -0.009509 | -0.89279 |
| 23 | 8 | 0 | 4.500861 | 0.840709 | -0.323 |
| 24 | 8 | 0 | 6.019212 | 0.253344 | -1.90596 |
| 25 | 6 | 0 | 6.477678 | -2.332809 | -0.37966 |
| 26 | 1 | 0 | -5.50551 | -2.486914 | 0.242252 |
| 27 | 1 | 0 | -4.25788 | -2.511919 | -0.99912 |
| 28 | 1 | 0 | -6.14238 | -0.148808 | -0.49799 |
| 29 | 1 | 0 | -6.30083 | -1.310984 | -1.80651 |
| 30 | 1 | 0 | -5.06239 | 0.895856 | -2.38747 |
| 31 | 1 | 0 | -4.26957 | -0.582599 | -2.87142 |
| 32 | 1 | 0 | -5.30934 | 0.166955 | 1.542073 |
| 33 | 1 | 0 | -5.15241 | -1.383603 | 2.391917 |
| 34 | 1 | 0 | -3.92789 | -0.126565 | 2.596989 |
| 35 | 1 | 0 | -2.23407 | -1.995803 | 2.001245 |
| 36 | 1 | 0 | -3.51612 | -3.138998 | 1.611283 |
| 37 | 1 | 0 | -2.28729 | -2.753841 | 0.39742 |
| 38 | 1 | 0 | -2.91266 | 2.492479 | -1.68425 |
| 39 | 1 | 0 | -1.4834 | 1.454777 | -1.80002 |
| 40 | 1 | 0 | -2.72559 | 1.39359 | -3.05403 |
| 41 | 1 | 0 | -1.95765 | 0.157816 | 1.824528 |
| 42 | 1 | 0 | -0.15432 | -1.023905 | 1.009157 |
| 43 | 1 | 0 | -1.11919 | -0.894971 | -1.26292 |
| 44 | 1 | 0 | 2.282574 | 2.066291 | 1.002212 |
| 45 | 1 | 0 | 4.177003 | 0.949964 | 1.690004 |
| 46 | 1 | 0 | 4.507387 | -1.748069 | 1.818369 |
| 47 | 1 | 0 | 6.203555 | -2.762012 | -1.35037 |
| 48 | 1 | 0 | 7.501778 | -1.956166 | -0.48288 |
| 49 | 1 | 0 | 6.464203 | -3.125886 | 0.372387 |

Conformer 3b

| Center Number | Atomic Number | Atomic Type | Coordinates (Angstroms) X | Y | Z |
|---|---|---|---|---|---|
| 1 | 6 | 0 | -5.26943 | -0.947923 | -0.61506 |
| 2 | 6 | 0 | -5.86987 | 0.438594 | -0.4002 |
| 3 | 6 | 0 | -4.86297 | 1.502366 | -0.83391 |
| 4 | 6 | 0 | -3.43464 | 1.235651 | -0.39314 |
| 5 | 6 | 0 | -3.03842 | 0.039253 | 0.102326 |
| 6 | 6 | 0 | -3.98502 | -1.176877 | 0.215922 |
| 7 | 6 | 0 | -4.36307 | -1.416234 | 1.699429 |
| 8 | 6 | 0 | -3.31464 | -2.460554 | -0.33144 |
| 9 | 6 | 0 | -2.55324 | 2.461367 | -0.53204 |
| 10 | 6 | 0 | -1.63941 | -0.199447 | 0.646275 |
| 11 | 6 | 0 | -0.51197 | -0.509174 | -0.39623 |
| 12 | 6 | 0 | 0.670454 | 0.103004 | 0.290944 |
| 13 | 6 | 0 | 0.201275 | 1.040144 | 1.331769 |
| 14 | 8 | 0 | -1.1586 | 0.920648 | 1.436666 |
| 15 | 8 | 0 | -0.70144 | 0.111629 | -1.6635 |
| 16 | 8 | 0 | 0.838348 | 1.811427 | 2.011257 |
| 17 | 6 | 0 | 1.977931 | -0.081983 | 0.0719 |
| 18 | 8 | 0 | 2.427605 | -0.941201 | -0.87351 |
| 19 | 6 | 0 | 3.829882 | -1.046885 | -1.0564 |
| 20 | 6 | 0 | 4.529433 | 0.247222 | -1.38811 |
| 21 | 6 | 0 | 5.517759 | 0.463772 | -0.51499 |
| 22 | 6 | 0 | 5.509713 | -0.671547 | 0.459649 |
| 23 | 8 | 0 | 4.472525 | -1.523623 | 0.122958 |
| 24 | 8 | 0 | 6.244588 | -0.877699 | 1.387099 |
| 25 | 6 | 0 | 6.509942 | 1.571616 | -0.39512 |
| 26 | 1 | 0 | -5.99507 | -1.733749 | -0.36883 |
| 27 | 1 | 0 | -5.03062 | -1.067859 | -1.68205 |
| 28 | 1 | 0 | -6.12702 | 0.579272 | 0.656966 |
| 29 | 1 | 0 | -6.80395 | 0.547462 | -0.9644 |
| 30 | 1 | 0 | -5.15953 | 2.490846 | -0.45568 |
| 31 | 1 | 0 | -4.87002 | 1.603354 | -1.93136 |
| 32 | 1 | 0 | -4.89931 | -0.561259 | 2.122226 |
| 33 | 1 | 0 | -5.00496 | -2.301536 | 1.788555 |
| 34 | 1 | 0 | -3.48074 | -1.587956 | 2.325413 |
| 35 | 1 | 0 | -2.45479 | -2.781054 | 0.266503 |
| 36 | 1 | 0 | -4.03579 | -3.286641 | -0.32217 |
| 37 | 1 | 0 | -2.97896 | -2.326038 | -1.36695 |
| 38 | 1 | 0 | -2.59429 | 3.069775 | 0.379762 |
| 39 | 1 | 0 | -1.51053 | 2.222392 | -0.73386 |
| 40 | 1 | 0 | -2.92154 | 3.085589 | -1.3556 |
| 41 | 1 | 0 | -1.6696 | -1.049681 | 1.334979 |
| 42 | 1 | 0 | -0.41157 | -1.59484 | -0.52459 |
| 43 | 1 | 0 | -1.65527 | 0.062611 | -1.85593 |
| 44 | 1 | 0 | 2.715968 | 0.451126 | 0.665252 |
| 45 | 1 | 0 | 3.929817 | -1.80668 | -1.83604 |
| 46 | 1 | 0 | 4.230378 | 0.861002 | -2.23026 |
| 47 | 1 | 0 | 6.389199 | 2.090303 | 0.563001 |
| 48 | 1 | 0 | 7.530446 | 1.171996 | -0.40768 |
| 49 | 1 | 0 | 6.401331 | 2.297912 | -1.20449 |

**Supplementary Data 2.21. Cartesian coordinates of the conformers 3a−3d of 4*S**,5*S**,2'*R** isomer (continued).**

Conformer 3c

| Center Number | Atomic Number | Atomic Type | Coordinates (Angstroms) | | |
|---|---|---|---|---|---|
| | | | X | Y | Z |
| 1 | 6 | 0 | -4.69908 | -1.929764 | -0.18879 |
| 2 | 6 | 0 | -5.40103 | -1.051643 | -1.22174 |
| 3 | 6 | 0 | -4.3536 | -0.353014 | -2.08676 |
| 4 | 6 | 0 | -3.17795 | 0.21004 | -1.31177 |
| 5 | 6 | 0 | -2.9483 | -0.100653 | -0.01986 |
| 6 | 6 | 0 | -3.79967 | -1.115685 | 0.770623 |
| 7 | 6 | 0 | -4.68329 | -0.390573 | 1.815913 |
| 8 | 6 | 0 | -2.88867 | -2.127962 | 1.508183 |
| 9 | 6 | 0 | -2.32233 | 1.139821 | -2.14634 |
| 10 | 6 | 0 | -1.83693 | 0.544411 | 0.789484 |
| 11 | 6 | 0 | -0.38829 | -0.025724 | 0.641246 |
| 12 | 6 | 0 | 0.432298 | 1.234232 | 0.65094 |
| 13 | 6 | 0 | -0.45466 | 2.412981 | 0.541164 |
| 14 | 8 | 0 | -1.74351 | 1.978176 | 0.533742 |
| 15 | 8 | 0 | -0.25705 | -0.780581 | -0.56026 |
| 16 | 8 | 0 | -0.15359 | 3.582014 | 0.461825 |
| 17 | 6 | 0 | 1.761677 | 1.363647 | 0.697271 |
| 18 | 8 | 0 | 2.570851 | 0.271223 | 0.799242 |
| 19 | 6 | 0 | 3.963978 | 0.507654 | 0.708058 |
| 20 | 6 | 0 | 4.712931 | -0.71125 | 1.170579 |
| 21 | 6 | 0 | 5.465915 | -1.178965 | 0.17178 |
| 22 | 6 | 0 | 5.244344 | -0.291316 | -1.01263 |
| 23 | 8 | 0 | 4.350203 | 0.699516 | -0.6477 |
| 24 | 8 | 0 | 5.72907 | -0.358862 | -2.109 |
| 25 | 6 | 0 | 6.386738 | -2.34986 | 0.090804 |
| 26 | 1 | 0 | -5.42644 | -2.49971 | 0.404303 |
| 27 | 1 | 0 | -4.07823 | -2.66706 | -0.71745 |
| 28 | 1 | 0 | -6.02844 | -0.304217 | -0.71971 |
| 29 | 1 | 0 | -6.07273 | -1.652035 | -1.84757 |
| 30 | 1 | 0 | -4.81228 | 0.46157 | -2.66588 |
| 31 | 1 | 0 | -3.95992 | -1.054953 | -2.8399 |
| 32 | 1 | 0 | -5.40284 | 0.281568 | 1.338129 |
| 33 | 1 | 0 | -5.24246 | -1.12115 | 2.414248 |
| 34 | 1 | 0 | -4.08849 | 0.212567 | 2.511191 |
| 35 | 1 | 0 | -2.31849 | -1.666464 | 2.323099 |
| 36 | 1 | 0 | -3.49934 | -2.920922 | 1.95732 |
| 37 | 1 | 0 | -2.17921 | -2.592259 | 0.814554 |
| 38 | 1 | 0 | -2.65648 | 2.180582 | -2.05388 |
| 39 | 1 | 0 | -1.26643 | 1.092617 | -1.88112 |
| 40 | 1 | 0 | -2.40737 | 0.864166 | -3.20464 |
| 41 | 1 | 0 | -2.09167 | 0.471527 | 1.850797 |
| 42 | 1 | 0 | -0.16063 | -0.66835 | 1.502732 |
| 43 | 1 | 0 | 0.689824 | -0.968196 | -0.66709 |
| 44 | 1 | 0 | 2.243348 | 2.338982 | 0.655612 |
| 45 | 1 | 0 | 4.212729 | 1.423068 | 1.259308 |
| 46 | 1 | 0 | 4.605578 | -1.106377 | 2.173949 |
| 47 | 1 | 0 | 6.047115 | -3.049659 | -0.68152 |
| 48 | 1 | 0 | 7.39165 | -2.02543 | -0.20281 |
| 49 | 1 | 0 | 6.448863 | -2.880016 | 1.044528 |

Conformer 3d

| Center Number | Atomic Number | Atomic Type | Coordinates (Angstroms) | | |
|---|---|---|---|---|---|
| | | | X | Y | Z |
| 1 | 6 | 0 | 4.321621 | -0.845753 | 1.402511 |
| 2 | 6 | 0 | 4.775574 | -2.046456 | 0.578486 |
| 3 | 6 | 0 | 3.554393 | -2.747874 | -0.01119 |
| 4 | 6 | 0 | 2.508605 | -1.800134 | -0.57129 |
| 5 | 6 | 0 | 2.483827 | -0.471385 | -0.3058 |
| 6 | 6 | 0 | 3.553708 | 0.217861 | 0.576954 |
| 7 | 6 | 0 | 2.958769 | 1.215763 | 1.601785 |
| 8 | 6 | 0 | 4.551102 | 0.982952 | -0.3319 |
| 9 | 6 | 0 | 1.523895 | -2.527979 | -1.46595 |
| 10 | 6 | 0 | 1.371564 | 0.357927 | -0.95528 |
| 11 | 6 | 0 | -0.02972 | 0.243087 | -0.2588 |
| 12 | 6 | 0 | -0.5341 | 1.65127 | -0.34229 |
| 13 | 6 | 0 | 0.579658 | 2.537679 | -0.75025 |
| 14 | 8 | 0 | 1.679835 | 1.767319 | -1.01487 |
| 15 | 8 | 0 | 0.04039 | -0.17187 | 1.109782 |
| 16 | 8 | 0 | 0.602983 | 3.74114 | -0.85487 |
| 17 | 6 | 0 | -1.73131 | 2.223211 | -0.11941 |
| 18 | 8 | 0 | -2.9138 | 1.709802 | 0.279836 |
| 19 | 6 | 0 | -3.02156 | 0.339898 | 0.641779 |
| 20 | 6 | 0 | -4.34484 | 0.127864 | 1.323146 |
| 21 | 6 | 0 | -5.08029 | -0.741201 | 0.625041 |
| 22 | 6 | 0 | -4.28128 | -1.146291 | -0.57074 |
| 23 | 8 | 0 | -3.06885 | -0.483528 | -0.52396 |
| 24 | 8 | 0 | -4.56518 | -1.917128 | -1.44828 |
| 25 | 6 | 0 | -6.44904 | -1.289085 | 0.85587 |
| 26 | 1 | 0 | 3.673258 | -1.199353 | 2.218283 |
| 27 | 1 | 0 | 5.179786 | -0.35619 | 1.879872 |
| 28 | 1 | 0 | 5.352498 | -2.74523 | 1.196448 |
| 29 | 1 | 0 | 5.442832 | -1.720428 | -0.22867 |
| 30 | 1 | 0 | 3.076068 | -3.377457 | 0.756858 |
| 31 | 1 | 0 | 3.855261 | -3.446411 | -0.80486 |
| 32 | 1 | 0 | 2.133226 | 0.772984 | 2.166458 |
| 33 | 1 | 0 | 3.743666 | 1.500437 | 2.313272 |
| 34 | 1 | 0 | 2.59757 | 2.130406 | 1.130582 |
| 35 | 1 | 0 | 4.041643 | 1.754119 | -0.91291 |
| 36 | 1 | 0 | 5.315621 | 1.471202 | 0.285814 |
| 37 | 1 | 0 | 5.059826 | 0.311631 | -1.03192 |
| 38 | 1 | 0 | 1.126647 | -3.412575 | -0.95017 |
| 39 | 1 | 0 | 0.669133 | -1.939018 | -1.79979 |
| 40 | 1 | 0 | 2.038964 | -2.903638 | -2.36109 |
| 41 | 1 | 0 | 1.250539 | 0.046337 | -1.99736 |
| 42 | 1 | 0 | -0.66159 | -0.457271 | -0.81644 |
| 43 | 1 | 0 | 0.631089 | -0.947134 | 1.130504 |
| 44 | 1 | 0 | -1.82311 | 3.295658 | -0.26945 |
| 45 | 1 | 0 | -2.14791 | 0.043846 | 1.230552 |
| 46 | 1 | 0 | -4.60594 | 0.642135 | 2.24065 |
| 47 | 1 | 0 | -7.11114 | -1.026548 | 0.022618 |
| 48 | 1 | 0 | -6.42131 | -2.38395 | 0.899896 |
| 49 | 1 | 0 | -6.88318 | -0.907564 | 1.783615 |

**Supplementary Data 2.22. Energy analysis and Boltzmann weights of low-energy conformers of 4*R**,5*R**,2'*R** isomer optimized in the gas phase at the B3LYP/6-31G(d) level for DP4+ analysis.**

| Conformer | Total energy (au) | ΔE (kJ/mol) | Gibbs free energy (au) | ΔG (kJ/mol) | Boltzmann weights |
|---|---|---|---|---|---|
| 4a | -1189.783127 | 0.00 | -1189.431560 | 0.00 | 0.704 |
| 4b | -1189.780725 | 6.31 | -1189.429818 | 4.57 | 0.111 |
| 4c | -1189.780275 | 7.49 | -1189.429382 | 5.72 | 0.070 |
| 4d | -1189.780622 | 6.58 | -1189.429372 | 5.74 | 0.069 |
| 4e | -1189.780788 | 6.14 | -1189.428975 | 6.79 | 0.045 |

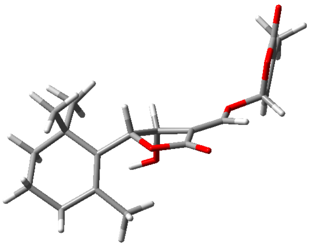

4a (70.4%)

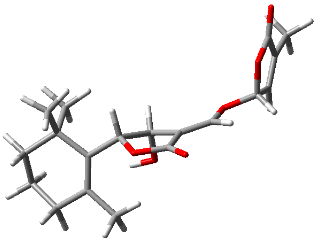

4b (11.1%)

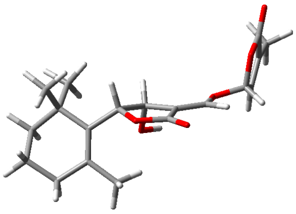

4c (7.0%)

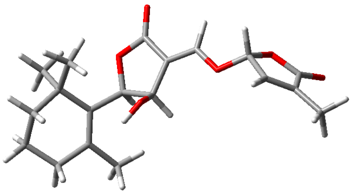

4d (6.9%)

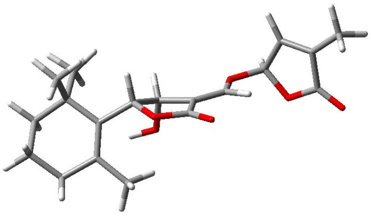

4e (4.5%)

**Supplementary Data 2.23. Low-energy conformers of 4*R**,5*R**,2'*R** isomer optimized in the gas phase at the B3LYP/6-31G(d) level for DP4+ analysis.**

**Supplementary Data 2.24. Cartesian coordinates of the conformers 4a−4e of 4*R**,5*R**,2'*R** isomer.**

Conformer 4a

| Center Number | Atomic Number | Atomic Type | Coordinates (Angstroms) X | Y | Z |
|---|---|---|---|---|---|
| 1 | 6 | 0 | -4.69525 | -2.02909 | 0.197524 |
| 2 | 6 | 0 | -5.7142 | -0.941516 | 0.526534 |
| 3 | 6 | 0 | -5.03691 | 0.162767 | 1.336205 |
| 4 | 6 | 0 | -3.66131 | 0.561584 | 0.831959 |
| 5 | 6 | 0 | -2.98179 | -0.171375 | -0.0819 |
| 6 | 6 | 0 | -3.5162 | -1.503503 | -0.65439 |
| 7 | 6 | 0 | -2.42174 | -2.598393 | -0.63712 |
| 8 | 6 | 0 | -3.98316 | -1.300899 | -2.11788 |
| 9 | 6 | 0 | -3.1875 | 1.870752 | 1.432152 |
| 10 | 6 | 0 | -1.65496 | 0.279217 | -0.67071 |
| 11 | 6 | 0 | -0.36824 | 0.002755 | 0.179743 |
| 12 | 6 | 0 | 0.474264 | 1.179331 | -0.20936 |
| 13 | 6 | 0 | -0.3834 | 2.21826 | -0.81592 |
| 14 | 8 | 0 | -1.6412 | 1.7005 | -0.97033 |
| 15 | 8 | 0 | -0.58431 | 0.01325 | 1.586607 |
| 16 | 8 | 0 | -0.10216 | 3.347278 | -1.14461 |
| 17 | 6 | 0 | 1.790751 | 1.37108 | -0.06759 |
| 18 | 8 | 0 | 2.597479 | 0.443824 | 0.502667 |
| 19 | 6 | 0 | 3.984066 | 0.719295 | 0.479876 |
| 20 | 6 | 0 | 4.697421 | -0.233509 | 1.39821 |
| 21 | 6 | 0 | 5.571343 | -0.959939 | 0.697175 |
| 22 | 6 | 0 | 5.474053 | -0.526488 | -0.73141 |
| 23 | 8 | 0 | 4.526168 | 0.476419 | -0.81467 |
| 24 | 8 | 0 | 6.086862 | -0.92386 | -1.68525 |
| 25 | 6 | 0 | 6.515789 | -2.039151 | 1.108694 |
| 26 | 1 | 0 | -4.30076 | -2.439773 | 1.138587 |
| 27 | 1 | 0 | -5.16932 | -2.865522 | -0.33197 |
| 28 | 1 | 0 | -6.55902 | -1.359522 | 1.08705 |
| 29 | 1 | 0 | -6.13111 | -0.522424 | -0.39763 |
| 30 | 1 | 0 | -4.93935 | -0.147272 | 2.389278 |
| 31 | 1 | 0 | -5.66667 | 1.06321 | 1.362308 |
| 32 | 1 | 0 | -2.01715 | -2.743518 | 0.372001 |
| 33 | 1 | 0 | -2.85002 | -3.554033 | -0.96251 |
| 34 | 1 | 0 | -1.58668 | -2.377388 | -1.31048 |
| 35 | 1 | 0 | -3.17522 | -0.932491 | -2.75924 |
| 36 | 1 | 0 | -4.32843 | -2.252676 | -2.54051 |
| 37 | 1 | 0 | -4.8037 | -0.580022 | -2.18355 |
| 38 | 1 | 0 | -3.62846 | 1.997436 | 2.428598 |
| 39 | 1 | 0 | -2.10544 | 1.931533 | 1.535393 |
| 40 | 1 | 0 | -3.52033 | 2.717652 | 0.819685 |
| 41 | 1 | 0 | -1.50785 | -0.222144 | -1.63248 |
| 42 | 1 | 0 | 0.078356 | -0.953945 | -0.12124 |
| 43 | 1 | 0 | -1.45146 | -0.404981 | 1.736675 |
| 44 | 1 | 0 | 2.258016 | 2.293447 | -0.40986 |
| 45 | 1 | 0 | 4.150165 | 1.778106 | 0.715491 |
| 46 | 1 | 0 | 4.475614 | -0.283931 | 2.457671 |
| 47 | 1 | 0 | 7.542678 | -1.775152 | 0.830826 |
| 48 | 1 | 0 | 6.474585 | -2.220534 | 2.185749 |
| 49 | 1 | 0 | 6.28064 | -2.972402 | 0.58399 |

Conformer 4b

| Center Number | Atomic Number | Atomic Type | Coordinates (Angstroms) X | Y | Z |
|---|---|---|---|---|---|
| 1 | 6 | 0 | 5.032281 | -1.640062 | 0.420438 |
| 2 | 6 | 0 | 5.196819 | -1.534839 | -1.0914 |
| 3 | 6 | 0 | 4.91065 | -0.096764 | -1.51354 |
| 4 | 6 | 0 | 3.619203 | 0.459434 | -0.93867 |
| 5 | 6 | 0 | 3.004544 | -0.103424 | 0.130545 |
| 6 | 6 | 0 | 3.598759 | -1.307198 | 0.904261 |
| 7 | 6 | 0 | 2.695548 | -2.552647 | 0.731036 |
| 8 | 6 | 0 | 3.714776 | -0.986475 | 2.416986 |
| 9 | 6 | 0 | 3.16938 | 1.704704 | -1.67135 |
| 10 | 6 | 0 | 1.666897 | 0.381332 | 0.669305 |
| 11 | 6 | 0 | 0.394029 | 0.06214 | -0.18759 |
| 12 | 6 | 0 | -0.4721 | 1.230095 | 0.175719 |
| 13 | 6 | 0 | 0.364993 | 2.301639 | 0.754053 |
| 14 | 8 | 0 | 1.636855 | 1.816671 | 0.910523 |
| 15 | 8 | 0 | 0.608244 | 0.049897 | -1.59143 |
| 16 | 8 | 0 | 0.059828 | 3.431745 | 1.056469 |
| 17 | 6 | 0 | -1.79242 | 1.391472 | 0.03182 |
| 18 | 8 | 0 | -2.58089 | 0.4358 | -0.51625 |
| 19 | 6 | 0 | -3.97306 | 0.68235 | -0.49465 |
| 20 | 6 | 0 | -4.66826 | -0.300709 | -1.39484 |
| 21 | 6 | 0 | -5.52504 | -1.033375 | -0.67928 |
| 22 | 6 | 0 | -5.43338 | -0.573956 | 0.741556 |
| 23 | 8 | 0 | -4.50678 | 0.450065 | 0.805247 |
| 24 | 8 | 0 | -6.03531 | -0.96798 | 1.703658 |
| 25 | 6 | 0 | -6.44774 | -2.138785 | -1.07008 |
| 26 | 1 | 0 | 5.301056 | -2.640899 | 0.781819 |
| 27 | 1 | 0 | 5.736816 | -0.940438 | 0.892174 |
| 28 | 1 | 0 | 4.506221 | -2.219067 | -1.60272 |
| 29 | 1 | 0 | 6.209583 | -1.828784 | -1.39236 |
| 30 | 1 | 0 | 4.869394 | -0.01362 | -2.60828 |
| 31 | 1 | 0 | 5.743395 | 0.555563 | -1.20511 |
| 32 | 1 | 0 | 2.652736 | -2.87857 | -0.31454 |
| 33 | 1 | 0 | 3.079052 | -3.388481 | 1.328978 |
| 34 | 1 | 0 | 1.667837 | -2.363256 | 1.062195 |
| 35 | 1 | 0 | 2.747326 | -0.94004 | 2.926993 |
| 36 | 1 | 0 | 4.297275 | -1.773318 | 2.911365 |
| 37 | 1 | 0 | 4.228812 | -0.0327 | 2.578783 |
| 38 | 1 | 0 | 2.998216 | 1.483271 | -2.73268 |
| 39 | 1 | 0 | 2.272831 | 2.163669 | -1.26907 |
| 40 | 1 | 0 | 3.978242 | 2.448176 | -1.63587 |
| 41 | 1 | 0 | 1.489092 | -0.077987 | 1.644484 |
| 42 | 1 | 0 | -0.03476 | -0.896515 | 0.136559 |
| 43 | 1 | 0 | 1.430607 | -0.449998 | -1.7388 |
| 44 | 1 | 0 | -2.27788 | 2.311554 | 0.354429 |
| 45 | 1 | 0 | -4.16194 | 1.733261 | -0.74774 |
| 46 | 1 | 0 | -4.4481 | -0.364218 | -2.45394 |
| 47 | 1 | 0 | -7.47954 | -1.890672 | -0.79576 |
| 48 | 1 | 0 | -6.40387 | -2.338675 | -2.14375 |
| 49 | 1 | 0 | -6.19328 | -3.057431 | -0.52889 |

**Supplementary Data 2.24. Cartesian coordinates of the conformers 4a−4e of 4*R**,5*R**,2'*R** isomer (continued).**

Conformer 4c

| Center Number | Atomic Number | Atomic Type | Coordinates (Angstroms) X | Y | Z |
|---|---|---|---|---|---|
| 1 | 6 | 0 | 4.706793 | -1.978571 | -0.1925 |
| 2 | 6 | 0 | 5.656205 | -0.891173 | -0.68936 |
| 3 | 6 | 0 | 4.874171 | 0.118925 | -1.52713 |
| 4 | 6 | 0 | 3.534868 | 0.516142 | -0.93691 |
| 5 | 6 | 0 | 2.972279 | -0.148664 | 0.092413 |
| 6 | 6 | 0 | 3.585422 | -1.42013 | 0.714485 |
| 7 | 6 | 0 | 2.512562 | -2.529275 | 0.846456 |
| 8 | 6 | 0 | 4.160656 | -1.116606 | 2.120272 |
| 9 | 6 | 0 | 2.928638 | 1.720261 | -1.62675 |
| 10 | 6 | 0 | 1.686622 | 0.312611 | 0.756464 |
| 11 | 6 | 0 | 0.328594 | -0.062442 | 0.077826 |
| 12 | 6 | 0 | -0.46234 | 1.201747 | 0.269755 |
| 13 | 6 | 0 | 0.423539 | 2.279657 | 0.76093 |
| 14 | 8 | 0 | 1.666323 | 1.759037 | 0.947907 |
| 15 | 8 | 0 | 0.51997 | -0.416766 | -1.29045 |
| 16 | 8 | 0 | 0.158655 | 3.441623 | 0.968978 |
| 17 | 6 | 0 | -1.75675 | 1.42202 | 0.021682 |
| 18 | 8 | 0 | -2.5466 | 0.447768 | -0.51278 |
| 19 | 6 | 0 | -3.93902 | 0.707136 | -0.52866 |
| 20 | 6 | 0 | -4.62925 | -0.30058 | -1.40535 |
| 21 | 6 | 0 | -5.50398 | -1.000288 | -0.67841 |
| 22 | 6 | 0 | -5.43024 | -0.490979 | 0.727093 |
| 23 | 8 | 0 | -4.4956 | 0.527878 | 0.767788 |
| 24 | 8 | 0 | -6.04994 | -0.845172 | 1.692555 |
| 25 | 6 | 0 | -6.43251 | -2.10892 | -1.04443 |
| 26 | 1 | 0 | 4.248067 | -2.469588 | -1.06263 |
| 27 | 1 | 0 | 5.252059 | -2.757855 | 0.356073 |
| 28 | 1 | 0 | 6.469422 | -1.328453 | -1.28165 |
| 29 | 1 | 0 | 6.128709 | -0.383553 | 0.161082 |
| 30 | 1 | 0 | 4.694739 | -0.288709 | -2.53547 |
| 31 | 1 | 0 | 5.469062 | 1.02984 | -1.6877 |
| 32 | 1 | 0 | 2.012185 | -2.704369 | -0.1122 |
| 33 | 1 | 0 | 2.98345 | -3.467509 | 1.164737 |
| 34 | 1 | 0 | 1.746877 | -2.289243 | 1.593494 |
| 35 | 1 | 0 | 3.401839 | -0.70671 | 2.796275 |
| 36 | 1 | 0 | 4.545084 | -2.035541 | 2.58082 |
| 37 | 1 | 0 | 4.978241 | -0.390547 | 2.072944 |
| 38 | 1 | 0 | 3.289575 | 1.769316 | -2.66155 |
| 39 | 1 | 0 | 1.840049 | 1.68165 | -1.66244 |
| 40 | 1 | 0 | 3.225695 | 2.654448 | -1.13438 |
| 41 | 1 | 0 | 1.650909 | -0.098386 | 1.769322 |
| 42 | 1 | 0 | -0.12449 | -0.908055 | 0.612385 |
| 43 | 1 | 0 | -0.36664 | -0.549266 | -1.66349 |
| 44 | 1 | 0 | -2.21899 | 2.389907 | 0.208742 |
| 45 | 1 | 0 | -4.10872 | 1.750568 | -0.82305 |
| 46 | 1 | 0 | -4.40011 | -0.400601 | -2.45991 |
| 47 | 1 | 0 | -7.46525 | -1.840019 | -0.79444 |
| 48 | 1 | 0 | -6.37524 | -2.347397 | -2.10951 |
| 49 | 1 | 0 | -6.19653 | -3.010111 | -0.46677 |

Conformer 4d

| Center Number | Atomic Number | Atomic Type | Coordinates (Angstroms) X | Y | Z |
|---|---|---|---|---|---|
| 1 | 6 | 0 | -4.90214 | -0.460077 | 1.235425 |
| 2 | 6 | 0 | -5.54713 | -1.495792 | 0.320112 |
| 3 | 6 | 0 | -4.46495 | -2.401334 | -0.26285 |
| 4 | 6 | 0 | -3.2202 | -1.660744 | -0.71845 |
| 5 | 6 | 0 | -2.93894 | -0.382036 | -0.36934 |
| 6 | 6 | 0 | -3.8917 | 0.465028 | 0.51029 |
| 7 | 6 | 0 | -4.66335 | 1.46614 | -0.38873 |
| 8 | 6 | 0 | -3.1612 | 1.263161 | 1.619673 |
| 9 | 6 | 0 | -2.35565 | -2.529009 | -1.61185 |
| 10 | 6 | 0 | -1.64595 | 0.231099 | -0.91445 |
| 11 | 6 | 0 | -0.33688 | -0.210928 | -0.16335 |
| 12 | 6 | 0 | 0.437776 | 1.068387 | -0.14366 |
| 13 | 6 | 0 | -0.4418 | 2.189828 | -0.51957 |
| 14 | 8 | 0 | -1.65714 | 1.677975 | -0.89012 |
| 15 | 8 | 0 | -0.5559 | -0.670177 | 1.165032 |
| 16 | 8 | 0 | -0.21376 | 3.376589 | -0.52852 |
| 17 | 6 | 0 | 1.72482 | 1.279455 | 0.15722 |
| 18 | 8 | 0 | 2.553969 | 0.273558 | 0.521535 |
| 19 | 6 | 0 | 3.916233 | 0.613846 | 0.695011 |
| 20 | 6 | 0 | 4.629164 | -0.517511 | 1.381784 |
| 21 | 6 | 0 | 5.599069 | -0.980664 | 0.589512 |
| 22 | 6 | 0 | 5.571112 | -0.178486 | -0.67301 |
| 23 | 8 | 0 | 4.562956 | 0.761758 | -0.56476 |
| 24 | 8 | 0 | 6.274831 | -0.26936 | -1.64228 |
| 25 | 6 | 0 | 6.586671 | -2.083324 | 0.775935 |
| 26 | 1 | 0 | -5.66678 | 0.169756 | 1.70728 |
| 27 | 1 | 0 | -4.38291 | -0.98237 | 2.052967 |
| 28 | 1 | 0 | -6.09116 | -0.996783 | -0.49114 |
| 29 | 1 | 0 | -6.28552 | -2.092638 | 0.869322 |
| 30 | 1 | 0 | -4.85979 | -2.979351 | -1.11052 |
| 31 | 1 | 0 | -4.16587 | -3.156036 | 0.482891 |
| 32 | 1 | 0 | -5.26207 | 0.952353 | -1.14845 |
| 33 | 1 | 0 | -5.34233 | 2.069708 | 0.227038 |
| 34 | 1 | 0 | -3.97722 | 2.1439 | -0.90038 |
| 35 | 1 | 0 | -2.61576 | 2.120548 | 1.224007 |
| 36 | 1 | 0 | -3.90641 | 1.646408 | 2.327602 |
| 37 | 1 | 0 | -2.45659 | 0.635836 | 2.173102 |
| 38 | 1 | 0 | -2.89357 | -2.762565 | -2.54123 |
| 39 | 1 | 0 | -1.39097 | -2.101391 | -1.88703 |
| 40 | 1 | 0 | -2.157 | -3.493309 | -1.12457 |
| 41 | 1 | 0 | -1.5348 | -0.035507 | -1.97015 |
| 42 | 1 | 0 | 0.176916 | -0.990788 | -0.74278 |
| 43 | 1 | 0 | -1.29835 | -1.298831 | 1.115344 |
| 44 | 1 | 0 | 2.14642 | 2.282959 | 0.118044 |
| 45 | 1 | 0 | 3.988158 | 1.574465 | 1.22039 |
| 46 | 1 | 0 | 4.338019 | -0.86642 | 2.365482 |
| 47 | 1 | 0 | 7.609173 | -1.702645 | 0.67109 |
| 48 | 1 | 0 | 6.482128 | -2.555425 | 1.756144 |
| 49 | 1 | 0 | 6.456915 | -2.846855 | 0.000222 |

**Supplementary Data 2.24. Cartesian coordinates of the conformers 4a−4e of 4*R**,5*R**,2'*R** isomer (continued).**

Conformer 4e

| Center Number | Atomic Number | Atomic Type | Coordinates (Angstroms) | | |
|---|---|---|---|---|---|
| | | | X | Y | Z |
| 1 | 6 | 0 | 5.273356 | -0.962394 | -0.5909 |
| 2 | 6 | 0 | 5.875793 | 0.418916 | -0.34954 |
| 3 | 6 | 0 | 4.877282 | 1.49211 | -0.77943 |
| 4 | 6 | 0 | 3.443504 | 1.224415 | -0.35781 |
| 5 | 6 | 0 | 3.038341 | 0.022549 | 0.11693 |
| 6 | 6 | 0 | 3.978977 | -1.198778 | 0.222478 |
| 7 | 6 | 0 | 3.309386 | -2.470897 | -0.35225 |
| 8 | 6 | 0 | 4.340062 | -1.46284 | 1.705947 |
| 9 | 6 | 0 | 2.566487 | 2.453848 | -0.49055 |
| 10 | 6 | 0 | 1.632627 | -0.217319 | 0.642798 |
| 11 | 6 | 0 | 0.514035 | -0.502748 | -0.41752 |
| 12 | 6 | 0 | -0.66978 | 0.12306 | 0.256276 |
| 13 | 6 | 0 | -0.20468 | 1.030678 | 1.324635 |
| 14 | 8 | 0 | 1.151721 | 0.891017 | 1.448757 |
| 15 | 8 | 0 | 0.730453 | 0.11863 | -1.67807 |
| 16 | 8 | 0 | -0.84341 | 1.787713 | 2.018465 |
| 17 | 6 | 0 | -1.97702 | -0.024814 | 0.0075 |
| 18 | 8 | 0 | -2.43218 | -0.865077 | -0.95192 |
| 19 | 6 | 0 | -3.8369 | -0.996547 | -1.10232 |
| 20 | 6 | 0 | -4.57092 | -1.458132 | 0.132133 |
| 21 | 6 | 0 | -5.54129 | -0.589988 | 0.432792 |
| 22 | 6 | 0 | -5.48662 | 0.508947 | -0.58186 |
| 23 | 8 | 0 | -4.44081 | 0.245978 | -1.44833 |
| 24 | 8 | 0 | -6.19648 | 1.471433 | -0.69159 |
| 25 | 6 | 0 | -6.55304 | -0.572495 | 1.529087 |
| 26 | 1 | 0 | 5.045956 | -1.066021 | -1.66204 |
| 27 | 1 | 0 | 5.993673 | -1.754229 | -0.34812 |
| 28 | 1 | 0 | 6.81647 | 0.532435 | -0.90171 |
| 29 | 1 | 0 | 6.121624 | 0.543733 | 0.712344 |
| 30 | 1 | 0 | 4.896325 | 1.608166 | -1.87517 |
| 31 | 1 | 0 | 5.172891 | 2.474265 | -0.38448 |
| 32 | 1 | 0 | 2.985335 | -2.318568 | -1.38894 |
| 33 | 1 | 0 | 4.026717 | -3.300402 | -0.34812 |
| 34 | 1 | 0 | 2.441459 | -2.796515 | 0.231337 |
| 35 | 1 | 0 | 3.450219 | -1.640331 | 2.319712 |
| 36 | 1 | 0 | 4.977541 | -2.352023 | 1.788415 |
| 37 | 1 | 0 | 4.874977 | -0.616656 | 2.147615 |
| 38 | 1 | 0 | 2.94835 | 3.090582 | -1.29799 |
| 39 | 1 | 0 | 1.526701 | 2.220473 | -0.71348 |
| 40 | 1 | 0 | 2.593906 | 3.047346 | 0.431491 |
| 41 | 1 | 0 | 1.651822 | -1.079366 | 1.317276 |
| 42 | 1 | 0 | 0.400435 | -1.586987 | -0.55196 |
| 43 | 1 | 0 | 1.687645 | 0.068776 | -1.85239 |
| 44 | 1 | 0 | -2.71092 | 0.534628 | 0.58147 |
| 45 | 1 | 0 | -3.93995 | -1.679194 | -1.94998 |
| 46 | 1 | 0 | -4.30851 | -2.375159 | 0.647677 |
| 47 | 1 | 0 | -7.56464 | -0.508101 | 1.112279 |
| 48 | 1 | 0 | -6.48204 | -1.464295 | 2.156903 |
| 49 | 1 | 0 | -6.41796 | 0.31447 | 2.158936 |

**Supplementary Data 2.25. Energy analysis and Boltzmann weights of low-energy conformers of 4*S*,5*R*,2'*R** isomer optimized at the ωB97X-D/TZVP level in acetonitrile (PCM) for ECD calculation.**

| Conformer | Total energy (au) | ΔE (kJ/mol) | Gibbs free energy (au) | ΔG (kJ/mol) | Boltzmann weights |
|---|---|---|---|---|---|
| 1ae | -1189.855611 | 0.00 | -1189.502466 | 0.00 | 0.369 |
| 1be | -1189.855365 | 0.65 | -1189.501938 | 1.39 | 0.211 |
| 1ce | -1189.854973 | 1.68 | -1189.501673 | 2.08 | 0.159 |
| 1de | -1189.855115 | 1.30 | -1189.500996 | 3.86 | 0.078 |
| 1ee | -1189.854384 | 3.22 | -1189.500591 | 4.92 | 0.051 |
| 1fe | -1189.853421 | 5.75 | -1189.500457 | 5.27 | 0.044 |
| 1ge | -1189.854679 | 2.45 | -1189.500372 | 5.50 | 0.040 |
| 1he | -1189.852745 | 7.52 | -1189.499341 | 8.20 | 0.013 |
| 1ie | -1189.852204 | 8.94 | -1189.499146 | 8.72 | 0.011 |
| 1je | -1189.855205 | 1.07 | -1189.498835 | 9.53 | 0.008 |
| 1ke | -1189.855021 | 1.55 | -1189.498726 | 9.82 | 0.007 |
| 1le | -1189.854488 | 2.95 | -1189.498257 | 11.05 | 0.004 |
| 1me | -1189.850898 | 12.37 | -1189.497910 | 11.96 | 0.003 |
| 1ne | -1189.851043 | 11.99 | -1189.497721 | 12.46 | 0.002 |
| 1oe | -1189.852260 | 8.80 | -1189.496675 | 15.20 | 0.001 |

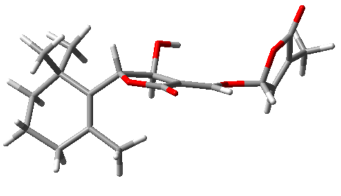

1ae (36.9%)

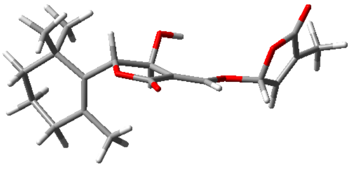

1be (21.1%)

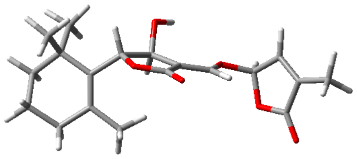

1ce (15.9%)

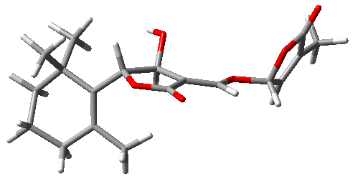

1de (7.8%)

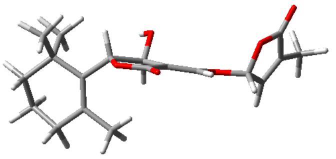

1ee (5.1%)

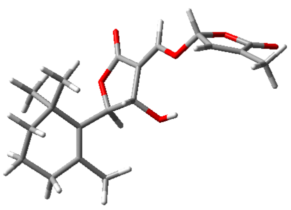

1fe (4.4%)

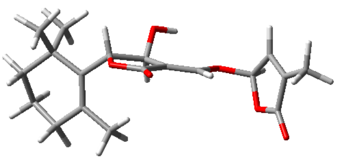

1ge (4.0%)

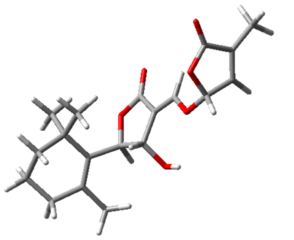

1he (1.3%)

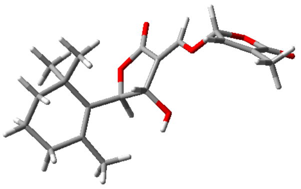

1ie (1.1%)

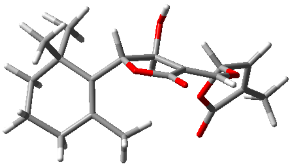

1je (0.8%)

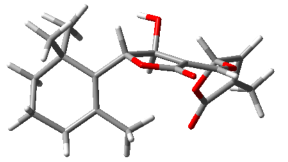

1ke (0.7%)

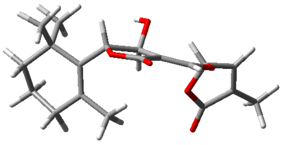

1le (0.4%)

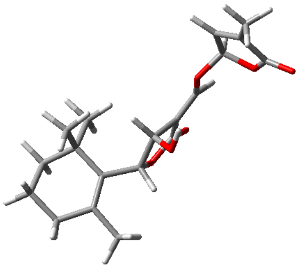

1me (0.3%)

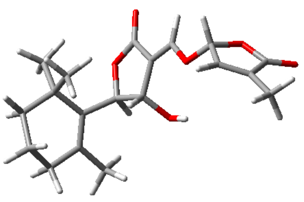

1ne (0.2%)

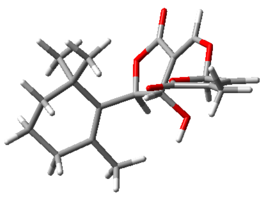

1oe (0.1%)

**Supplementary Data 2.26. Low-energy conformers of 4*R**,5*R**,2'*R** isomer optimized at the ωB97X-D/TZVP level in acetonitrile (PCM) for ECD calculation.**

**Supplementary Data 2.27. Cartesian coordinates of the conformers 1ae−1je of 4*S*,5*R*,2'*R* isomer for ECD calculation.**

Conformer 1ae

| Center Number | Atomic Number | Atomic Type | Coordinates (Angstroms) X | Y | Z |
|---|---|---|---|---|---|
| 1 | 6 | 0 | -4.87101 | -1.741298 | 0.342247 |
| 2 | 6 | 0 | -5.52984 | -0.620585 | 1.125775 |
| 3 | 6 | 0 | -4.47734 | 0.083792 | 1.963839 |
| 4 | 6 | 0 | -3.21 | 0.391401 | 1.205342 |
| 5 | 6 | 0 | -2.92277 | -0.168192 | 0.024412 |
| 6 | 6 | 0 | -3.80288 | -1.232077 | -0.63891 |
| 7 | 6 | 0 | -2.95762 | -2.446815 | -1.05842 |
| 8 | 6 | 0 | -4.48306 | -0.643394 | -1.88682 |
| 9 | 6 | 0 | -2.35133 | 1.407541 | 1.911079 |
| 10 | 6 | 0 | -1.70965 | 0.243518 | -0.78069 |
| 11 | 6 | 0 | -0.31404 | -0.135304 | -0.22729 |
| 12 | 6 | 0 | 0.410236 | 1.176105 | -0.26874 |
| 13 | 6 | 0 | -0.49055 | 2.224333 | -0.75864 |
| 14 | 8 | 0 | -1.69469 | 1.681454 | -0.99901 |
| 15 | 8 | 0 | 0.241552 | -1.124285 | -1.07756 |
| 16 | 8 | 0 | -0.27722 | 3.399273 | -0.92991 |
| 17 | 6 | 0 | 1.674584 | 1.412452 | 0.065344 |
| 18 | 8 | 0 | 2.442972 | 0.417406 | 0.542416 |
| 19 | 6 | 0 | 3.81465 | 0.690284 | 0.691711 |
| 20 | 6 | 0 | 4.457152 | -0.395542 | 1.495682 |
| 21 | 6 | 0 | 5.3761 | -1.006967 | 0.761952 |
| 22 | 6 | 0 | 5.377921 | -0.354721 | -0.5773 |
| 23 | 8 | 0 | 4.451081 | 0.644316 | -0.57616 |
| 24 | 8 | 0 | 6.049038 | -0.595519 | -1.53779 |
| 25 | 6 | 0 | 6.28312 | -2.139408 | 1.075299 |
| 26 | 1 | 0 | -4.39909 | -2.430888 | 1.050731 |
| 27 | 1 | 0 | -5.61066 | -2.320693 | -0.21665 |
| 28 | 1 | 0 | -6.32268 | -1.012898 | 1.76569 |
| 29 | 1 | 0 | -5.99867 | 0.093759 | 0.443416 |
| 30 | 1 | 0 | -4.21551 | -0.531142 | 2.833894 |
| 31 | 1 | 0 | -4.86926 | 1.020553 | 2.371842 |
| 32 | 1 | 0 | -2.24216 | -2.214686 | -1.84823 |
| 33 | 1 | 0 | -2.40086 | -2.844756 | -0.20698 |
| 34 | 1 | 0 | -3.6161 | -3.234681 | -1.43265 |
| 35 | 1 | 0 | -3.75473 | -0.256908 | -2.60297 |
| 36 | 1 | 0 | -5.06531 | -1.415935 | -2.39543 |
| 37 | 1 | 0 | -5.15427 | 0.175899 | -1.62443 |
| 38 | 1 | 0 | -2.41455 | 1.250404 | 2.990196 |
| 39 | 1 | 0 | -1.29849 | 1.377247 | 1.64164 |
| 40 | 1 | 0 | -2.71841 | 2.418325 | 1.712466 |
| 41 | 1 | 0 | -1.76304 | -0.192681 | -1.7758 |
| 42 | 1 | 0 | -0.38993 | -0.524475 | 0.79105 |
| 43 | 1 | 0 | 1.079135 | -1.403889 | -0.69867 |
| 44 | 1 | 0 | 2.12691 | 2.39577 | -0.02593 |
| 45 | 1 | 0 | 3.956295 | 1.691412 | 1.104229 |
| 46 | 1 | 0 | 4.167314 | -0.599395 | 2.515396 |
| 47 | 1 | 0 | 7.323336 | -1.843056 | 0.928816 |
| 48 | 1 | 0 | 6.150305 | -2.471725 | 2.102999 |
| 49 | 1 | 0 | 6.086499 | -2.97746 | 0.404111 |

Conformer 1be

| Center Number | Atomic Number | Atomic Type | Coordinates (Angstroms) X | Y | Z |
|---|---|---|---|---|---|
| 1 | 6 | 0 | -5.28431 | -1.042034 | -0.31794 |
| 2 | 6 | 0 | -5.05503 | -1.788858 | 0.983389 |
| 3 | 6 | 0 | -4.31734 | -0.879839 | 1.950187 |
| 4 | 6 | 0 | -3.13111 | -0.185072 | 1.328843 |
| 5 | 6 | 0 | -2.95796 | -0.102772 | 0.004195 |
| 6 | 6 | 0 | -3.97329 | -0.63809 | -1.01197 |
| 7 | 6 | 0 | -3.37892 | -1.851867 | -1.74635 |
| 8 | 6 | 0 | -4.32605 | 0.447314 | -2.04407 |
| 9 | 6 | 0 | -2.19631 | 0.381261 | 2.363349 |
| 10 | 6 | 0 | -1.72337 | 0.519856 | -0.60773 |
| 11 | 6 | 0 | -0.34417 | -0.026925 | -0.17482 |
| 12 | 6 | 0 | 0.422638 | 1.232648 | 0.093671 |
| 13 | 6 | 0 | -0.46138 | 2.392691 | -0.06124 |
| 14 | 8 | 0 | -1.69135 | 1.959071 | -0.3869 |
| 15 | 8 | 0 | 0.161104 | -0.803951 | -1.24759 |
| 16 | 8 | 0 | -0.21969 | 3.565536 | 0.084198 |
| 17 | 6 | 0 | 1.702175 | 1.348962 | 0.433964 |
| 18 | 8 | 0 | 2.458739 | 0.251439 | 0.608186 |
| 19 | 6 | 0 | 3.835827 | 0.456224 | 0.809304 |
| 20 | 6 | 0 | 4.469707 | -0.817688 | 1.271405 |
| 21 | 6 | 0 | 5.3742 | -1.210513 | 0.385762 |
| 22 | 6 | 0 | 5.373333 | -0.211455 | -0.71923 |
| 23 | 8 | 0 | 4.460357 | 0.757705 | -0.42911 |
| 24 | 8 | 0 | 6.032729 | -0.182814 | -1.7169 |
| 25 | 6 | 0 | 6.26867 | -2.39485 | 0.361634 |
| 26 | 1 | 0 | -5.88 | -1.63678 | -1.01547 |
| 27 | 1 | 0 | -5.86211 | -0.136933 | -0.10095 |
| 28 | 1 | 0 | -4.46674 | -2.69316 | 0.803587 |
| 29 | 1 | 0 | -6.00512 | -2.109751 | 1.415048 |
| 30 | 1 | 0 | -3.96942 | -1.440499 | 2.823006 |
| 31 | 1 | 0 | -4.99958 | -0.114973 | 2.341318 |
| 32 | 1 | 0 | -2.42529 | -1.61126 | -2.22126 |
| 33 | 1 | 0 | -3.20276 | -2.681434 | -1.05914 |
| 34 | 1 | 0 | -4.0644 | -2.191275 | -2.52712 |
| 35 | 1 | 0 | -3.49148 | 0.6992 | -2.70044 |
| 36 | 1 | 0 | -5.13951 | 0.090071 | -2.68023 |
| 37 | 1 | 0 | -4.65909 | 1.361829 | -1.54856 |
| 38 | 1 | 0 | -1.65961 | -0.425866 | 2.871064 |
| 39 | 1 | 0 | -1.46519 | 1.087205 | 1.980868 |
| 40 | 1 | 0 | -2.77878 | 0.899149 | 3.129627 |
| 41 | 1 | 0 | -1.74791 | 0.396087 | -1.6883 |
| 42 | 1 | 0 | -0.4211 | -0.646679 | 0.720433 |
| 43 | 1 | 0 | 0.973056 | -1.225178 | -0.95409 |
| 44 | 1 | 0 | 2.176983 | 2.313922 | 0.585617 |
| 45 | 1 | 0 | 3.9962 | 1.300964 | 1.482753 |
| 46 | 1 | 0 | 4.185792 | -1.294662 | 2.197285 |
| 47 | 1 | 0 | 7.311806 | -2.080268 | 0.295978 |
| 48 | 1 | 0 | 6.136422 | -3.000754 | 1.255903 |
| 49 | 1 | 0 | 6.058787 | -3.008806 | -0.51612 |

**Supplementary Data 2.27. Cartesian coordinates of the conformers 1ae−1je of 4*S*,5*R*,2'*R* isomer for ECD calculation (continued).**

Conformer 1ce

| Center Number | Atomic Number | Atomic Type | Coordinates (Angstroms) | | |
|---|---|---|---|---|---|
| | | | X | Y | Z |
| 1 | 6 | 0 | 5.236354 | 0.133386 | -0.98638 |
| 2 | 6 | 0 | 5.353954 | 1.550751 | -0.45586 |
| 3 | 6 | 0 | 4.021164 | 2.257324 | -0.63075 |
| 4 | 6 | 0 | 2.837984 | 1.427567 | -0.19776 |
| 5 | 6 | 0 | 2.924446 | 0.111308 | 0.025931 |
| 6 | 6 | 0 | 4.198612 | -0.702511 | -0.22011 |
| 7 | 6 | 0 | 3.887599 | -1.93925 | -1.08046 |
| 8 | 6 | 0 | 4.793583 | -1.164525 | 1.121042 |
| 9 | 6 | 0 | 1.590383 | 2.252663 | -0.0203 |
| 10 | 6 | 0 | 1.763658 | -0.684252 | 0.582176 |
| 11 | 6 | 0 | 0.534943 | -0.902875 | -0.33441 |
| 12 | 6 | 0 | -0.59385 | -0.425509 | 0.527311 |
| 13 | 6 | 0 | -0.0717 | 0.028916 | 1.820044 |
| 14 | 8 | 0 | 1.265445 | -0.088909 | 1.811815 |
| 15 | 8 | 0 | 0.469645 | -2.283098 | -0.65466 |
| 16 | 8 | 0 | -0.66546 | 0.466425 | 2.774935 |
| 17 | 6 | 0 | -1.8873 | -0.382287 | 0.223935 |
| 18 | 8 | 0 | -2.31188 | -0.812944 | -0.97612 |
| 19 | 6 | 0 | -3.67529 | -0.65241 | -1.29385 |
| 20 | 6 | 0 | -4.64328 | -1.191326 | -0.2822 |
| 21 | 6 | 0 | -5.47506 | -0.230479 | 0.097252 |
| 22 | 6 | 0 | -5.07181 | 1.010265 | -0.62244 |
| 23 | 8 | 0 | -3.99051 | 0.728628 | -1.40331 |
| 24 | 8 | 0 | -5.55677 | 2.102819 | -0.58185 |
| 25 | 6 | 0 | -6.61369 | -0.241742 | 1.048801 |
| 26 | 1 | 0 | 4.943274 | 0.179516 | -2.04097 |
| 27 | 1 | 0 | 6.198553 | -0.384239 | -0.94866 |
| 28 | 1 | 0 | 6.142125 | 2.094125 | -0.98077 |
| 29 | 1 | 0 | 5.629807 | 1.537299 | 0.602231 |
| 30 | 1 | 0 | 3.878162 | 2.536639 | -1.68206 |
| 31 | 1 | 0 | 4.003812 | 3.197424 | -0.07074 |
| 32 | 1 | 0 | 3.246229 | -2.660451 | -0.5724 |
| 33 | 1 | 0 | 3.39514 | -1.649814 | -2.01168 |
| 34 | 1 | 0 | 4.820558 | -2.449325 | -1.33308 |
| 35 | 1 | 0 | 4.079483 | -1.751308 | 1.702828 |
| 36 | 1 | 0 | 5.669278 | -1.794316 | 0.944805 |
| 37 | 1 | 0 | 5.099182 | -0.314831 | 1.733636 |
| 38 | 1 | 0 | 1.576944 | 3.055454 | -0.76067 |
| 39 | 1 | 0 | 0.662161 | 1.697059 | -0.13156 |
| 40 | 1 | 0 | 1.582125 | 2.725577 | 0.96566 |
| 41 | 1 | 0 | 2.10309 | -1.677455 | 0.867551 |
| 42 | 1 | 0 | 0.621081 | -0.31429 | -1.25105 |
| 43 | 1 | 0 | -0.23833 | -2.406633 | -1.29207 |
| 44 | 1 | 0 | -2.62841 | -0.017972 | 0.928058 |
| 45 | 1 | 0 | -3.78219 | -1.111681 | -2.27423 |
| 46 | 1 | 0 | -4.62756 | -2.224754 | 0.030979 |
| 47 | 1 | 0 | -7.52761 | 0.082888 | 0.548126 |
| 48 | 1 | 0 | -6.76782 | -1.237447 | 1.459671 |
| 49 | 1 | 0 | -6.42718 | 0.455089 | 1.868053 |

Conformer 1de

| Center Number | Atomic Number | Atomic Type | Coordinates (Angstroms) | | |
|---|---|---|---|---|---|
| | | | X | Y | Z |
| 1 | 6 | 0 | -4.89682 | -1.716579 | 0.314974 |
| 2 | 6 | 0 | -5.50447 | -0.660488 | 1.220652 |
| 3 | 6 | 0 | -4.4039 | -0.026061 | 2.053091 |
| 4 | 6 | 0 | -3.17348 | 0.330057 | 1.256572 |
| 5 | 6 | 0 | -2.95837 | -0.129076 | 0.018528 |
| 6 | 6 | 0 | -3.88407 | -1.129192 | -0.68067 |
| 7 | 6 | 0 | -3.07107 | -2.305761 | -1.24907 |
| 8 | 6 | 0 | -4.62961 | -0.438261 | -1.83481 |
| 9 | 6 | 0 | -2.25676 | 1.266571 | 1.998803 |
| 10 | 6 | 0 | -1.7761 | 0.318942 | -0.81006 |
| 11 | 6 | 0 | -0.36629 | -0.179769 | -0.37032 |
| 12 | 6 | 0 | 0.408916 | 1.095189 | -0.30847 |
| 13 | 6 | 0 | -0.45874 | 2.224079 | -0.66395 |
| 14 | 8 | 0 | -1.70034 | 1.767163 | -0.89153 |
| 15 | 8 | 0 | 0.204523 | -1.063413 | -1.31918 |
| 16 | 8 | 0 | -0.19231 | 3.398753 | -0.73746 |
| 17 | 6 | 0 | 1.671649 | 1.286949 | 0.066431 |
| 18 | 8 | 0 | 2.445644 | 0.274578 | 0.477857 |
| 19 | 6 | 0 | 3.792931 | 0.581971 | 0.741739 |
| 20 | 6 | 0 | 4.43604 | -0.561743 | 1.460519 |
| 21 | 6 | 0 | 5.42473 | -1.049316 | 0.724624 |
| 22 | 6 | 0 | 5.475669 | -0.250598 | -0.53161 |
| 23 | 8 | 0 | 4.504617 | 0.703523 | -0.48052 |
| 24 | 8 | 0 | 6.213923 | -0.354493 | -1.46731 |
| 25 | 6 | 0 | 6.362616 | -2.173526 | 0.968963 |
| 26 | 1 | 0 | -4.38884 | -2.460625 | 0.938361 |
| 27 | 1 | 0 | -5.67 | -2.248747 | -0.24543 |
| 28 | 1 | 0 | -6.26324 | -1.101426 | 1.870065 |
| 29 | 1 | 0 | -6.00624 | 0.106131 | 0.623886 |
| 30 | 1 | 0 | -4.10348 | -0.705642 | 2.860306 |
| 31 | 1 | 0 | -4.76578 | 0.879616 | 2.549566 |
| 32 | 1 | 0 | -2.42434 | -2.01185 | -2.07812 |
| 33 | 1 | 0 | -2.45654 | -2.766569 | -0.47077 |
| 34 | 1 | 0 | -3.75178 | -3.069732 | -1.63174 |
| 35 | 1 | 0 | -3.94009 | 0.00924 | -2.55378 |
| 36 | 1 | 0 | -5.2415 | -1.165473 | -2.3745 |
| 37 | 1 | 0 | -5.28264 | 0.354294 | -1.46651 |
| 38 | 1 | 0 | -2.28061 | 1.029726 | 3.064757 |
| 39 | 1 | 0 | -1.21746 | 1.224382 | 1.681632 |
| 40 | 1 | 0 | -2.59807 | 2.29992 | 1.891903 |
| 41 | 1 | 0 | -1.90195 | -0.011446 | -1.83901 |
| 42 | 1 | 0 | -0.41833 | -0.668665 | 0.60575 |
| 43 | 1 | 0 | -0.15218 | -1.942004 | -1.16706 |
| 44 | 1 | 0 | 2.111302 | 2.280786 | 0.0653 |
| 45 | 1 | 0 | 3.866364 | 1.535168 | 1.26982 |
| 46 | 1 | 0 | 4.095593 | -0.891768 | 2.430371 |
| 47 | 1 | 0 | 7.395111 | -1.822464 | 0.92338 |
| 48 | 1 | 0 | 6.183902 | -2.624499 | 1.943061 |
| 49 | 1 | 0 | 6.244957 | -2.937572 | 0.198307 |

**Supplementary Data 2.27. Cartesian coordinates of the conformers 1ae−1je of 4*S*,5*R*,2'*R* isomer for ECD calculation (continued).**

Conformer 1ee

| Center Number | Atomic Number | Atomic Type | Coordinates (Angstroms) X | Y | Z |
|---|---|---|---|---|---|
| 1 | 6 | 0 | -5.33301 | -0.964101 | -0.33238 |
| 2 | 6 | 0 | -5.04348 | -1.904562 | 0.823208 |
| 3 | 6 | 0 | -4.25048 | -1.156253 | 1.879883 |
| 4 | 6 | 0 | -3.08912 | -0.378142 | 1.312753 |
| 5 | 6 | 0 | -2.98316 | -0.090448 | 0.009701 |
| 6 | 6 | 0 | -4.05616 | -0.456534 | -1.02222 |
| 7 | 6 | 0 | -3.51338 | -1.539506 | -1.97023 |
| 8 | 6 | 0 | -4.45123 | 0.7791 | -1.84938 |
| 9 | 6 | 0 | -2.09535 | 0.016352 | 2.371624 |
| 10 | 6 | 0 | -1.77063 | 0.605635 | -0.5644 |
| 11 | 6 | 0 | -0.37514 | -0.024212 | -0.29219 |
| 12 | 6 | 0 | 0.42159 | 1.164197 | 0.137326 |
| 13 | 6 | 0 | -0.44111 | 2.350608 | 0.189295 |
| 14 | 8 | 0 | -1.69582 | 1.994513 | -0.13429 |
| 15 | 8 | 0 | 0.167018 | -0.60504 | -1.46571 |
| 16 | 8 | 0 | -0.16297 | 3.485115 | 0.49232 |
| 17 | 6 | 0 | 1.698731 | 1.225258 | 0.507743 |
| 18 | 8 | 0 | 2.470704 | 0.131846 | 0.556333 |
| 19 | 6 | 0 | 3.833329 | 0.335219 | 0.840258 |
| 20 | 6 | 0 | 4.479053 | -0.976883 | 1.156332 |
| 21 | 6 | 0 | 5.425579 | -1.23258 | 0.264548 |
| 22 | 6 | 0 | 5.445118 | -0.095344 | -0.69705 |
| 23 | 8 | 0 | 4.498872 | 0.809531 | -0.32079 |
| 24 | 8 | 0 | 6.143596 | 0.077947 | -1.65287 |
| 25 | 6 | 0 | 6.346249 | -2.387927 | 0.119699 |
| 26 | 1 | 0 | -5.96932 | -1.442074 | -1.08183 |
| 27 | 1 | 0 | -5.8908 | -0.103689 | 0.05303 |
| 28 | 1 | 0 | -4.47202 | -2.769737 | 0.475028 |
| 29 | 1 | 0 | -5.97284 | -2.288816 | 1.248134 |
| 30 | 1 | 0 | -3.86581 | -1.844836 | 2.638138 |
| 31 | 1 | 0 | -4.90518 | -0.458774 | 2.416792 |
| 32 | 1 | 0 | -2.60131 | -1.214507 | -2.47695 |
| 33 | 1 | 0 | -3.28373 | -2.460053 | -1.43045 |
| 34 | 1 | 0 | -4.25143 | -1.768524 | -2.74297 |
| 35 | 1 | 0 | -3.64667 | 1.135139 | -2.49482 |
| 36 | 1 | 0 | -5.29571 | 0.527035 | -2.49544 |
| 37 | 1 | 0 | -4.75543 | 1.601174 | -1.19803 |
| 38 | 1 | 0 | -1.55582 | -0.864922 | 2.731396 |
| 39 | 1 | 0 | -1.36537 | 0.757326 | 2.059849 |
| 40 | 1 | 0 | -2.63022 | 0.427435 | 3.231724 |
| 41 | 1 | 0 | -1.85171 | 0.648762 | -1.64844 |
| 42 | 1 | 0 | -0.42669 | -0.776386 | 0.496907 |
| 43 | 1 | 0 | -0.1487 | -1.509264 | -1.52962 |
| 44 | 1 | 0 | 2.153035 | 2.168452 | 0.799186 |
| 45 | 1 | 0 | 3.949043 | 1.086538 | 1.624656 |
| 46 | 1 | 0 | 4.170448 | -1.577857 | 1.998351 |
| 47 | 1 | 0 | 7.384049 | -2.050225 | 0.13805 |
| 48 | 1 | 0 | 6.193931 | -3.109843 | 0.919641 |
| 49 | 1 | 0 | 6.182633 | -2.882918 | -0.83937 |

Conformer 1fe

| Center Number | Atomic Number | Atomic Type | Coordinates (Angstroms) X | Y | Z |
|---|---|---|---|---|---|
| 1 | 6 | 0 | -4.38091 | -0.560347 | 1.759226 |
| 2 | 6 | 0 | -5.42763 | -1.236686 | 0.893229 |
| 3 | 6 | 0 | -4.7348 | -2.101859 | -0.14469 |
| 4 | 6 | 0 | -3.51811 | -1.466585 | -0.76832 |
| 5 | 6 | 0 | -2.94326 | -0.352447 | -0.2931 |
| 6 | 6 | 0 | -3.45939 | 0.376212 | 0.957338 |
| 7 | 6 | 0 | -4.24599 | 1.6412 | 0.562664 |
| 8 | 6 | 0 | -2.32341 | 0.787658 | 1.906622 |
| 9 | 6 | 0 | -3.02069 | -2.228923 | -1.977 |
| 10 | 6 | 0 | -1.72588 | 0.173747 | -1.02519 |
| 11 | 6 | 0 | -0.34434 | -0.223665 | -0.4467 |
| 12 | 6 | 0 | 0.348991 | 1.098184 | -0.3312 |
| 13 | 6 | 0 | -0.55162 | 2.165578 | -0.77493 |
| 14 | 8 | 0 | -1.72893 | 1.620618 | -1.12355 |
| 15 | 8 | 0 | 0.265715 | -1.120446 | -1.36191 |
| 16 | 8 | 0 | -0.36222 | 3.355319 | -0.83514 |
| 17 | 6 | 0 | 1.584032 | 1.334709 | 0.098436 |
| 18 | 8 | 0 | 2.357496 | 0.321597 | 0.526216 |
| 19 | 6 | 0 | 3.701026 | 0.628026 | 0.808203 |
| 20 | 6 | 0 | 4.337068 | -0.520659 | 1.525277 |
| 21 | 6 | 0 | 5.334029 | -1.001923 | 0.796344 |
| 22 | 6 | 0 | 5.398515 | -0.193168 | -0.45293 |
| 23 | 8 | 0 | 4.426219 | 0.76021 | -0.40486 |
| 24 | 8 | 0 | 6.146674 | -0.289416 | -1.38138 |
| 25 | 6 | 0 | 6.270162 | -2.127242 | 1.042184 |
| 26 | 1 | 0 | -4.85081 | 0.017744 | 2.559499 |
| 27 | 1 | 0 | -3.76886 | -1.331415 | 2.240441 |
| 28 | 1 | 0 | -6.04953 | -0.485723 | 0.399711 |
| 29 | 1 | 0 | -6.09706 | -1.84514 | 1.504619 |
| 30 | 1 | 0 | -5.42666 | -2.375507 | -0.94755 |
| 31 | 1 | 0 | -4.42089 | -3.051465 | 0.306582 |
| 32 | 1 | 0 | -3.60263 | 2.371116 | 0.075394 |
| 33 | 1 | 0 | -5.0611 | 1.40493 | -0.12278 |
| 34 | 1 | 0 | -4.67433 | 2.102481 | 1.456901 |
| 35 | 1 | 0 | -1.65286 | 1.530325 | 1.475175 |
| 36 | 1 | 0 | -2.75516 | 1.235035 | 2.805061 |
| 37 | 1 | 0 | -1.73207 | -0.074787 | 2.22095 |
| 38 | 1 | 0 | -3.41858 | -1.792478 | -2.89863 |
| 39 | 1 | 0 | -1.93582 | -2.262827 | -2.05636 |
| 40 | 1 | 0 | -3.3788 | -3.258695 | -1.9341 |
| 41 | 1 | 0 | -1.73914 | -0.177489 | -2.05381 |
| 42 | 1 | 0 | -0.45064 | -0.708543 | 0.525372 |
| 43 | 1 | 0 | 1.082149 | -1.438509 | -0.96773 |
| 44 | 1 | 0 | 2.00851 | 2.334069 | 0.124615 |
| 45 | 1 | 0 | 3.765491 | 1.577283 | 1.344291 |
| 46 | 1 | 0 | 3.986323 | -0.858279 | 2.488818 |
| 47 | 1 | 0 | 7.302672 | -1.774571 | 1.011967 |
| 48 | 1 | 0 | 6.080408 | -2.586776 | 2.010159 |
| 49 | 1 | 0 | 6.162723 | -2.884691 | 0.263572 |

**Supplementary Data 2.27. Cartesian coordinates of the conformers 1ae−1je of 4*S*,5*R*,2'*R* isomer for ECD calculation (continued).**

Conformer 1ge

| Center Number | Atomic Number | Atomic Type | Coordinates (Angstroms) X | Y | Z |
|---|---|---|---|---|---|
| 1 | 6 | 0 | 5.482494 | 0.265589 | -0.02539 |
| 2 | 6 | 0 | 5.245926 | 1.644012 | -0.61512 |
| 3 | 6 | 0 | 4.098548 | 2.307453 | 0.125555 |
| 4 | 6 | 0 | 2.899018 | 1.408688 | 0.296561 |
| 5 | 6 | 0 | 2.964502 | 0.081867 | 0.133092 |
| 6 | 6 | 0 | 4.26745 | -0.664211 | -0.17679 |
| 7 | 6 | 0 | 4.215044 | -1.221213 | -1.60959 |
| 8 | 6 | 0 | 4.468601 | -1.83089 | 0.806114 |
| 9 | 6 | 0 | 1.658103 | 2.178335 | 0.659954 |
| 10 | 6 | 0 | 1.739952 | -0.800588 | 0.224748 |
| 11 | 6 | 0 | 0.532733 | -0.463072 | -0.67935 |
| 12 | 6 | 0 | -0.61203 | -0.530886 | 0.285168 |
| 13 | 6 | 0 | -0.10578 | -0.791795 | 1.636601 |
| 14 | 8 | 0 | 1.235528 | -0.867212 | 1.589626 |
| 15 | 8 | 0 | 0.489735 | -1.435329 | -1.71104 |
| 16 | 8 | 0 | -0.7121 | -0.906808 | 2.67322 |
| 17 | 6 | 0 | -1.90591 | -0.36806 | 0.027206 |
| 18 | 8 | 0 | -2.31569 | -0.121231 | -1.22855 |
| 19 | 6 | 0 | -3.6874 | 0.121209 | -1.4418 |
| 20 | 6 | 0 | -4.61871 | -0.920345 | -0.89507 |
| 21 | 6 | 0 | -5.49397 | -0.352411 | -0.07623 |
| 22 | 6 | 0 | -5.16088 | 1.098421 | -0.0139 |
| 23 | 8 | 0 | -4.07401 | 1.330035 | -0.80354 |
| 24 | 8 | 0 | -5.70007 | 1.975468 | 0.594821 |
| 25 | 6 | 0 | -6.6203 | -0.925836 | 0.701353 |
| 26 | 1 | 0 | 6.350335 | -0.216521 | -0.48317 |
| 27 | 1 | 0 | 5.709415 | 0.378752 | 1.040376 |
| 28 | 1 | 0 | 5.003529 | 1.565481 | -1.6787 |
| 29 | 1 | 0 | 6.14826 | 2.254087 | -0.5403 |
| 30 | 1 | 0 | 3.776547 | 3.216863 | -0.39056 |
| 31 | 1 | 0 | 4.430876 | 2.631993 | 1.119373 |
| 32 | 1 | 0 | 3.341151 | -1.857593 | -1.76507 |
| 33 | 1 | 0 | 4.168802 | -0.415603 | -2.34464 |
| 34 | 1 | 0 | 5.10565 | -1.821556 | -1.81239 |
| 35 | 1 | 0 | 3.739303 | -2.631646 | 0.673112 |
| 36 | 1 | 0 | 5.457981 | -2.267824 | 0.650308 |
| 37 | 1 | 0 | 4.409971 | -1.483373 | 1.839835 |
| 38 | 1 | 0 | 1.310214 | 2.761466 | -0.19802 |
| 39 | 1 | 0 | 0.830655 | 1.571217 | 1.014458 |
| 40 | 1 | 0 | 1.897415 | 2.895934 | 1.448957 |
| 41 | 1 | 0 | 2.006235 | -1.823617 | -0.03196 |
| 42 | 1 | 0 | 0.625501 | 0.531974 | -1.11852 |
| 43 | 1 | 0 | -0.18521 | -1.171066 | -2.34129 |
| 44 | 1 | 0 | -2.65879 | -0.440569 | 0.805506 |
| 45 | 1 | 0 | -3.77674 | 0.252365 | -2.51803 |
| 46 | 1 | 0 | -4.54792 | -1.958942 | -1.18246 |
| 47 | 1 | 0 | -6.71381 | -1.994647 | 0.519911 |
| 48 | 1 | 0 | -6.46457 | -0.759558 | 1.768825 |
| 49 | 1 | 0 | -7.55628 | -0.434616 | 0.429357 |

Conformer 1he

| Center Number | Atomic Number | Atomic Type | Coordinates (Angstroms) X | Y | Z |
|---|---|---|---|---|---|
| 1 | 6 | 0 | -4.1305 | -1.616338 | -1.23872 |
| 2 | 6 | 0 | -5.45725 | -0.943519 | -0.93873 |
| 3 | 6 | 0 | -5.26279 | 0.562414 | -0.92528 |
| 4 | 6 | 0 | -3.99975 | 1.017561 | -0.23954 |
| 5 | 6 | 0 | -3.01114 | 0.183525 | 0.113514 |
| 6 | 6 | 0 | -3.0598 | -1.325334 | -0.17175 |
| 7 | 6 | 0 | -3.39895 | -2.109464 | 1.110659 |
| 8 | 6 | 0 | -1.73178 | -1.859342 | -0.73052 |
| 9 | 6 | 0 | -3.97097 | 2.512369 | -0.00505 |
| 10 | 6 | 0 | -1.80134 | 0.785212 | 0.798725 |
| 11 | 6 | 0 | -0.58208 | 1.105484 | -0.10258 |
| 12 | 6 | 0 | 0.53868 | 0.421183 | 0.6155 |
| 13 | 6 | 0 | 0.021639 | -0.236734 | 1.818342 |
| 14 | 8 | 0 | -1.30759 | -0.051341 | 1.8754 |
| 15 | 8 | 0 | -0.45362 | 2.518007 | -0.16669 |
| 16 | 8 | 0 | 0.608495 | -0.873177 | 2.658543 |
| 17 | 6 | 0 | 1.820122 | 0.364819 | 0.267076 |
| 18 | 8 | 0 | 2.242849 | 0.979129 | -0.85074 |
| 19 | 6 | 0 | 3.595909 | 0.830111 | -1.21559 |
| 20 | 6 | 0 | 4.592172 | 1.183923 | -0.15101 |
| 21 | 6 | 0 | 5.398301 | 0.153672 | 0.067684 |
| 22 | 6 | 0 | 4.949452 | -0.95306 | -0.82328 |
| 23 | 8 | 0 | 3.86888 | -0.526515 | -1.53684 |
| 24 | 8 | 0 | 5.400798 | -2.05288 | -0.95398 |
| 25 | 6 | 0 | 6.546121 | -0.011031 | 0.993764 |
| 26 | 1 | 0 | -4.25231 | -2.699793 | -1.32067 |
| 27 | 1 | 0 | -3.76669 | -1.262441 | -2.20984 |
| 28 | 1 | 0 | -5.83934 | -1.280211 | 0.02817 |
| 29 | 1 | 0 | -6.20525 | -1.220019 | -1.68452 |
| 30 | 1 | 0 | -6.11091 | 1.059736 | -0.44416 |
| 31 | 1 | 0 | -5.24236 | 0.948137 | -1.95229 |
| 32 | 1 | 0 | -2.61658 | -2.000975 | 1.859258 |
| 33 | 1 | 0 | -4.33408 | -1.76292 | 1.552907 |
| 34 | 1 | 0 | -3.50538 | -3.172112 | 0.875306 |
| 35 | 1 | 0 | -0.91063 | -1.7957 | -0.01728 |
| 36 | 1 | 0 | -1.85109 | -2.915807 | -0.98233 |
| 37 | 1 | 0 | -1.44137 | -1.33514 | -1.64329 |
| 38 | 1 | 0 | -4.29227 | 2.750168 | 1.013979 |
| 39 | 1 | 0 | -2.99189 | 2.9598 | -0.16635 |
| 40 | 1 | 0 | -4.67216 | 3.003908 | -0.6811 |
| 41 | 1 | 0 | -2.08174 | 1.720425 | 1.276669 |
| 42 | 1 | 0 | -0.71959 | 0.70471 | -1.10835 |
| 43 | 1 | 0 | 0.22563 | 2.726266 | -0.81313 |
| 44 | 1 | 0 | 2.553406 | -0.161057 | 0.870028 |
| 45 | 1 | 0 | 3.702583 | 1.429266 | -2.11733 |
| 46 | 1 | 0 | 4.61097 | 2.158581 | 0.313723 |
| 47 | 1 | 0 | 7.447608 | -0.270851 | 0.435899 |
| 48 | 1 | 0 | 6.727109 | 0.902974 | 1.555836 |
| 49 | 1 | 0 | 6.351528 | -0.825425 | 1.693946 |

**Supplementary Data 2.27. Cartesian coordinates of the conformers 1ae−1je of 4*S*,5*R*,2'*R* isomer for ECD calculation (continued).**

Conformer 1ie

| Center Number | Atomic Number | Atomic Type | Coordinates (Angstroms) | | |
|---|---|---|---|---|---|
| | | | X | Y | Z |
| 1 | 6 | 0 | -4.3555 | -0.564366 | 1.797548 |
| 2 | 6 | 0 | -5.439 | -1.200518 | 0.94756 |
| 3 | 6 | 0 | -4.788 | -2.050544 | -0.12814 |
| 4 | 6 | 0 | -3.59125 | -1.4088 | -0.78782 |
| 5 | 6 | 0 | -2.97825 | -0.319612 | -0.29932 |
| 6 | 6 | 0 | -3.44224 | 0.37637 | 0.991695 |
| 7 | 6 | 0 | -4.21825 | 1.665235 | 0.65709 |
| 8 | 6 | 0 | -2.27048 | 0.740857 | 1.916588 |
| 9 | 6 | 0 | -3.18514 | -2.164252 | -2.02974 |
| 10 | 6 | 0 | -1.77022 | 0.215497 | -1.03844 |
| 11 | 6 | 0 | -0.37784 | -0.24909 | -0.51397 |
| 12 | 6 | 0 | 0.359993 | 1.042958 | -0.39273 |
| 13 | 6 | 0 | -0.52128 | 2.153032 | -0.76819 |
| 14 | 8 | 0 | -1.73521 | 1.664488 | -1.07156 |
| 15 | 8 | 0 | 0.258995 | -1.118602 | -1.43511 |
| 16 | 8 | 0 | -0.29061 | 3.336965 | -0.80325 |
| 17 | 6 | 0 | 1.59878 | 1.26136 | 0.041895 |
| 18 | 8 | 0 | 2.380219 | 0.261094 | 0.470378 |
| 19 | 6 | 0 | 3.709561 | 0.595359 | 0.785598 |
| 20 | 6 | 0 | 4.353479 | -0.542064 | 1.513554 |
| 21 | 6 | 0 | 5.378944 | -0.99749 | 0.808038 |
| 22 | 6 | 0 | 5.455425 | -0.182654 | -0.43649 |
| 23 | 8 | 0 | 4.461214 | 0.748162 | -0.4092 |
| 24 | 8 | 0 | 6.228619 | -0.258191 | -1.3464 |
| 25 | 6 | 0 | 6.334139 | -2.102287 | 1.073199 |
| 26 | 1 | 0 | -4.78979 | 0.001458 | 2.626146 |
| 27 | 1 | 0 | -3.74356 | -1.358213 | 2.240367 |
| 28 | 1 | 0 | -6.06239 | -0.428344 | 0.490084 |
| 29 | 1 | 0 | -6.10019 | -1.813786 | 1.56308 |
| 30 | 1 | 0 | -5.51011 | -2.313429 | -0.90728 |
| 31 | 1 | 0 | -4.45911 | -3.005371 | 0.300581 |
| 32 | 1 | 0 | -3.57829 | 2.396815 | 0.168141 |
| 33 | 1 | 0 | -5.06017 | 1.461545 | -0.00603 |
| 34 | 1 | 0 | -4.60798 | 2.109013 | 1.57741 |
| 35 | 1 | 0 | -1.59879 | 1.482232 | 1.485323 |
| 36 | 1 | 0 | -2.66743 | 1.171104 | 2.839086 |
| 37 | 1 | 0 | -1.6877 | -0.140795 | 2.191255 |
| 38 | 1 | 0 | -3.9199 | -1.998847 | -2.82332 |
| 39 | 1 | 0 | -2.20071 | -1.924176 | -2.42186 |
| 40 | 1 | 0 | -3.19403 | -3.235944 | -1.8153 |
| 41 | 1 | 0 | -1.81443 | -0.081692 | -2.0818 |
| 42 | 1 | 0 | -0.47239 | -0.74667 | 0.4523 |
| 43 | 1 | 0 | 0.028527 | -2.022322 | -1.20965 |
| 44 | 1 | 0 | 2.012723 | 2.265541 | 0.077175 |
| 45 | 1 | 0 | 3.742711 | 1.543397 | 1.326912 |
| 46 | 1 | 0 | 3.986779 | -0.891755 | 2.466794 |
| 47 | 1 | 0 | 7.358772 | -1.726106 | 1.070774 |
| 48 | 1 | 0 | 6.130452 | -2.570404 | 2.034242 |
| 49 | 1 | 0 | 6.263869 | -2.858523 | 0.289168 |

Conformer 1je

| Center Number | Atomic Number | Atomic Type | Coordinates (Angstroms) | | |
|---|---|---|---|---|---|
| | | | X | Y | Z |
| 1 | 6 | 0 | 3.945904 | -2.100344 | 0.479413 |
| 2 | 6 | 0 | 4.345288 | -2.016829 | -0.98264 |
| 3 | 6 | 0 | 3.1088 | -1.736233 | -1.81825 |
| 4 | 6 | 0 | 2.232421 | -0.646979 | -1.25195 |
| 5 | 6 | 0 | 2.371083 | -0.188159 | -0.00286 |
| 6 | 6 | 0 | 3.355642 | -0.783396 | 1.008659 |
| 7 | 6 | 0 | 2.634933 | -1.108435 | 2.328682 |
| 8 | 6 | 0 | 4.490207 | 0.216012 | 1.291298 |
| 9 | 6 | 0 | 1.230484 | -0.133369 | -2.25281 |
| 10 | 6 | 0 | 1.571904 | 0.988749 | 0.513699 |
| 11 | 6 | 0 | 0.047342 | 0.773023 | 0.735564 |
| 12 | 6 | 0 | -0.56071 | 1.870738 | -0.08872 |
| 13 | 6 | 0 | 0.522005 | 2.687685 | -0.66764 |
| 14 | 8 | 0 | 1.706413 | 2.143004 | -0.35284 |
| 15 | 8 | 0 | -0.30499 | 0.813408 | 2.109864 |
| 16 | 8 | 0 | 0.445959 | 3.680619 | -1.34843 |
| 17 | 6 | 0 | -1.82067 | 2.192209 | -0.4052 |
| 18 | 8 | 0 | -2.98178 | 1.6055 | -0.10396 |
| 19 | 6 | 0 | -3.00354 | 0.375592 | 0.58598 |
| 20 | 6 | 0 | -4.42125 | 0.013528 | 0.895931 |
| 21 | 6 | 0 | -4.73371 | -1.125189 | 0.293389 |
| 22 | 6 | 0 | -3.5333 | -1.565231 | -0.4684 |
| 23 | 8 | 0 | -2.53559 | -0.658419 | -0.26935 |
| 24 | 8 | 0 | -3.37893 | -2.535245 | -1.15168 |
| 25 | 6 | 0 | -5.994 | -1.909751 | 0.278744 |
| 26 | 1 | 0 | 3.197682 | -2.892648 | 0.591161 |
| 27 | 1 | 0 | 4.796744 | -2.378808 | 1.106796 |
| 28 | 1 | 0 | 4.817692 | -2.946365 | -1.3063 |
| 29 | 1 | 0 | 5.080319 | -1.220636 | -1.12967 |
| 30 | 1 | 0 | 2.504392 | -2.646807 | -1.91434 |
| 31 | 1 | 0 | 3.385349 | -1.456591 | -2.83955 |
| 32 | 1 | 0 | 2.282092 | -0.218847 | 2.852321 |
| 33 | 1 | 0 | 1.774425 | -1.757598 | 2.151187 |
| 34 | 1 | 0 | 3.322561 | -1.630654 | 2.998537 |
| 35 | 1 | 0 | 4.110103 | 1.172824 | 1.655762 |
| 36 | 1 | 0 | 5.159121 | -0.184162 | 2.057397 |
| 37 | 1 | 0 | 5.0768 | 0.414969 | 0.393026 |
| 38 | 1 | 0 | 0.928208 | -0.947178 | -2.91508 |
| 39 | 1 | 0 | 0.323581 | 0.273712 | -1.81172 |
| 40 | 1 | 0 | 1.677517 | 0.642579 | -2.8804 |
| 41 | 1 | 0 | 1.982625 | 1.307958 | 1.469453 |
| 42 | 1 | 0 | -0.24776 | -0.218165 | 0.397354 |
| 43 | 1 | 0 | -0.18208 | 1.70946 | 2.438033 |
| 44 | 1 | 0 | -1.99253 | 3.072527 | -1.01371 |
| 45 | 1 | 0 | -2.35358 | 0.418771 | 1.461414 |
| 46 | 1 | 0 | -5.04313 | 0.628457 | 1.528828 |
| 47 | 1 | 0 | -5.81516 | -2.921746 | 0.646562 |
| 48 | 1 | 0 | -6.75373 | -1.436756 | 0.897961 |
| 49 | 1 | 0 | -6.37293 | -1.99696 | -0.7412 |

**Supplementary Data 2.27. Cartesian coordinates of the conformers 1ae−1je of 4*S*,5*R*,2'*R* isomer for ECD calculation (continued).**

Conformer 1ke

| Center Number | Atomic Number | Atomic Type | Coordinates (Angstroms) | | |
|---|---|---|---|---|---|
| | | | X | Y | Z |
| 1 | 6 | 0 | -3.94988 | -2.104953 | -0.45191 |
| 2 | 6 | 0 | -4.3791 | -1.987056 | 0.999396 |
| 3 | 6 | 0 | -3.16293 | -1.675877 | 1.854005 |
| 4 | 6 | 0 | -2.27664 | -0.601132 | 1.275884 |
| 5 | 6 | 0 | -2.38894 | -0.17787 | 0.011747 |
| 6 | 6 | 0 | -3.35723 | -0.797456 | -1.0006 |
| 7 | 6 | 0 | -2.61913 | -1.147547 | -2.30508 |
| 8 | 6 | 0 | -4.48858 | 0.193413 | -1.32176 |
| 9 | 6 | 0 | -1.29594 | -0.058612 | 2.282177 |
| 10 | 6 | 0 | -1.56987 | 0.973789 | -0.52662 |
| 11 | 6 | 0 | -0.04428 | 0.730447 | -0.71241 |
| 12 | 6 | 0 | 0.561757 | 1.866137 | 0.052256 |
| 13 | 6 | 0 | -0.52125 | 2.713135 | 0.587286 |
| 14 | 8 | 0 | -1.70661 | 2.158753 | 0.294793 |
| 15 | 8 | 0 | 0.333802 | 0.80088 | -2.08243 |
| 16 | 8 | 0 | -0.44379 | 3.735673 | 1.222603 |
| 17 | 6 | 0 | 1.817205 | 2.197883 | 0.374329 |
| 18 | 8 | 0 | 2.986111 | 1.607257 | 0.112778 |
| 19 | 6 | 0 | 3.027491 | 0.378213 | -0.57514 |
| 20 | 6 | 0 | 4.45144 | 0.026709 | -0.86789 |
| 21 | 6 | 0 | 4.764295 | -1.111671 | -0.26487 |
| 22 | 6 | 0 | 3.557584 | -1.562184 | 0.480563 |
| 23 | 8 | 0 | 2.556578 | -0.661183 | 0.272898 |
| 24 | 8 | 0 | 3.401348 | -2.535938 | 1.158289 |
| 25 | 6 | 0 | 6.029717 | -1.887608 | -0.23726 |
| 26 | 1 | 0 | -3.19504 | -2.895141 | -0.52973 |
| 27 | 1 | 0 | -4.7869 | -2.403546 | -1.08852 |
| 28 | 1 | 0 | -4.85159 | -2.911634 | 1.336647 |
| 29 | 1 | 0 | -5.12296 | -1.193354 | 1.110389 |
| 30 | 1 | 0 | -2.55709 | -2.580337 | 1.990278 |
| 31 | 1 | 0 | -3.46375 | -1.36811 | 2.860186 |
| 32 | 1 | 0 | -2.3069 | -0.263729 | -2.86537 |
| 33 | 1 | 0 | -1.74084 | -1.766582 | -2.10222 |
| 34 | 1 | 0 | -3.28291 | -1.716716 | -2.95987 |
| 35 | 1 | 0 | -4.10199 | 1.144464 | -1.69451 |
| 36 | 1 | 0 | -5.14255 | -0.220926 | -2.09324 |
| 37 | 1 | 0 | -5.09159 | 0.406299 | -0.43787 |
| 38 | 1 | 0 | -0.99406 | -0.857449 | 2.962757 |
| 39 | 1 | 0 | -0.38789 | 0.353942 | 1.848673 |
| 40 | 1 | 0 | -1.7631 | 0.721456 | 2.889635 |
| 41 | 1 | 0 | -1.9502 | 1.267743 | -1.50268 |
| 42 | 1 | 0 | 0.24084 | -0.240007 | -0.30253 |
| 43 | 1 | 0 | 0.050921 | -0.009692 | -2.5155 |
| 44 | 1 | 0 | 1.976658 | 3.098924 | 0.955119 |
| 45 | 1 | 0 | 2.387155 | 0.419058 | -1.45731 |
| 46 | 1 | 0 | 5.076923 | 0.647804 | -1.49116 |
| 47 | 1 | 0 | 5.862446 | -2.899662 | -0.61033 |
| 48 | 1 | 0 | 6.79359 | -1.40744 | -0.84577 |
| 49 | 1 | 0 | 6.396945 | -1.97547 | 0.786891 |

Conformer 1le

| Center Number | Atomic Number | Atomic Type | Coordinates (Angstroms) | | |
|---|---|---|---|---|---|
| | | | X | Y | Z |
| 1 | 6 | 0 | -4.53373 | -1.553314 | -0.17087 |
| 2 | 6 | 0 | -3.73457 | -2.713891 | 0.393155 |
| 3 | 6 | 0 | -2.8308 | -2.197915 | 1.498663 |
| 4 | 6 | 0 | -2.07186 | -0.951591 | 1.116367 |
| 5 | 6 | 0 | -2.41333 | -0.194721 | 0.06623 |
| 6 | 6 | 0 | -3.64487 | -0.470757 | -0.80442 |
| 7 | 6 | 0 | -3.19897 | -0.921946 | -2.20595 |
| 8 | 6 | 0 | -4.50401 | 0.798387 | -0.93805 |
| 9 | 6 | 0 | -0.91283 | -0.684398 | 2.038166 |
| 10 | 6 | 0 | -1.58958 | 0.994984 | -0.37198 |
| 11 | 6 | 0 | -0.07244 | 0.768524 | -0.61223 |
| 12 | 6 | 0 | 0.551095 | 1.876611 | 0.177903 |
| 13 | 6 | 0 | -0.51794 | 2.671259 | 0.813299 |
| 14 | 8 | 0 | -1.70744 | 2.10543 | 0.555775 |
| 15 | 8 | 0 | 0.249543 | 0.892753 | -1.99357 |
| 16 | 8 | 0 | -0.42589 | 3.657972 | 1.501108 |
| 17 | 6 | 0 | 1.814855 | 2.213726 | 0.459683 |
| 18 | 8 | 0 | 2.979169 | 1.655714 | 0.117628 |
| 19 | 6 | 0 | 3.006877 | 0.454703 | -0.61898 |
| 20 | 6 | 0 | 4.423047 | 0.128803 | -0.97354 |
| 21 | 6 | 0 | 4.766472 | -1.028434 | -0.42539 |
| 22 | 6 | 0 | 3.589731 | -1.519626 | 0.341938 |
| 23 | 8 | 0 | 2.574756 | -0.6215 | 0.203289 |
| 24 | 8 | 0 | 3.464332 | -2.520853 | 0.985343 |
| 25 | 6 | 0 | 6.039344 | -1.791391 | -0.46903 |
| 26 | 1 | 0 | -5.2553 | -1.895143 | -0.91748 |
| 27 | 1 | 0 | -5.11126 | -1.102224 | 0.643303 |
| 28 | 1 | 0 | -3.13132 | -3.177453 | -0.39259 |
| 29 | 1 | 0 | -4.40088 | -3.487877 | 0.778937 |
| 30 | 1 | 0 | -2.10712 | -2.961697 | 1.798648 |
| 31 | 1 | 0 | -3.42212 | -1.980224 | 2.396615 |
| 32 | 1 | 0 | -2.57317 | -0.170833 | -2.69459 |
| 33 | 1 | 0 | -2.63008 | -1.852332 | -2.1616 |
| 34 | 1 | 0 | -4.07039 | -1.081263 | -2.84582 |
| 35 | 1 | 0 | -4.00894 | 1.590869 | -1.5017 |
| 36 | 1 | 0 | -5.42906 | 0.555665 | -1.46669 |
| 37 | 1 | 0 | -4.76814 | 1.19324 | 0.045209 |
| 38 | 1 | 0 | -0.10673 | -1.400744 | 1.853102 |
| 39 | 1 | 0 | -0.49678 | 0.316376 | 1.969567 |
| 40 | 1 | 0 | -1.23377 | -0.834275 | 3.072034 |
| 41 | 1 | 0 | -1.97414 | 1.375328 | -1.31607 |
| 42 | 1 | 0 | 0.240421 | -0.212445 | -0.25494 |
| 43 | 1 | 0 | -0.02922 | 0.088009 | -2.4397 |
| 44 | 1 | 0 | 1.987838 | 3.088557 | 1.075571 |
| 45 | 1 | 0 | 2.335003 | 0.523209 | -1.47564 |
| 46 | 1 | 0 | 5.021722 | 0.779406 | -1.59308 |
| 47 | 1 | 0 | 5.86878 | -2.791632 | -0.87127 |
| 48 | 1 | 0 | 6.776856 | -1.282169 | -1.08633 |
| 49 | 1 | 0 | 6.443037 | -1.91056 | 0.538129 |

**Supplementary Data 2.27. Cartesian coordinates of the conformers 1ae−1je of 4*S*,5*R*,2'*R* isomer for ECD calculation (continued).**

Conformer 1me

| Center Number | Atomic Number | Atomic Type | Coordinates (Angstroms) X | Y | Z |
|---|---|---|---|---|---|
| 1 | 6 | 0 | 4.68563 | -0.932073 | -1.23838 |
| 2 | 6 | 0 | 5.458761 | -1.547582 | -0.08586 |
| 3 | 6 | 0 | 5.281012 | -0.702352 | 1.166684 |
| 4 | 6 | 0 | 3.93036 | -0.050903 | 1.321484 |
| 5 | 6 | 0 | 2.972219 | -0.096336 | 0.382439 |
| 6 | 6 | 0 | 3.176836 | -0.841086 | -0.94664 |
| 7 | 6 | 0 | 2.535124 | -0.112823 | -2.13676 |
| 8 | 6 | 0 | 2.60361 | -2.272006 | -0.87606 |
| 9 | 6 | 0 | 3.795651 | 0.694751 | 2.633357 |
| 10 | 6 | 0 | 1.696666 | 0.661101 | 0.653827 |
| 11 | 6 | 0 | 0.32492 | 0.022517 | 0.34508 |
| 12 | 6 | 0 | -0.37862 | 1.116724 | -0.39862 |
| 13 | 6 | 0 | 0.541592 | 2.236465 | -0.6176 |
| 14 | 8 | 0 | 1.724747 | 1.937041 | -0.06138 |
| 15 | 8 | 0 | -0.26659 | -0.32645 | 1.585633 |
| 16 | 8 | 0 | 0.35616 | 3.277126 | -1.20005 |
| 17 | 6 | 0 | -1.63536 | 1.121521 | -0.82987 |
| 18 | 8 | 0 | -2.41774 | 0.043611 | -0.63994 |
| 19 | 6 | 0 | -3.78435 | 0.190261 | -0.93638 |
| 20 | 6 | 0 | -4.43737 | -1.155504 | -0.96679 |
| 21 | 6 | 0 | -5.36706 | -1.21959 | -0.02429 |
| 22 | 6 | 0 | -5.3667 | 0.089724 | 0.686201 |
| 23 | 8 | 0 | -4.42797 | 0.895003 | 0.114603 |
| 24 | 8 | 0 | -6.04491 | 0.457007 | 1.600694 |
| 25 | 6 | 0 | -6.28615 | -2.316727 | 0.369273 |
| 26 | 1 | 0 | 5.066772 | 0.076131 | -1.43332 |
| 27 | 1 | 0 | 4.832288 | -1.512437 | -2.15335 |
| 28 | 1 | 0 | 6.519046 | -1.623711 | -0.33454 |
| 29 | 1 | 0 | 5.109851 | -2.566763 | 0.093176 |
| 30 | 1 | 0 | 6.020167 | 0.108295 | 1.177546 |
| 31 | 1 | 0 | 5.48766 | -1.29094 | 2.06608 |
| 32 | 1 | 0 | 1.447463 | -0.057957 | -2.06859 |
| 33 | 1 | 0 | 2.92166 | 0.902285 | -2.23641 |
| 34 | 1 | 0 | 2.767632 | -0.65693 | -3.05529 |
| 35 | 1 | 0 | 1.514109 | -2.285082 | -0.86576 |
| 36 | 1 | 0 | 2.922848 | -2.840338 | -1.75399 |
| 37 | 1 | 0 | 2.943886 | -2.798802 | 0.015292 |
| 38 | 1 | 0 | 4.76284 | 0.745823 | 3.133694 |
| 39 | 1 | 0 | 3.443698 | 1.718549 | 2.502675 |
| 40 | 1 | 0 | 3.108269 | 0.18653 | 3.314072 |
| 41 | 1 | 0 | 1.646277 | 0.923315 | 1.706857 |
| 42 | 1 | 0 | 0.410695 | -0.86752 | -0.27541 |
| 43 | 1 | 0 | -1.08997 | -0.786836 | 1.402926 |
| 44 | 1 | 0 | -2.07079 | 1.971844 | -1.34649 |
| 45 | 1 | 0 | -3.91088 | 0.766709 | -1.85534 |
| 46 | 1 | 0 | -4.14539 | -1.916775 | -1.67432 |
| 47 | 1 | 0 | -7.3235 | -1.983427 | 0.304861 |
| 48 | 1 | 0 | -6.15206 | -3.185572 | -0.27213 |
| 49 | 1 | 0 | -6.10284 | -2.609566 | 1.404715 |

Conformer 1ne

| Center Number | Atomic Number | Atomic Type | Coordinates (Angstroms) X | Y | Z |
|---|---|---|---|---|---|
| 1 | 6 | 0 | -4.63273 | -0.532724 | 1.479481 |
| 2 | 6 | 0 | -4.47014 | -2.022538 | 1.259705 |
| 3 | 6 | 0 | -4.41941 | -2.279275 | -0.2338 |
| 4 | 6 | 0 | -3.46595 | -1.359833 | -0.95609 |
| 5 | 6 | 0 | -2.96923 | -0.242044 | -0.40112 |
| 6 | 6 | 0 | -3.44056 | 0.295686 | 0.960537 |
| 7 | 6 | 0 | -3.95552 | 1.746416 | 0.866946 |
| 8 | 6 | 0 | -2.30571 | 0.247471 | 1.997309 |
| 9 | 6 | 0 | -3.08908 | -1.889598 | -2.31951 |
| 10 | 6 | 0 | -1.80485 | 0.413735 | -1.11366 |
| 11 | 6 | 0 | -0.40579 | -0.079269 | -0.65883 |
| 12 | 6 | 0 | 0.270649 | 1.194621 | -0.2593 |
| 13 | 6 | 0 | -0.62103 | 2.32654 | -0.52543 |
| 14 | 8 | 0 | -1.78517 | 1.856285 | -1.00097 |
| 15 | 8 | 0 | 0.203072 | -0.731639 | -1.76289 |
| 16 | 8 | 0 | -0.43303 | 3.507549 | -0.36605 |
| 17 | 6 | 0 | 1.485883 | 1.344911 | 0.257454 |
| 18 | 8 | 0 | 2.249446 | 0.269586 | 0.515599 |
| 19 | 6 | 0 | 3.583183 | 0.511552 | 0.890576 |
| 20 | 6 | 0 | 4.17911 | -0.738076 | 1.457589 |
| 21 | 6 | 0 | 5.198739 | -1.125427 | 0.704631 |
| 22 | 6 | 0 | 5.320589 | -0.14629 | -0.4114 |
| 23 | 8 | 0 | 4.356981 | 0.805635 | -0.26274 |
| 24 | 8 | 0 | 6.103667 | -0.119618 | -1.31539 |
| 25 | 6 | 0 | 6.112859 | -2.289086 | 0.820856 |
| 26 | 1 | 0 | -5.54017 | -0.205452 | 0.960326 |
| 27 | 1 | 0 | -4.77514 | -0.298154 | 2.537552 |
| 28 | 1 | 0 | -5.2948 | -2.573824 | 1.71581 |
| 29 | 1 | 0 | -3.5484 | -2.379271 | 1.729032 |
| 30 | 1 | 0 | -5.41923 | -2.169193 | -0.67185 |
| 31 | 1 | 0 | -4.11663 | -3.309961 | -0.44147 |
| 32 | 1 | 0 | -3.15684 | 2.474758 | 0.755436 |
| 33 | 1 | 0 | -4.64189 | 1.861335 | 0.025378 |
| 34 | 1 | 0 | -4.50237 | 1.98342 | 1.783634 |
| 35 | 1 | 0 | -1.45072 | 0.858246 | 1.702138 |
| 36 | 1 | 0 | -2.66388 | 0.638823 | 2.952461 |
| 37 | 1 | 0 | -1.94968 | -0.771942 | 2.159259 |
| 38 | 1 | 0 | -3.90463 | -2.502456 | -2.70772 |
| 39 | 1 | 0 | -2.88486 | -1.112889 | -3.05534 |
| 40 | 1 | 0 | -2.20407 | -2.528618 | -2.26061 |
| 41 | 1 | 0 | -1.8626 | 0.213146 | -2.17924 |
| 42 | 1 | 0 | -0.49063 | -0.774558 | 0.178374 |
| 43 | 1 | 0 | 1.040219 | -1.098326 | -1.46647 |
| 44 | 1 | 0 | 1.898765 | 2.321223 | 0.494689 |
| 45 | 1 | 0 | 3.636159 | 1.372248 | 1.560416 |
| 46 | 1 | 0 | 3.787203 | -1.204598 | 2.348742 |
| 47 | 1 | 0 | 7.149057 | -1.952031 | 0.884566 |
| 48 | 1 | 0 | 5.877797 | -2.88191 | 1.702665 |
| 49 | 1 | 0 | 6.031413 | -2.923053 | -0.06394 |

**Supplementary Data 2.27. Cartesian coordinates of the conformers 1ae−1je of 4*S*,5*R*,2'*R* isomer for ECD calculation (continued).**

Conformer 1oe

| Center Number | Atomic Number | Atomic Type | Coordinates (Angstroms) | | |
|---|---|---|---|---|---|
| | | | X | Y | Z |
| 1 | 6 | 0 | 3.06962 | -1.904024 | -1.38288 |
| 2 | 6 | 0 | 4.254471 | -2.272617 | -0.51095 |
| 3 | 6 | 0 | 3.842812 | -2.179815 | 0.946676 |
| 4 | 6 | 0 | 3.012542 | -0.963828 | 1.282758 |
| 5 | 6 | 0 | 2.441028 | -0.184322 | 0.351232 |
| 6 | 6 | 0 | 2.588056 | -0.460298 | -1.15543 |
| 7 | 6 | 0 | 3.605874 | 0.514411 | -1.77918 |
| 8 | 6 | 0 | 1.258242 | -0.324725 | -1.91358 |
| 9 | 6 | 0 | 2.899121 | -0.787819 | 2.776979 |
| 10 | 6 | 0 | 1.597922 | 0.98565 | 0.811718 |
| 11 | 6 | 0 | 0.062684 | 0.737302 | 0.870284 |
| 12 | 6 | 0 | -0.48666 | 1.881699 | 0.07854 |
| 13 | 6 | 0 | 0.633509 | 2.730626 | -0.36708 |
| 14 | 8 | 0 | 1.793825 | 2.17049 | 0.006662 |
| 15 | 8 | 0 | -0.41175 | 0.775377 | 2.212434 |
| 16 | 8 | 0 | 0.607601 | 3.759107 | -0.99693 |
| 17 | 6 | 0 | -1.71217 | 2.214738 | -0.34175 |
| 18 | 8 | 0 | -2.89826 | 1.62288 | -0.17671 |
| 19 | 6 | 0 | -2.99676 | 0.400367 | 0.516182 |
| 20 | 6 | 0 | -4.44031 | 0.050762 | 0.692287 |
| 21 | 6 | 0 | -4.70192 | -1.093994 | 0.07709 |
| 22 | 6 | 0 | -3.43674 | -1.55216 | -0.55879 |
| 23 | 8 | 0 | -2.45693 | -0.647654 | -0.27869 |
| 24 | 8 | 0 | -3.22372 | -2.533777 | -1.20925 |
| 25 | 6 | 0 | -5.96122 | -1.870286 | -0.04866 |
| 26 | 1 | 0 | 3.310901 | -2.024556 | -2.44237 |
| 27 | 1 | 0 | 2.243992 | -2.590497 | -1.16438 |
| 28 | 1 | 0 | 5.092756 | -1.599857 | -0.70794 |
| 29 | 1 | 0 | 4.601454 | -3.282147 | -0.74019 |
| 30 | 1 | 0 | 4.720405 | -2.189486 | 1.600043 |
| 31 | 1 | 0 | 3.259935 | -3.066505 | 1.22527 |
| 32 | 1 | 0 | 3.2578 | 1.543589 | -1.71708 |
| 33 | 1 | 0 | 4.571616 | 0.457643 | -1.27494 |
| 34 | 1 | 0 | 3.755541 | 0.264791 | -2.83323 |
| 35 | 1 | 0 | 0.867128 | 0.691723 | -1.91603 |
| 36 | 1 | 0 | 1.416982 | -0.604926 | -2.95754 |
| 37 | 1 | 0 | 0.493488 | -0.99082 | -1.50896 |
| 38 | 1 | 0 | 3.880898 | -0.554231 | 3.198317 |
| 39 | 1 | 0 | 2.200916 | -0.025952 | 3.112162 |
| 40 | 1 | 0 | 2.584873 | -1.734154 | 3.225448 |
| 41 | 1 | 0 | 1.897538 | 1.283818 | 1.811273 |
| 42 | 1 | 0 | -0.19154 | -0.224006 | 0.424551 |
| 43 | 1 | 0 | -0.21713 | -0.071603 | 2.623191 |
| 44 | 1 | 0 | -1.8259 | 3.118215 | -0.92936 |
| 45 | 1 | 0 | -2.43144 | 0.450071 | 1.447799 |
| 46 | 1 | 0 | -5.11561 | 0.678363 | 1.254039 |
| 47 | 1 | 0 | -5.82582 | -2.879493 | 0.344481 |
| 48 | 1 | 0 | -6.7723 | -1.385593 | 0.491416 |
| 49 | 1 | 0 | -6.24316 | -1.965782 | -1.09883 |
