# Supplementary material for: An ancestral function of strigolactones as symbiotic rhizosphere signals
Source: Nat Commun. 2022 Jul 8;13:3974. doi: 10.1038/s41467-022-31708-3 (PMC9270392; doi:10.1038/s41467-022-31708-3)
Supplement: Supplementary file 7 — Reporting Summary [file 41467_2022_31708_MOESM7_ESM.pdf]

nature portfolio

Corresponding author(s): Junko Kyozuka
Takahito Nomura
Pierre-marc Delaux

Last updated by author(s): Jun 15, 2022

Reporting Summary

Nature Portfolio wishes to improve the reproducibility of the work that we publish. This form provides structure for consistency and transparency in reporting. For further information on Nature Portfolio policies, see our Editorial Policies and the Editorial Policy Checklist.

Statistics

For all statistical analyses, confirm that the following items are present in the figure legend, table legend, main text, or Methods section.

n/a	Confirmed	
☐	☒	The exact sample size (*n*) for each experimental group/condition, given as a discrete number and unit of measurement
☐	☒	A statement on whether measurements were taken from distinct samples or whether the same sample was measured repeatedly
☐	☒	The statistical test(s) used AND whether they are one- or two-sided<br>*Only common tests should be described solely by name; describe more complex techniques in the Methods section.*
☐	☒	A description of all covariates tested
☐	☒	A description of any assumptions or corrections, such as tests of normality and adjustment for multiple comparisons
☐	☒	A full description of the statistical parameters including central tendency (e.g. means) or other basic estimates (e.g. regression coefficient) AND variation (e.g. standard deviation) or associated estimates of uncertainty (e.g. confidence intervals)
☐	☒	For null hypothesis testing, the test statistic (e.g. *F*, *t*, *r*) with confidence intervals, effect sizes, degrees of freedom and *P* value noted<br>*Give P values as exact values whenever suitable.*
☒	☐	For Bayesian analysis, information on the choice of priors and Markov chain Monte Carlo settings
☐	☒	For hierarchical and complex designs, identification of the appropriate level for tests and full reporting of outcomes
☒	☐	Estimates of effect sizes (e.g. Cohen's *d*, Pearson's *r*), indicating how they were calculated

Our web collection on statistics for biologists contains articles on many of the points above.

Software and code

Policy information about availability of computer code

Data collection	tblastn v2.9.0+ algorithm (The 1KP project)
Data analysis	MAFFT v7.313, IQ-TREE v1.6.7, ModelFinder and iTOL v5.6.3 (Phylogenetic analysis), MultiQuant (LC-MS/MS: AB Sciex), SigmaPlot 14 (Kinetics parameter: Systat Software), ImageJ (v2.0.0), Galaxy (RNA_seq analysis), R (Statistical analysis), Spartan'20 (NMR analysis: Wavefunction), Gaussian09 (NMR analysis: Gaussian Inc.)

For manuscripts utilizing custom algorithms or software that are central to the research but not yet described in published literature, software must be made available to editors and reviewers. We strongly encourage code deposition in a community repository (e.g. GitHub). See the Nature Portfolio guidelines for submitting code & software for further information.

Data

Policy information about availability of data

All manuscripts must include a data availability statement. This statement should provide the following information, where applicable:

- Accession codes, unique identifiers, or web links for publicly available datasets
- A description of any restrictions on data availability
- For clinical datasets or third party data, please ensure that the statement adheres to our policy

The accession numbers of genes analyzed in this study are shown in Supplementary Table 5. All reads obtained was deposited in the DDBJ and are available through the Sequence Read Archive (SRA) with the accession number DRA012476 [https://ddbj.nig.ac.jp/resource/bioproject/PRJDB12017]. LC-MS/MS data was deposited in Metabolights with the identifier MTBLS5064 [https://www.ebi.ac.uk/metabolights/MTBLS5064]. The authors declare that all data supporting the findings of this study are available within the manuscript and its supplementary files or are from the corresponding author upon request.

Field-specific reporting

Please select the one below that is the best fit for your research. If you are not sure, read the appropriate sections before making your selection.

☒ Life sciences ☐ Behavioural & social sciences ☐ Ecological, evolutionary & environmental sciences

For a reference copy of the document with all sections, see nature.com/documents/nr-reporting-summary-flat.pdf

Life sciences study design

All studies must disclose on these points even when the disclosure is negative.

Sample size	At least three replicates were performed in all experiments. In the gene expression analysis, more than 10 individuals were pooled to normalize the variation among individual materials. In the phenotyping, we also pooled more than 10 individuals and repeated the experiments at least three times.
Data exclusions	No data were excluded.
Replication	At least three replicates were performed in all experiments.
Randomization	More than 10 randomly selected individuals were pooled to normalize the variation.
Blinding	Blinding was not relevant and required for our analysis because we used plant materials from different genetic backgrounds.

Reporting for specific materials, systems and methods

We require information from authors about some types of materials, experimental systems and methods used in many studies. Here, indicate whether each material, system or method listed is relevant to your study. If you are not sure if a list item applies to your research, read the appropriate section before selecting a response.

Materials & experimental systems

n/a	Involved in the study	
☒	☐	Antibodies
☒	☐	Eukaryotic cell lines
☒	☐	Palaeontology and archaeology
☒	☐	Animals and other organisms
☒	☐	Human research participants
☒	☐	Clinical data
☒	☐	Dual use research of concern

Methods

n/a	Involved in the study	
☒	☐	ChIP-seq
☒	☐	Flow cytometry
☒	☐	MRI-based neuroimaging
